# Decoupling the Transparency‐Efficiency Trade‐Off in Semi‐Transparent Organic Solar Cells via Optimized Dual‐Channel Photoelectric Conversion

**DOI:** 10.1002/advs.202523474

**Published:** 2026-03-14

**Authors:** Yuyan Li, Shibo Wang, Heng Liu, Bonan Shi, Zhuoqiong Zhang, Yabing Tang, Yuting Song, Shu Kong So, Dongbin Dang, Han Yan, Xinhui Lu, Guilong Cai, Suojiang Zhang

**Affiliations:** ^1^ School of Chemical and Molecular Sciences Henan University (Zhengzhou) Zhengzhou China; ^2^ Longzihu New Energy Laboratory Zhengzhou China; ^3^ Beijing Key Laboratory of Solid State Battery and Energy Storage Process Institute of Process Engineering Chinese Academy of Sciences Beijing China; ^4^ Department of Physics The Chinese University of Hong Kong Hong Kong China; ^5^ State Key Laboratory for Mechanical Behavior of Materials, School of Materials Science and Engineering Xi'an Jiaotong University Xi'an China; ^6^ Department of Physics and Institute of Advanced Materials Hong Kong Baptist University Hong Kong China

**Keywords:** dual‐additive approach, dual‐channel photoelectric conversion, low‐donor‐content device, semi‐transparent organic solar cell, spontaneously formed photo‐charge

## Abstract

The development of semi‐transparent organic solar cells (ST‐OSCs) for building‐integrated photovoltaics is fundamentally constrained by the inherent trade‐off between transparency and efficiency. To achieve a breakthrough, it is imperative to maintain high transparency while mitigating the concomitant efficiency loss in low‐donor‐content devices. Herein, we address this challenge by implementing a strategy that optimizes dual‐channel photoelectric conversion, which synergistically integrates the respective advantages of both the heterojunction (HJ) channel and the spontaneously formed photo‐charge (SP) channel. The results reveal that the HJ channel primarily governs hole transport and thus the fill factor, whereas the SP channel is pivotal for charge generation, directly influencing the short‐circuit current density. Strategic acceptor selection and dual‐additive‐assisted morphology control effectively minimize electrical losses from insufficient charge generation and severe recombination, enabling a remarkable power conversion efficiency of 11.3% in PTB7‐Th:BTP‐eC9 (1:4) devices that outperforms their bulk heterojunction (BHJ) counterparts (10.4%), without losing the high transparency (>65%). The general applicability of this strategy was further validated in PM6:BTP‐eC9 (1:3) based ST‐OSCs, yielding a competitive light utilization efficiency of 4.67% and demonstrating the generalizability of our approach across different active layer systems. This study reveals the crucial role of dual‐channel photoelectric conversion in realizing high‐performance ST‐OSCs.

## Graphical abstract

The respective roles of the heterojunction (HJ) and spontaneously formed photo‐charge (SP) channels in low‐donor‐content devices have been decoupled and subjected to a targeted optimization strategy. A breakthrough of light utilization efficiency (LUE) in semi‐transparent organic solar cells (ST‐OSCs) is achieved.

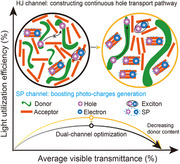

## 1 Introduction

The multi functionality of optical transparency, heat insulation, and power generator in semi‐transparent organic solar cells (ST‐OSCs) has established this technology as a cornerstone for building‐integrated photovoltaics (BIPV) applications [1, 2, 3, 4, 5]. Benefit from elaborate active layer control, optical engineering design, and electrode technology innovation, the champion light utilization efficiency (LUE) (defined as the product of average visible transmittance (AVT) and power conversion efficiency (PCE)) of ST‐OSCs has surpassed 5.0% [6, 7, 8, 9, 10]. Nevertheless, the inherent trade‐off between transparency and efficiency continues to impede further advancement. In state‐of‐the‐art ST‐OSCs, the active layer typically consists of a low proportion of electron‐donor (D) and a high proportion of electron‐acceptor (A) [7, 8, 9, 11, 12]. This unbalanced D/A ratio ensures high AVT albeit at the loss of PCE. To further improve the LUE of ST‐OSCs, innovative strategies for reducing the PCE loss from bulk heterojunction (BHJ) devices to low‐donor‐content devices are urgently be developed.

Progressively decreasing the donor content inevitably leads to insufficient charge generation and severe charge recombination, significantly impairing both short‐circuit current density (*J*_SC_) and fill factor (FF) of photovoltaic devices [13, 14]. The conventional mechanisms posits that photogenerated excitons (EXs) must first dissociate at the D/A heterojunction, after which the resulting free holes are transported through the continuous donor matrix [15]. The HJ assisted photoelectric conversion channel creates a dilemma that improving one property often compromises the other. Specifically, conversing aggregated donor phases into polydisperse donor phases will provide abundant D/A heterojunctions for EX dissociation, but it simultaneously disrupts the continuity of the hole transport pathways and introduces more hole recombination centers. In the pursuit of higher PCE in low‐donor‐content devices, researchers have employed strategies such as using semi‐transparent hole‐transporting material to partially replace the donor or promoting donor self‐aggregation to suppress charge recombination [16, 17, 18]. However, these approaches often come at the cost of further aggravating the inherent issue of insufficient charge generation. Encouragingly, the discovery of spontaneous photo‐charge formation in Y‐series acceptors reveals that EXs can dissociate without strict reliance on D/A heterojunctions, and that free holes can also transport through the acceptor phase [19, 20, 21, 22, 23, 24, 25]. The photo‐charge (SP) assisted photoelectric conversion channel offers a promising avenue to overcome the longstanding challenge between charge generation and recombination. Theoretically, an ideal acceptor with near‐unity charge generation efficiency and negligible charge recombination, could comprehensively resolve the persistent challenges of insufficient charge generation and severe charge recombination in low‐donor‐content devices. In practice, however, although Y‐series acceptors exhibit efficient SP generation at low temperatures due to strong quadrupole moments and polarization effects, at room temperature, thermal energy causes reversion of the SP state to the lowest singlet excited (LE) state, hampering charge generation [26, 27]. Furthermore, the lack of spatial separation between holes and electrons within the acceptor bulk leads to severe charge recombination [22]. While strategies such as regulating crystalline morphology and electronic structures have shown promise in optimizing the SP channel, OSCs still cannot work just as the inorganic solar cells, as evidenced by the persistently low PCE of single‐component OSCs [21, 23, 24, 28, 29]. Thus, relying solely on the SP channel remains insufficient to fully mitigate electrical losses in low‐donor‐content devices as well. A more promising strategy currently lies in adopting a compromise solution that leverages the respective advantages of both HJ and SP assisted photoelectric conversion channels.

Herein, we propose the optimization of dual‐channel photoelectric conversion as an efficient strategy to address the PCE loss issue in low‐donor‐content devices. Photovoltaic testing of two representative electron‐acceptors with distinct SP characteristics revealed that the SP channel predominantly influences *J*_SC_, whereas the HJ channel mainly modulates FF. Ternary phase diagram analysis and morphology characterization further identified that simultaneous optimization of both the SP and HJ channels hinges on improving the SP characteristics of the electron‐acceptors and forming continuous donor phases. Consequently, we employed a dual‐additive strategy involving trichlorobenzene (TCB) and 1,8‐diiodooctane (DIO) for synergistic morphology control, combined with a rational acceptor selection. This approach enabled the fabrication of PTB7‐Th:BTP‐eC9 (1:4, w/w) devices that achieved a PCE of 11.3%, surpassing that of conventional BHJ counterparts (10.4%), while preserving a high AVT of 65.4% compared to the pre‐optimization value of 65.8%. Furthermore, by leveraging this dual‐channel photoelectric optimization, the strategy was successfully applied to the PM6:BTP‐eC9 (1:3, w/w) semi‐transparent devices. These devices attained a competitive LUE of 4.67%, accompanied by an AVT of 34.3% and a PCE of 13.6%. This work demonstrates the feasibility of synergistically optimizing dual‐channel photoelectric conversion and establishes a comprehensive material and morphological blueprint for high‐performance, low‐donor‐content OSCs, thereby paving the way for the practical application of ST‐OSCs in next‐generation transparent energy systems.

## 2 Results and Discussion

### 2.1 Identifying the Distinct Photoelectric Conversion Processes in the Two Material Systems

**FIGURE 1:**
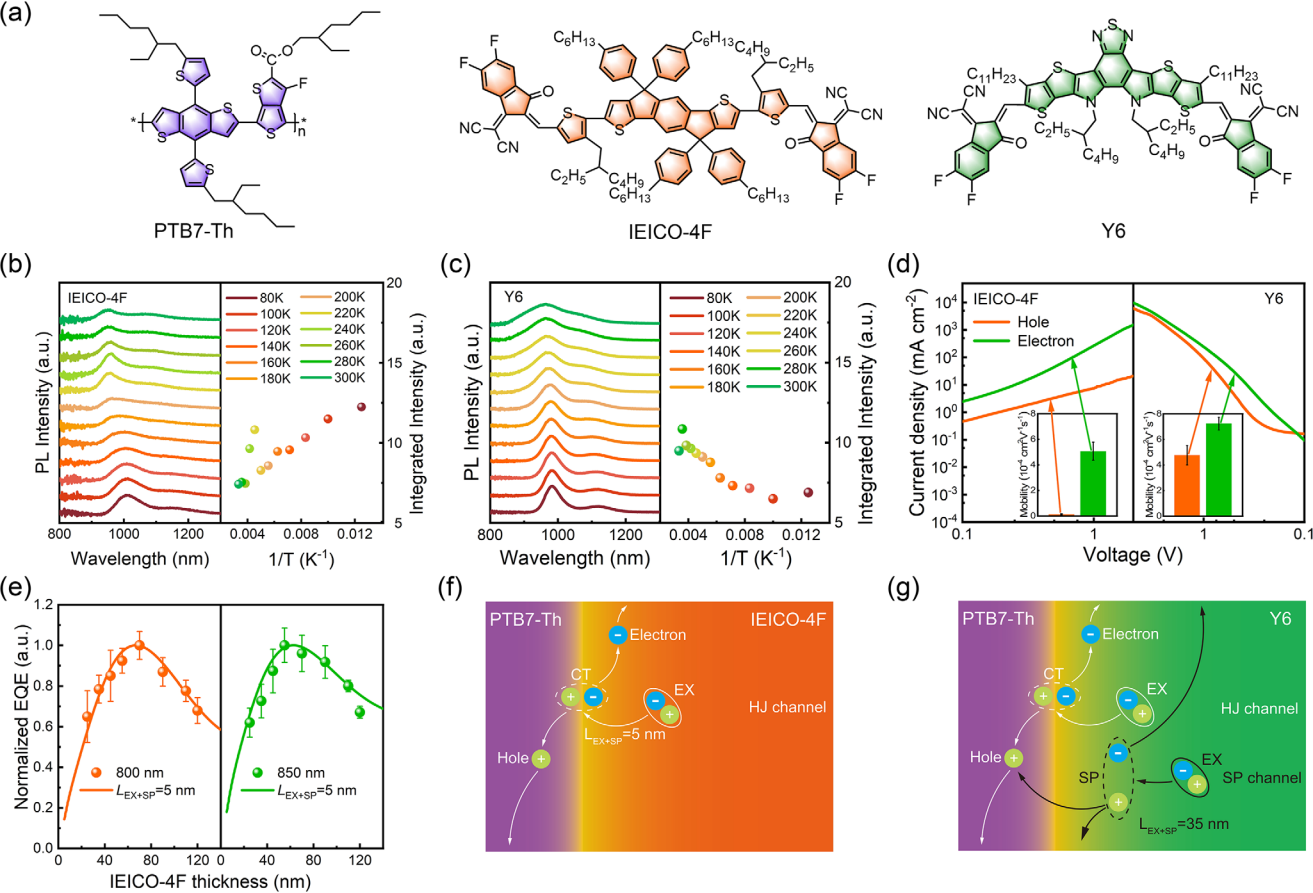
(a) The chemical structures of PTB7‐Th, IEICO‐4F, and Y6. Temperature‐dependent PL spectra (left) and corresponding integrated intensity plots (right) of (b) IEICO‐4F excited at 690 nm and (c) Y6 excited at 750 nm. (d) Hole‐only current density and electron‐only current density versus voltage curves of IEICO‐4F (left) and Y6 (right) devices. The insets show the statistical distribution of the average hole and electron mobilities. (e) *L*_EX+SP_ values from the thickness varying EQE measurements of the copper(I) thiocyanate (CuSCN)/IEICO‐4F devices. The excitation wavelength keeps at 800 and 850 nm. The experimental data (circles) are fitted (solid lines) for all IEICO‐4F thicknesses. The scheme of distinguishing photoelectric conversion processes in the acceptor phases of (f) PTB7‐Th:IEICO‐4F and (g) PTB7‐Th:Y6 devices.

To elucidate the respective roles of HJ and SP channels on specific photovoltaic parameters in low‐donor‐content devices, two material systems PTB7‐Th: IEICO‐4F and PTB7‐Th: Y6 were chosen for subsequent investigation (Figure 1a). We chose IEICO‐4F and Y6 as electron‐acceptors owing to their entirely distinct SP characteristics. For the electron‐donor, we chose PTB7‐Th because the PTB7‐Th:Y6 blend shows a higher SP channel contribution than the PM6:Y6 system, thus offering a more distinct view of the SP channel's function [30].

We first qualitatively accessed the SP yield in pure acceptor films using temperature‐dependent photoluminescence (PL) spectroscopy, where variations in integrated PL intensity reflect transitions between the SP and LE states [27]. In the IEICO‐4F film, the integrated PL intensity decreases with increasing temperature (Figure 1b), indicating that EXs dominate at low temperatures, and only a small fraction of EXs dissociate into SP at room temperature under thermal activation. In contrast, Y6 exhibits entirely opposite behavior. Its integrated PL intensity increases with rising temperature (Figure 1c), reflecting a high SP yield even under partial thermal‐driven SP→LE reversion at room temperature. Then, space charge limited current (SCLC) mobility measurements were employed to evaluate the SP transport properties. IEICO‐4F displays highly unbalanced charge transport with negligible hole mobility (*µ*_h_ = 0.1 × 10^−4^ cm^2^V^−1^s^−1^) and moderate electron mobility (*µ*_e_ = 5.1 × 10^−4^ cm^2^V^−1^s^−1^). In comparison, Y6 exhibits both high and balanced hole and electron mobilities (*µ*_h_ = 4.8 × 10^−4^ cm^2^V^−1^s^−1^ and *µ*_e_ = 7.2 × 10^−4^ cm^2^V^−1^s^−1^), supporting efficient hole transport within the acceptor phase (Figure 1d; Table S1). Owing to its low SP yield and limited hole transport, IEICO‐4F exhibits a short EX‐SP mixed transport length (*L*_EX+SP_, with contribution predominantly from exciton diffusion) of only 5 nm, as derived from thickness‐dependent external quantum efficiency (EQE) measurements (Figure 1e; Figure S1) [31, 32]. This value contrasts sharply with the ∼35 nm *L*_EX+SP_ previously reported for Y6 [23, 31]. Given that the *L*_EX_ of Y6 is only 11 nm, its high *L*_EX+SP_ must therefore be attributed primarily to SP transport. Such a pronounced difference leads to fundamentally distinct photoelectric conversion processes in the two systems. In PTB7‐Th:IEICO‐4F devices, the HJ channel dominates, with only EXs within 5 nm of the D/A heterojunction contributing to photocurrent, and hole transport confined mainly to donor phases (Figure 1f). While in PTB7‐Th:Y6 devices, both HJ and SP channels operate effectively. The long *L*_EX+SP_ enables efficient dissociation of most excitons into SP states, and the acceptor phase serves concurrently as a hole transport pathway and recombination zone (Figure 1g). While the high ionization energy (IE) offsets between PTB7‐Th and IEICO‐4F (0.5 eV) as well as Y6 (0.7 eV) guarantee efficient exciton splitting at the D/A heterojunction (Figure S2) [33].

### 2.2 Evaluating the Impact of HJ and SP Channels on Photovoltaic Parameters

**FIGURE 2:**
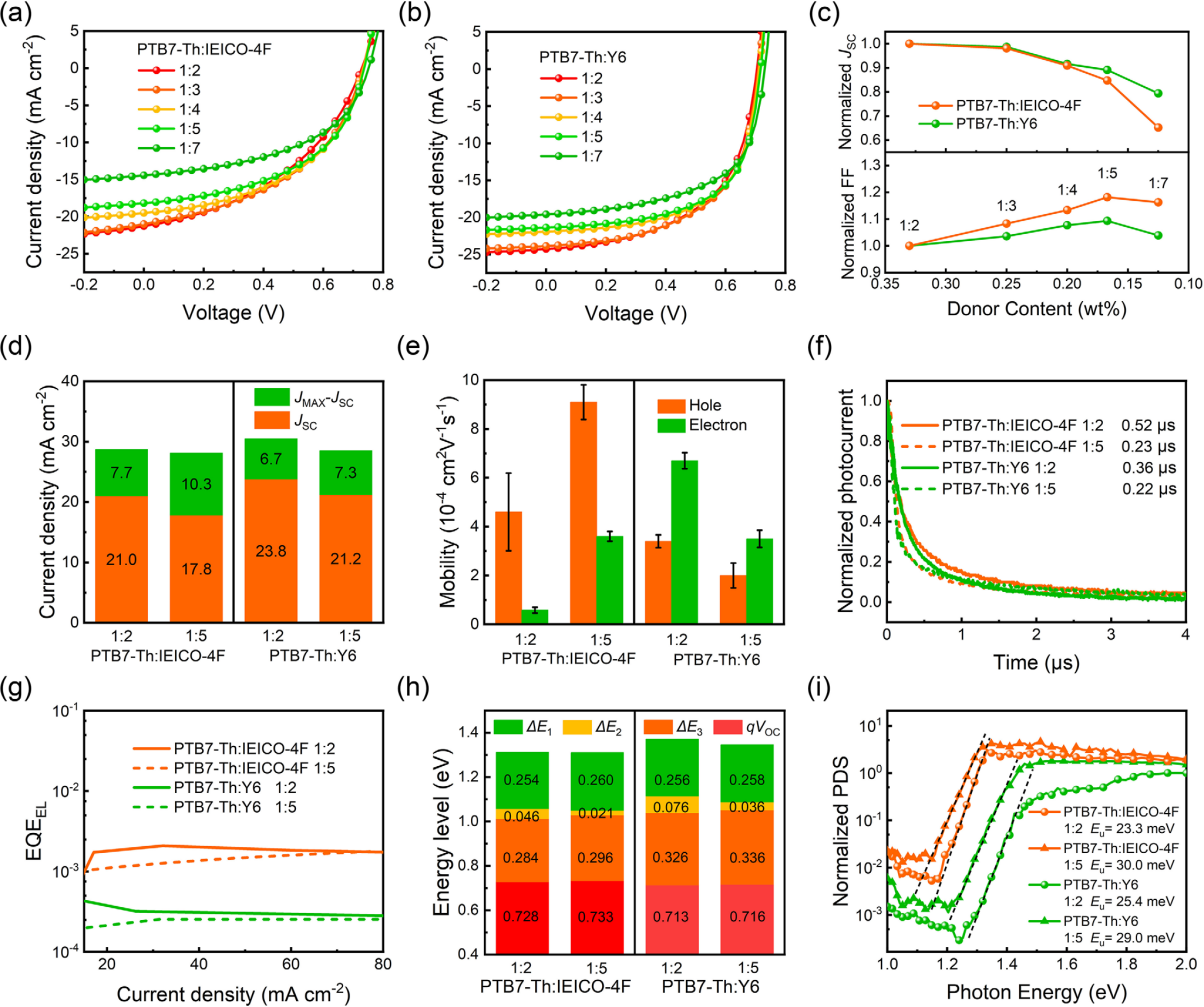
The *J*‐V curves of (a) PTB7‐Th:IEICO‐4F and (b) PTB7‐Th:Y6 devices at various D/A ratios (1:2, 1:3, 1:4, 1:5, and 1:7). (c) The normalized *J*_SC_ and FF of PTB7‐Th:IEICO‐4F and PTB7‐Th:Y6 devices at various D/A ratios (1:2, 1:3, 1:4, 1:5, and 1:7). (d) Histograms of the current density loss, (e) summarized hole and electron mobilities, (f) TPC decay kinetics curves, (g) EQE_EL_ measurements, (h) histograms of the energy loss (*∆E*_1_ represents the radiative recombination loss above *E*_g_, *∆E*_2_ represents the additional radiative recombination loss below *E*_g_, *∆E*_3_ represents the nonradiative recombination loss), and (i) PDS spectra of PTB7‐Th:IEICO‐4F and PTB7‐Th:Y6 devices at D/A ratios of 1:2 and 1:5.

Building on the distinguishing photoelectric conversion processes in the selected two material systems, we proceeded to evaluate their specific impacts on the photovoltaic performance of low‐donor‐content devices. Summarizing the detail data including open‐circuit voltage (*V*_OC_), *J*_SC_, and FF in Table S2, it is evident that both material systems exhibit similar variation trends in these parameters with decreasing donor content, though to differing extents (Figure 2a,b). Theoretically, the SP channel directly influences the charge generation and thus affects *J*_SC_ values. We therefore began by analyzing the variation in *J*_SC_. Evidently, PTB7‐Th:Y6 devices exhibit a higher initial *J*_SC_ and retain a greater proportion of *J*_SC_ with decreasing donor content (Figure 2c), a trend further supported by the corresponding external quantum efficiency (EQE) measurements (Figure S3 and Table S2). To include the influence of absorption, we calculated the theoretical maximum current density (*J*_MAX_, defined as the current density achievable under full photon utilization in the absence of electrical losses) by the transfer‐matrix model (Figures S4 and S5) [34, 35]. The current density loss was then quantified as the difference (*J*_MAX_—*J*_SC_) (Table S3). It was found that PTB7‐Th:Y6 (1:2) devices exhibit a higher *J*_MAX_ (30.5 vs. 28.7 mA cm^−2^) and a lower current density loss (6.7 vs. 7.7 mA cm^−2^), leading to a higher initial *J*_SC_ (23.8 mA cm^−2^) compared to PTB7‐Th:IEICO‐4F (1:2) devices (21.0 mA cm^−2^). When the D:A ratio shifts to 1:5 where the product of PCE for the opaque device and AVT for the blend film reaches its maximum (Figure S6 and Table S4), both of *J*_MAX_ and *J*_SC_ are decreased. The superior retention of *J*_SC_ in PTB7‐Th:Y6 (1:5) devices can be attributed to their lower current density loss (7.3 vs. 10.3 mA cm^−2^), which is consistent with their more pronounced reduction in *J*_MAX_ (2.0 vs. 0.6 mA cm^−2^) (Figure 2d). Based on this evidence, we conclude that the SP channel reduces the dependence of charge generation on the D/A heterojunctions, thus emerging as the key factor in controlling the *J*_SC_ decay at low donor contents.

In addition to its influence on *J*_SC_, the SP channel also introduces an additional hole transport pathway, thereby potentially affecting the variation in FF. Interestingly, the FF initially increases and then decreases as the donor content is reduced, peaking at a D:A ratio of 1:5 (Figure 2c). The significant difference lies in the extent of improvement. PTB7‐Th:IEICO‐4F devices show a more pronounced enhancement in FF (from 42.0% to 49.6%) compared to PTB7‐Th:Y6 devices (from 55.5% to 60.6%) as the D:A ratio shifts from 1:2 to 1:5. In‐depth analysis based on SCLC mobility measurements reveals that the variation in FF is primarily governed by the balance between hole and electron transport. In PTB7‐Th:IEICO‐4F devices, which exhibit more efficient hole transport, the hole/electron mobility ratio (*µ*_h_/*µ*_e_) is decreased from 7.7 to 2.5. In contrast, PTB7‐Th:Y6 devices, characterized by higher electron mobility, show an increase in *µ*_h_/*µ*_e_ from 0.5 to 0.6 (Figure 2e; Figure S7, Table S5). The more balanced carrier transport in PTB7‐Th:Y6 devices underlies their higher baseline FF, though with higher bimolecular recombination rate (2.3×10^−16^ vs. 2.0×10^−17^ m^3^s^−1^) as evidenced by drift‐diffusion simulation (Figure S8, Tables S6 and S7), while the smaller change in *µ*_h_/*µ*_e_ accounts for their more modest FF enhancement. This interpretation was further validated by transient photocurrent (TPC) measurements, which quantitatively evaluate the charge extraction time (*τ*_TPC_). The lower initial *τ*_TPC_ (0.36 vs. 0.52 µs) and the smaller extent of its reduction (0.14 vs. 0.29 µs) of PTB7‐Th:Y6 devices correlate well with the trends in FF and *µ*_h_/*µ*_e_ variation (Figure 2f), underscoring the significance of carrier mobility balance. Considering the continuous decrease in hole mobility with reduced donor content in PTB7‐Th:Y6 devices (Figure S9 and Table S8), we speculate that hole transport occurs predominantly through the donor phases, even in the presence of the SP channel [36, 37]. Therefore, achieving FF enhancement ultimately depends on balancing hole transport in the donor phases and electron transport in the acceptor phases, which aligns with the mechanism governed exclusively by the HJ channel.

Finally, energy loss analysis was employed to investigate the *V*_OC_ variation [38]. After evaluating the *∆E*_3_ through measuring electroluminescence quantum efficiency (EQE_EL_, Figure 2g), we summarized all three parts of energy loss in Figure 2h and Table S9. It was found that PTB7‐Th:IEICO‐4F (1:2) devices, despite having a narrower optical bandgap (*E*_g_ = 1.312 vs. 1.371 eV) (Figure S10), exhibit a higher *V*_OC_ (0.726 vs. 0.712 V) compared to PTB7‐Th:Y6 (1:2) devices. This unconventional observation is primarily attributed to the larger *∆E*_3_ in PTB7‐Th:Y6 (1:2) devices (0.326 vs. 0.284 eV), which results from their greater energy disorder. To corroborate this hypothesis, we employed photothermal deflection spectroscopy (PDS) to determine the Urbach energy (*E*_u_) as a quantitative measure of energy disorder (Figure 2i). The results confirm a higher *E*_u_ value in PTB7‐Th:Y6 (1:2) devices (25.4 vs. 23.3 meV), which is consistent with the trend observed in *∆E*_3_. The intrinsic energy disorder of neat Y6 is significantly lower than that of IEICO‐4F (32.1 vs. 43.1 meV in the high‐temperature regime, as derived from the slope of *E*_0‐0_ vs 1/T plots based on temperature‐dependent PL spectroscopy, Figure S11). Given this contrast, we speculate that the higher *E*_u_ in the PTB7‐Th:Y6 blend arises from greater torsional disorder of the polymer donor or less ordered molecular stacking of the blend film [39, 40]. When the D:A ratio shifts to 1:5, the polymer donor possesses greater spatial freedom, leading to an increase in *E*_u_ and a corresponding rise in *∆E*_3_ in both material systems. Nevertheless, the *V*_OC_ still increases slightly, which can be attributed to the reduction in *∆E*_2_ resulting from a decreased number of charge‐transfer (CT) states [38]. Benefiting from a well‐balanced interplay among the three photovoltaic parameters, the final PCE of both material systems remains nearly unchanged across D:A ratios from 1:2 to 1:5 (Table S2).

### 2.3 Morphology Prediction and Characterization

**FIGURE 3:**
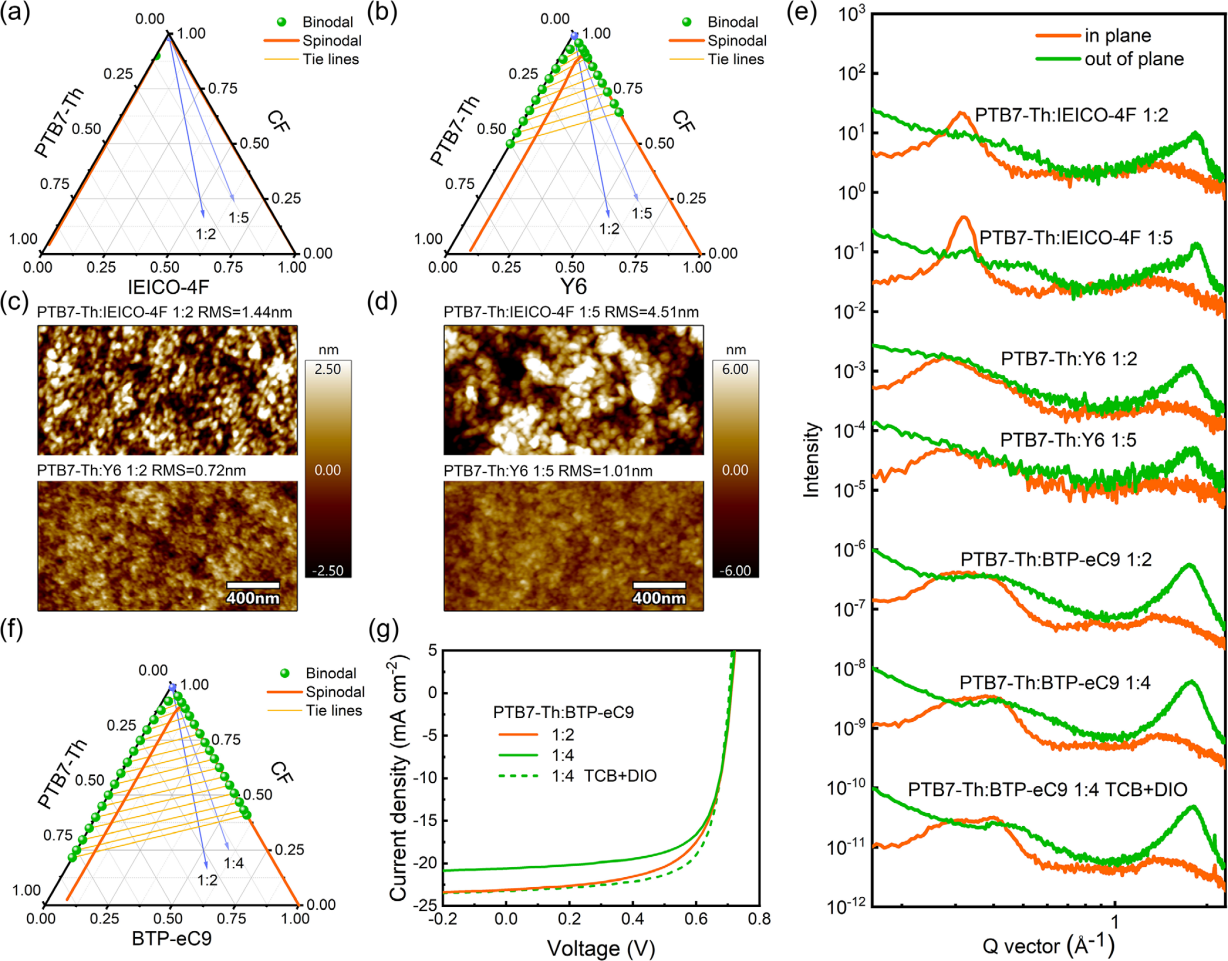
Ternary phase diagrams of (a) CF/PTB7‐Th/IEICO‐4F, and (b) CF/PTB7‐Th/Y6. The binodal line demarcates the boundary between the single‐phase and metastable regions, whereas the spinodal line separates the metastable region from the unstable region. Tie lines connect compositions with equal chemical potential. The bule points are the initial composition points at D/A ratios of 1:2 and 1:5. The blue arrows indicate the direction of solvent quenching at D/A ratios of 1:2 and 1:5. AFM images of PTB7‐Th:IEICO‐4F and PTB7‐Th:Y6 blend films at D/A ratios of (c) 1:2 and (d) 1:5. (e) In‐plane and out‐of‐plane line‐cut profiles of the GIWAXS measurements are presented for the following pristine blend films: PTB7‐Th:IEICO‐4F (at D/A ratios of 1:2 and 1:5), PTB7‐Th:Y6 (1:2 and 1:5), PTB7‐Th:BTP‐eC9 (1:2 and 1:4), as well as for the PTB7‐Th:BTP‐eC9 (1:4) blend film processed with the additives TCB and DIO. (f) Ternary phase diagram of CF/PTB7‐Th/BTP‐eC9. The blue arrows indicate the direction of solvent quenching at D/A ratios of 1:2 and 1:4. (g) The *J*‐V curves of PTB7‐Th:BTP‐eC9 (1:2) devices without additives, and PTB7‐Th:BTP‐eC9 (1:4) devices both with and without dual‐additive treatment.

Following an analysis of photovoltaic parameter variations from a device physics perspective, the investigation proceeded to their morphological origins. The thermodynamic equilibrium morphology of blend films is governed by material miscibility [41, 42]. Thus, the donor‐acceptor miscibility was evaluated qualitatively using the difference in their Hildebrand solubility parameters (*∆δ*_Hildebrand_), which were derived from contact angle measurements (Figure S12) [43, 44, 45]. The results showed that the *∆δ*_Hildebrand_ between PTB7‐Th and IEICO‐4F (9.3 Mpa^1/2^) was significantly larger than that between PTB7‐Th and Y6 (1.2 Mpa^1/2^), indicating worse miscibility and thus a strong tendency for larger phase separation in PTB7‐Th:IEICO‐4F blends (Table S10). Differential scanning calorimetry (DSC) measurements based on the melting point depression method provided further verification of this miscibility sequence [46, 47]. The results showed a higher Flory‐Huggins interaction parameter between PTB7‐Th and IEICO‐4F (*χ*_PTB7‐Th:IEICO‐4F‐DSC_ = 0.93) than between PTB7‐Th and Y6 (*χ*_PTB7‐Th:Y6‐DSC_ = 0.72) (Figure S13). This also leads to fewer CT states and hence a lower *ΔE*_2_ in the PTB7‐Th:IEICO‐4F devices (Figure 2h; Table S9), which serves as a minor contributing factor in explaining the counterintuitive observation in *V*_OC_. However, since the actual morphology of blend films is often kinetically frozen prior to reaching thermodynamic equilibrium, and miscibility alone cannot explain the morphological variations across different D:A ratios, we subsequently performed a more comprehensive analysis by calculating the ternary phase diagram using Flory‐Huggins theory [43, 44, 48]. The molecular weight of PTB7‐Th is 70 kDa. All relevant parameters are summarized in Tables S11 and S12, and the calculation detail is provided in the Supporting Information. The final phase separation morphology of the blend film is governed by the interplay between polymer chain mobility and the duration of phase separation. Higher polymer chain mobility combined with longer phase separation time generally leads to larger domain sizes and more ordered molecular stacking [44, 48]. In the chloroform (CF)/PTB7‐Th/IEICO‐4F ternary diagram, the high Flory‐Huggins interaction parameter between PTB7‐Th and IEICO‐4F (*χ*_PTB7‐Th/IEICO‐4F_ = 3.16) places the initial composition points below both the binodal and spinodal lines (Figure 3a), indicating that the spinodal liquid‐liquid (L‐L) demixing occurred already in the initial solution state. On this occasion, the final phase separation morphology is influenced more strongly by polymer chain mobility than phase separation time. The considerable disparity in the tie lines, with the left side being significantly lower than the right, indicates a lower solvent affinity for the polymer, suppressing its chain mobility. Subsequently, reducing the donor content lowers the blend solution viscosity, thereby enhancing the polymer chain mobility and ultimately leading to a larger domain size as well as more ordered molecular stacking [49]. In contrast, in the CF/PTB7‐Th/Y6 ternary diagram, the low *χ*_PTB7‐Th/Y6_ (0.39) results in a downward shift of both the binodal and spinodal lines, situating the initial composition points above these boundaries (Figure 3b). Meanwhile, though the left side of the tie lines remain lower than the right side, its shallower slop indicates lower viscosity and thus higher polymer chain mobility. Therefore, the final phase separation morphology is determined by the competition of polymer chain mobility and phase separation time. Polymer chain mobility also increases with decreasing donor content. The onset of phase separation can be inferred from the intersection of the solvent quenching line with the binodal line, whereas the endpoint is determined by the film formation time. Owing to similar initiation times and a reduction in film formation time resulting from lower viscosity, the total duration accessible for phase separation becomes shorter as the donor content decreases [49]. Therefore, the increased chain mobility promotes a larger domain size, whereas the shortened phase separation time partially impedes this process, leading to a decrease in molecular order.

The absorption spectra of the blend solutions and their corresponding films were measured to further support the aforementioned conclusions. At a fixed D:A ratio of 1:2, PTB7‐Th:IEICO‐4F exhibits a significantly smaller redshift (50 nm) in the absorption peak from solution to film compared to PTB7‐Th:Y6 (80 nm), indicating stronger pre‐aggregation in the solution state of the former blend (Figure S14) [50]. This finding well supports the observed differences in the initial composition points between the two material systems. Film formation time was determined from in situ absorption measurements as the interval between the moment the absorption signal first peaks after solution deposition (start) and the onset of the film stabilization region (end). A clear reduction in this time was observed for both blend systems when the D/A ratio changes from 1:2 to 1:5 from 0.55s to 0.5s for PTB7‐Th:IEICO‐4F and from 0.5s to 0.4s for PTB7‐Th:Y6, providing direct experimental verification (Figure S15). We then evaluated the domain size of the blend films by measuring the average fibril width using tapping‐mode atomic force microscopy (TM‐AFM, Figure 3c,d) [51, 52]. The results show that the average fibril width of PTB7‐Th:IEICO‐4F increases markedly from 58.2 to 115.9 nm as the D:A ratio changes from 1:2 to 1:5, whereas that of PTB7‐Th:Y6 increases only slightly from 41.5 to 77.5 nm (Figure S16). The combination of a higher average fibril width and a lower current density loss in PTB7‐Th:Y6 (1:5) devices compared to PTB7‐Th:IEICO‐4F (1:2) devices also rules out heterojunction area variation as the main cause of *J*_SC_ decay, supporting the role of the SP channel as the dominant factor. A similar trend was observed in the variation of the root‐mean‐square (RMS) roughness. Specifically, PTB7‐Th:IEICO‐4F not only exhibits a higher initial RMS value (1.44 vs. 0.72 nm) but also a much greater increase in roughness (3.07 vs. 0.29 nm) upon adjusting the D:A ratio from 1:2 to 1:5. These results confirm that the overall domain size of PTB7‐Th:IEICO‐4F blend films is larger than that of PTB7‐Th:Y6, and that the extent of domain size increase with lower donor content is more pronounced in PTB7‐Th:IEICO‐4F. Furthermore, the molecular stacking behavior was characterized using grazing incidence wide‐angle X‐ray scattering (GIWAXS) measurement (Figure 3e; Figure S17) [53]. The results show that all films exhibit a predominant face‐on orientation, as evidenced by the peak intensity distribution along the in‐plane and out‐of‐plane directions. Detailed analysis of the location, coherence length (CL), and area of characteristic π‐π stacking peaks provides quantitative insight into molecular packing order (Table S13). Upon changing the D:A ratio from 1:2 to 1:5, the π‐π stacking peak position moves to the right in both systems (from 1.839 to 1.858 Å^−^^1^ for PTB7‐Th:IEICO‐4F and from 1.755 to 1.774 Å^−^^1^ for PTB7‐Th:Y6), indicating tighter molecular stacking. Differently, PTB7‐Th:IEICO‐4F exhibits a simultaneous increase in both CL (from 2.85 to 3.09 nm) and peak area (from 1.01 to 1.56), while PTB7‐Th:Y6 shows a simultaneous decrease in CL (from 2.13 to 2.11 nm) and area (from 1.89 to 0.70). This opposite trend in the CL and area of the π‐π stacking peaks aligns well with the conclusions of the ternary phase diagram. The higher CL of the polymer's lamellar stacking in PTB7‐Th:IEICO‐4F also correlates well with its stronger pre‐aggregation behavior and lower *E*_u_.

Based on the above analysis, we attribute the distinct charge transport behavior between the two material systems to their morphological differences. At a D/A ratio of 1:2, the polymer donor in the PTB7‐Th:IEICO‐4F blend forms more continuous phases than that in the PTB7‐Th:Y6 blend, leading to higher hole mobility. When the D/A ratio changes to 1:5, the combination of more continuous donor phases and enhanced molecular ordering in PTB7‐Th:IEICO‐4F enables simultaneous improvement in both hole and electron mobilities. In contrast, the less ordered molecular stacking in PTB7‐Th:Y6 results in reduced mobilities for both types of charge carriers (Figure 2e). Therefore, we conclude that further enhancement of the PCE in low‐donor‐content devices depends primarily on two factors, which are prolonging the *L*_EX+SP_ of the electron‐acceptor, and constructing a morphology with appropriate phase separation and molecular packing order. The former contributes to minimized current density loss by enhancing the contribution from the SP channel, whereas the latter ensures efficient charge transport by optimizing the conventional HJ channel. To realize simultaneously optimized dual‐channel photoelectric conversion, we first replaced the electron‐acceptor with BTP‐eC9, which exhibits a reported *L*_EX+SP_ value of 50 nm [24]. This value is significantly higher than that of Y6, let alone IEICO‐4F. Furthermore, the *∆δ*_Hildebrand_ between PTB7‐Th and BTP‐eC9 was calculated to be 1.6 MPa^1/2^, higher than that between PTB7‐Th and Y6 (Figure S12 and Table S10). This is also verified by the DSC measurement, which yields a *χ*_PTB7‐Th:BTP‐eC9‐DSC_ of 0.75 (Figure S13). In‐depth analysis based on the CF/PTB7‐Th/BTP‐eC9 ternary phase diagram reveals that though the initial composition points remain above the binodal and spinodal lines (Figure 3f, Tables S11 and S12), the worse miscibility between PTB7‐Th and BTP‐eC9 facilitates earlier phase separation, leading to a larger average fibril width (49.1 nm) and higher RMS value (0.92 nm) at a D/A ratio of 1:2 as confirmed by AFM measurements (Figures S18 and S19). Benefit from higher *L*_EX+SP_ and more continuous donor phase, the PTB7‐Th:BTP‐eC9 (1:2) devices exhibit lower current density loss (6.4 mA cm^−2^) and higher FF (64.7%) (Figure S20 and Table S14). We further employed a dual‐additive strategy to achieve a more favorable active layer morphology. In this approach, a solid additive (TCB) was introduced to optimize the SP channel through enhanced ordering of the lamellar stacking, while a liquid additive (DIO) was used to improve the HJ channel by promoting the continuity of the polymer donor phase [24, 54]. The overall film formation time is prolonged from 0.5s to 0.6s upon the addition of dual‐additives or DIO alone (Figure S21). This was strongly supported by the notably increased CL for lamellar stacking (from 5.54 to 6.73 nm) and the enlarged average fibril width (from 79.1 to 99.2 nm) observed at a D/A ratio of 1:4 (Figure 3e; Figure S19 and S22, Table S15). This specific composition yields the maximum product of PCE for the opaque device and AVT for the blend film (Figure S23 and Table S16). Encouragingly, the addition of TCB and DIO led to significant improvements in both the *J*_SC_ (from 20.4 to 23.1 mA cm^−2^) and FF (from 68.3% to 70.2%), resulting in a high PCE of 11.3% (Figure 3g; Figure S24, Table S17). This value even surpasses the PCE achieved at a D/A ratio of 1:2 (10.4%), underscoring the feasibility of enhancing PCE through the optimization of dual‐channel photoelectric conversion. Meanwhile, the AVT of the blend films remains nearly unchanged (65.4% vs. 65.8%), demonstrating great potential of this strategy in fabricating high‐performance ST‐OSCs (Figure S25 and Table S16). It should be noted that the solid and liquid additives do not function independently. The liquid additive also contributes to the optimization of the SP channel, as it can induce an increased CL for lamellar stacking (1.36 nm). However, its lower diffraction peak area (74.6% of the dual‐additive‐containing optimal sample) demonstrates that the solid additive contributes more significantly to SP channel optimization and thus the *J*_SC_ enhancement (Figure S22). While the solid additive induces a decreased average fibril width (52.0 nm) that is detrimental to FF (Figures S18 and S19), this effect underscores that the liquid additive remains the primary contributor to FF improvement. We also ruled out the reduction of bimolecular recombination as the main cause of the *J*_SC_ enhancement, as the improvement (0.2 mA cm^−2^) predicted by the Hecht equation (*J*_SC_/*J*_MAX_ = 2*L*_dri_/*L*(1‐exp(‐*L*/2*L*_dri_)), where *L* is the film thickness) is significantly lower than the actual increase observed (2.7 mA cm^−2^) (Figure S26 and Table S18–S19) [55]. Furthermore, considering that the addition of the dual‐additive leads to an increase in the average fibril width, thereby reducing the D/A heterojunction area available for exciton splitting, we conclude that the *J*_SC_ enhancement is primarily attributed to the optimization of the SP channel, which promotes more charge generation within the acceptor bulk.

### 2.4 Semi‐Transparent Device Performances

**FIGURE 4:**
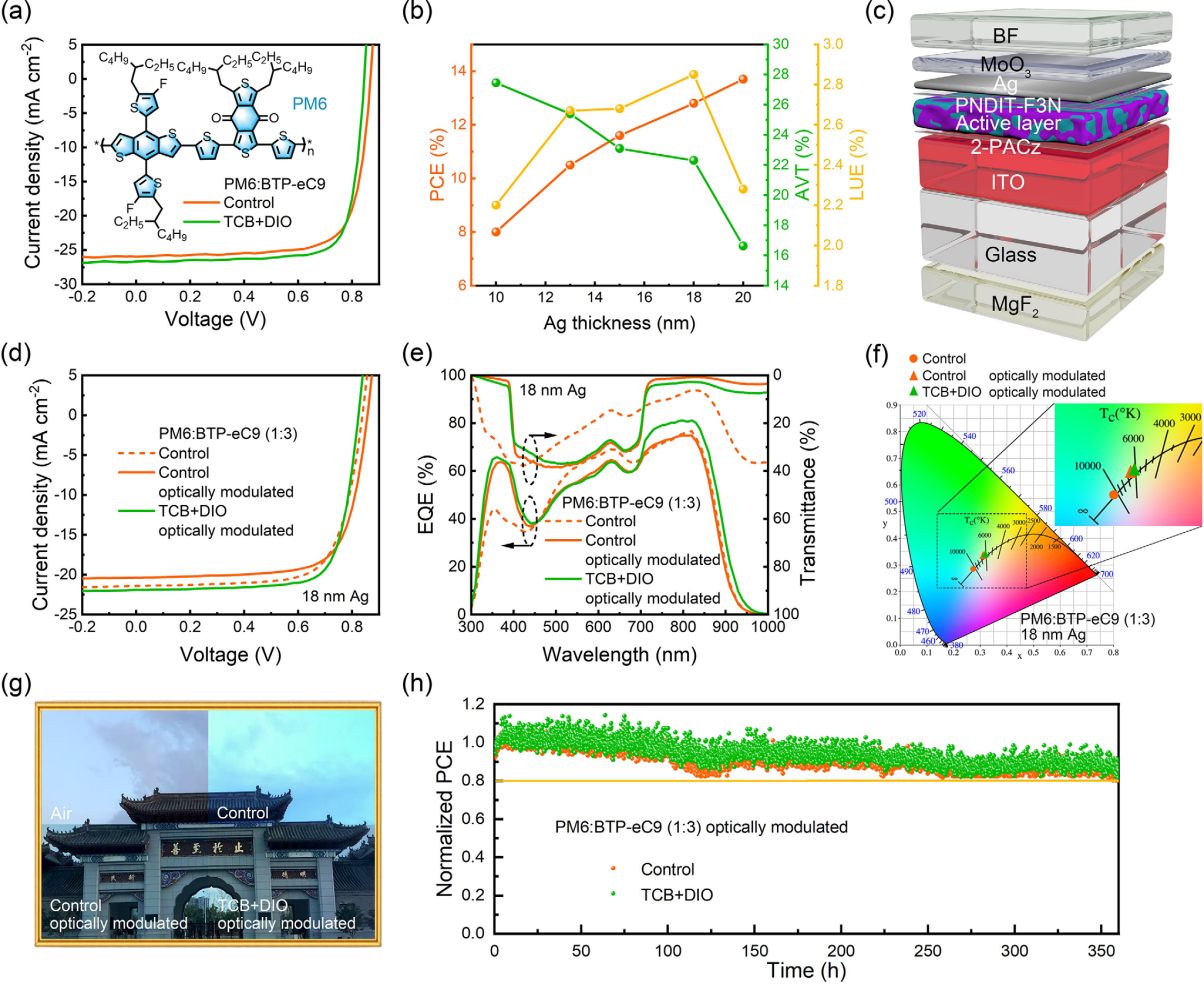
(a) The *J*‐V curves of opaque PM6:BTP‐eC9 (1:3) devices before and after the addition of TCB and DIO. The inset is the molecular structure of PM6. (b) The summarized PCE, AVT, and LUE of the original PM6:BTP‐eC9 (1:3) ST‐OSCs at varies Ag thicknesses (10, 13, 15, 18, and 20 nm). (c) The scheme of device structure of optically modulated ST‐OSCs. (d) The *J*‐V curves, (e) EQE and transmittance spectra, and (f) color coordinates of the original and optically modulated PM6:BTP‐eC9 (1:3) ST‐OSCs before and after the addition of TCB and DIO. (g) Photographs of the south gate of Henan University's Longzihu campus, filtered by air and PM6:BTP‐eC9 ST‐OSCs. (h) MPPT tests of optically modulated PM6:BTP‐eC9 (1:3) ST‐OSCs before and after the addition of TCB and DIO.

We further extended the study to high‐efficiency PM6:BTP‐eC9 devices. At a D/A ratio of 1:3, which is a formulation suitable for ST‐OSC fabrication, the device showed a notable improvement in PCE from 16.9% to 17.4% after the addition of TCB and DIO (Figure 4a and Table S20). This enhancement was accompanied by simultaneous increases in both *J*_SC_ (from 25.5 to 26.3 mA cm^−^^2^) and FF (from 75.8% to 78.6%). A similar trend was observed when the donor polymer was switched from PM6 to D18 (Table S21), underscoring the general applicability of our optimization strategy. These results collectively demonstrate that optimizing dual‐channel photoelectric conversion effectively enhances the performance of low‐donor‐content devices through synergistic improvements in *J*_SC_ and FF. Subsequently, we fabricated ST‐OSCs based on PM6:BTP‐eC9 with a fixed D/A ratio of 1:3 to evaluate the specific contribution of our strategy to LUE improvement, accounting for both PCE and AVT. Initially, the ST‐OSCs were prepared by reducing the thickness of the top Ag cathode (originally 100 nm). A champion LUE of 2.85% was achieved with an 18 nm thick Ag cathode, accompanied by a high PCE of 12.8% but a low AVT of 22.3% (Figure 4b; Table S22). Further improvement in LUE relies primarily on enhancing AVT through strategic optical modulation. To this end, we incorporated three additional layers into the original device structure: a 100 nm thick MgF_2_ film was deposited on the glass substrate to reduce reflection loss; a 35 nm thick MoO_3_ layer was deposited on the Ag cathode to boost full‐spectrum transmittance; and finally, a commercial bandpass filter (BF) was placed on top of the MoO_3_ layer to selectively only transmit visible light (Figure 4c; Figure S27). The results indicate that the optically modulated ST‐OSCs exhibited significantly improved transmittance in the visible region, albeit accompanied by a reduced EQE response (Figure 4d,e). Benefitting from the substantially enhanced AVT of 35.1% and a marginally decreased PCE of 12.6%, the LUE was increased to 4.42%. The introduction of dual additives (TCB and DIO) further elevated the LUE to 4.67%, yielding a high PCE of 13.6% with a slightly reduced AVT of 34.3% (Table S23). Color coordinate analysis revealed that optical modulation shifted the color coordinates from (0.2732, 0.2862) to (0.3191, 0.3425), moving closer to the “white point” D65 (0.3128, 0.3290) (Figure 4f; Table S24). This improved color perception was visually confirmed by photographs taken through the ST‐OSCs (Figure 4g). The photo‐stability of the optically modulated PM6:BTP‐eC9 (1:3) ST‐OSCs was also investigated by implementing maximum power point tracking (MPPT) under the ISOS‐L‐1 protocol. The control device exhibits a T_80_ lifetime of approximately 360 h. Notably, despite the opposing effects of the individual additives, the dual‐additives showed a negligible impact on stability (82.0% retention compared to 79.2% for the control, Figure 4h), which highlights the robustness of our optimizing strategy [56, 57]. Further analysis of the normalized *V*_OC_, *J*_SC_, and FF decay attributes the PCE decay to the combined degradation of all photovoltaic parameters, with the corresponding retention ratios of 93.9%, 97.4%, and 90.0% for the *V*_OC_, *J*_SC_, and FF, respectively (Figure S28). Considering that such results may also be substantially influenced by the stability of interfacial layers (e.g., the SAM layer), we further evaluated device stability using a more robust inverted architecture to focus more directly on the behavior of the active layer itself [58]. It was found that the T_80_ lifetime of the control device reaches 710 h, while the introduction of dual additives unexpectedly slightly increases this value to 900 h. Given the nearly unchanged *V*_OC_ and the degraded but consistent *J*_SC_ values, the slightly higher T_80_ lifetime in the optimized devices can be attributed to a smaller decay in FF (Figure S29). This improvement likely originates from a more ordered molecular stacking, which mitigates conformational changes caused by photo‐isomerization of vinyl groups [59].

## 3 Conclusion

In summary, while the low‐donor‐content strategy is well‐established in state‐of‐the‐art ST‐OSCs for enhanceing transparency, the concomitant efficiency loss remains a major impediment to their advancement. The inherent limitations of both the SP and HJ channels prevent either from individually overcoming this challenge. Through a comparative study of IEICO‐4F and Y6 with differing SP channel contributions, we decoupled the distinct roles of these dual channels on photovoltaic parameters. Specifically, the SP channel allows EXs that far from D/A heterojunction, contributing to charge generation, thus primarily governing the *J*_SC_ decay as donor content decreases. While the FF of low‐donor‐content devices is influenced by the balance between hole transport in the donor phase and electron transport in the acceptor phase, just as controlled by the HJ channel. Consequently, mitigating efficiency loss necessitates the simultaneous optimization of both channels. Herein, leveraging a Y series acceptor BTP‐eC9 known for its superior SP channel contribution, we devised a dual‐additive strategy guided by ternary phase diagram predictions and detailed morphology characterization. The solid additive (TCB) enhances the lamellar stacking order of the acceptor, thereby augmenting the SP channel's role. Meanwhile, the liquid additive (DIO) improves the continuity of the donor phases, thus boosting the HJ channel's contribution. This strategy successfully increased the PCE of PTB7‐Th:BTP‐eC9 (1:4) devices from 9.9% to 11.3%, exceeding that of conventional BHJ counterparts (10.4%), while maintaining a nearly identical AVT after additive treatment (65.4% vs. 65.8%). Furthermore, extending the dual‐additive strategy to PM6:BTP‐eC9 (1:3) ST‐OSCs resulted in an improvement in LUE from 4.42% to 4.67% without compromising photo‐stability, along with a significant PCE increase (13.6% vs. 12.6%) and only a slight AVT reduction (34.3% vs. 35.1%). This work provides a design blueprint for materials and morphology for high‐performance ST‐OSCs, while emphasizing the critical importance of synergistic dual‐channel optimization for guiding the development of next‐generation transparent photovoltaics.

## Author Contributions

Y. T., G. C., and S. Z. conceived and designed the research. Y. L. performed the experiments, including the device fabrication, optical and electrical measurements, DSC, and in situ absorption measurements. S. W. calculated the ternary phase diagram. H. L. and X. L. conducted the GIWAXS characterization. B. S. and H. Y. performed the temperature‐dependent PL tests. Z. Z. and S. K. S. performed the PDS characterization. Y. S. conducted the AFM characterization. Y. L. drafted the manuscript. Y. T., D. D., G. C., and S. Z. reviewed and edited the manuscript. Y. T., G. C., and S. Z. mentored the other authors throughout the investigation. All authors participated in the discussion of the results and reviewed the manuscript.

## Conflicts of Interest

The authors declare no conflicts of interest.

## Supporting information




**Supporting File**: advs74830‐sup‐0001‐SuppMat.docx.

## Data Availability

The data that support the findings of this study are available in the supplementary material of this article.
