# Supplementary material for: Decoupling the Transparency‐Efficiency Trade‐Off in Semi‐Transparent Organic Solar Cells via Optimized Dual‐Channel Photoelectric Conversion
Source: Adv Sci (Weinh). 2026 Mar 14;13(30):e23474. doi: 10.1002/advs.202523474 (PMC13248786; doi:10.1002/advs.202523474)
Supplement: Supplementary file 1 — Supporting File: advs74830‐sup‐0001‐SuppMat.docx. [file ADVS-13-e23474-s001.docx]

Supporting Information

**Decoupling the Transparency-Efficiency Trade-Off in**

**Semi-Transparent Organic Solar Cells via** **Optimized**

**Dual-Channel Photoelectric Conversion**

*Yuyan Li,^1,2^ Shibo Wang,^2^ Heng Liu**,^4^ Bonan Shi,^5^ Zhuoqiong Zhang,^6^ Yabing Tang, ^2*^ Yuting Song, ^2,3^ Shu Kong So,^6^ Dongbin Dang,^1^ Han Yan,^5^ Xinhui Lu, ^4^ Guilong Cai,^1,2,3*^ Suojiang Zhang^1,2,3*^*

Table of content

**Materials** 1

**Instrumentation** 1

**Device fabrication** 3

**Calculating the SCLC mobility** 4

**Calculating the *L*_EX+SP_** 4

**Calculating the energy loss** 6

**Calculating the ternary phase diagram** 8

**Calculating the average visible transmittance** 10

**Calculating the color coordinates** 10

**Calculating the *J*_MAX_** 11

**1D drift-diffusion simulation** 11

**Calculating the EA and IE** 11

**The DSC measurements** 12

**Calculating the bimolecular recombination coefficient** 12

**Calculating the *L*_dr_** 13

**Figure S1.** The extinction coefficient of IEICO-4F and EQE spectra of CuSCN/IEICO-4F devices for different IEICO-4F thicknesses 14

**Figure S2.** The CV measurement of PTB7-Th, IEICO-4F, Y6, and Fc/Fc^+^. 14

**Figure S3.** EQE curves of PTB7-Th:IEICO-4F and PTB7-Th:Y6 devices 14

**Figure S4.** The absorption coefficient of PTB7-Th:IEICO-4F and PTB7-Th:Y6 blend films 15

**Figure S5.** *J*_MAX_ values of PTB7-Th:IEICO-4F and PTB7-Th:Y6 devices 15

**Figure S6.** Transmittance spectra and PCE×AVT values of PTB7-Th:IEICO-4F and PTB7-Th:Y6 active layer 16

**Figure S7.** Hole-only and electron-only charge transport curves of opaque devices 16

**Figure S8.** Drift-diffusion simulations of PTB7-Th:IEICO-4F and PTB7-Th:Y6 devices 17

**Figure S9.** Hole-only charge transport curves of PTB7-Th:Y6 devices 17

**Figure S10.** *E*_g_ of PTB7-Th:IEICO-4F and PTB7-Th:Y6 blend films 17

**Figure S11.** Energy disorders of pure IEICO-4F and Y6 films 18

**Figure S12.** Contact angle measurements of PTB7-Th, IEICO-4F, Y6 and BTP-eC9 18

**Figure S13.** Differential scanning calorimetry (DSC) endothermal curves of IEICO-4F, Y6 and BTP-eC9 with different mass ratio of PTB7-Th to show melting point depression behavior. 19

**Figure S14.** Normalized absorbance of PTB7-Th:IEICO-4F and PTB7-Th:Y6 blend films 19

**Figure S15.** Contour plots of In-situ absorption spectra of PTB7-Th:IEICO-4F and PTB7-Th:Y6 blend films 20

**Figure S16.** The average fibril width of PTB7-Th:IEICO-4F and PTB7-Th:Y6 blend films 20

**Figure S17.** Two-dimensional GIWAXS scattering patterns of PTB7-Th:IEICO-4F and PTB7-Th:Y6 blend films 21

**Figure S18.** AFM images of PTB7-Th:BTP-eC9 blend films 21

**Figure S19.** The average fibril width of PTB7-Th:BTP-eC9 blend films 22

**Figure S20.** The absorption coefficient and *J*_MAX_ value of PTB7-Th:BTP-eC9 device 22

**Figure S21.** Contour plots of In-situ absorption spectra of PTB7-Th:BTP-eC9 blend films 23

**Figure S22.** Two-dimensional GIWAXS scattering patterns of PTB7-Th:BTP-eC9 blend films 23

**Figure S23.** EQE curves, transmittance spectra and PCE×AVT values of PTB7-Th:BTP-eC9 active layer 24

**Figure S24.** EQE curves of PTB7-Th:BTP-eC9 devices 24

**Figure S25.** Transmittance spectra of PTB7-Th:BTP-eC9 (1:4) blend films with and without additives. 25

**Figure S26.** The absorption coefficient of PTB7-Th:BTP-eC9 device. 25

**Figure S27.** Transmittance Spectrum of the bandpass filter (BF). 26

**Figure S28.** Evolution of normalized *V*_OC_, *J*_SC_ and FF decay of optically modulated PM6:BTP-eC9 (1:3) ST-OSCs 26

**Figure S29.** MPPT measurements of the unencapsulated optically modulated PM6:BTP-eC9 (1:3) inverted architecture. 26

**Table S1.** Film thicknesses, hole, and electron mobilities of IEICO-4F and Y6 27

**Table S2.** Photovoltaic performance of PTB7-Th:IEICO-4F and PTB7-Th:Y6 devices 27

**Table S3.** *J*_MAX_, *J*_SC_ and the corresponding current density loss of PTB7-Th:IEICO-4F and PTB7-Th:Y6 devices 28

**Table S4.** PCE of opaque devices, AVT of blend films,PCE×AVT of PTB7-Th:IEICO-4F and PTB7-Th:Y6 28

**Table S5.** Film thicknesses, hole and electron mobilities of PTB7-Th:IEICO-4F and PTB7-Th:Y6 28

**Table S6.** Parameters of PTB7-Th:IEICO-4F and PTB7-Th:Y6 devices via drift-diffusion simulations 29

**Table S7.** Photovoltaic performance of PTB7-Th:IEICO-4F and PTB7-Th:Y6 devices via drift-diffusion simulations 29

**Table S8.** Film thicknesses, hole mobilities of PTB7-Th:Y6 29

**Table S9.** Summary of calculated energy loss parameters of PTB7-Th:IEICO-4F and PTB7-Th:Y6 devices. 29

**Table S10.** Parameters for calculation of the Flory-Huggins interaction parameters (*χ*) and ternary phase diagram 30

**Table S11.** Flory-Huggins interaction parameters calculated and the degree of polymerization (*N*_i_) for PTB7-Th, IEICO-4F, Y6, and BTP-eC9. 30

**Table S12.** Initial component points (*φ*_CF_, *φ*_PTB7-Th_, *φ*_acceptor_) of PTB7-Th:IEICO-4F, PTB7-Th:Y6 and PTB7-Th:BTP-eC9 devices 30

**Table S13.** The fitted peak location, d-spacing, full width at half maximum (FWHM), coherence length, and peak area from GIWAXS patterns of PTB7-Th:IEICO-4F and PTB7-Th:Y6 blend films 31

**Table S14.** Photovoltaic performance of PTB7-Th:BTP-eC9 devices 31

**Table S15.** The fitted peak location, d-spacing, FWHM, coherence length and peak area from GIWAXS patterns of PTB7-Th:BTP-eC9 blend films 32

**Table S16.** PCE of opaque devices, AVT of blend films, PCE×AVT of PTB7-Th:BTP-eC9 32

**Table S17.** Photovoltaic performance of PTB7-Th:BTP-eC9 devices 33

**Table S18.** Film thicknesses, hole and electron mobilities of PTB7-Th:BTP-eC9 device 33

**Table S19.** Parameters for calculating the carrier drift length (*L*_dr_) 33

**Table S20.** Photovoltaic performance of PM6:BTP-eC9 devices at a D/A ratio of 1:3 with and without the addition of TCB and DIO 34

**Table S21.** Photovoltaic performance of D18:BTP-eC9 devices at a D/A ratio of 1:3 with and without the addition of TCB and DIO 34

**Table S22.** Photovoltaic performance of PM6:BTP-eC9 (1:3) ST-OSCs with various Ag thicknesses. 35

**Table S23.** Photovoltaic performance of PM6:BTP-eC9 (1:3) ST-OSCs devices. 35

**Table S24.** Color coordinates of PM6:BTP-eC9 (1:3) ST-OSCs 35

**References.** 36

**Experimental Section**

**Materials.** PM6 (poly[(2,6-(4,8-bis(5-(2-ethylhexyl-3-fluoro)thiophen-2-yl)-benzo[1,2-b:4,5-b′]dithiophene))-alt-(5,5-(1′,3′-di-2-thienyl-5′,7′-bis(2-ethylhexyl)benzo[1′,2′-c:4′,5′-c′]dithiophene-4,8-dione)]), D18 (poly[(2,6-(4,8-bis(5-(2-ethylhexyl-3-fluoro)thiophen-2-yl)-benzo[1,2-b:4,5-b′]dithiophene))-alt-(2-butyloctyl) thiophen-2-yl)-8-(4-(2-butyloctyl)-5-methylthiophen-2-yl)dithieno[3′,2′:3,4;2′′,3′′:5,6]benzo[1,2-c][1,2,5]thiadiazole)]), BTP-eC9 (2,2′-[[12,13-Bis(2-butyloctyl)-12,13-dihydro-3,9-dinonylbisthieno[2′′,3′′:4′,5′]thieno[2′,3′:4,5]pyrrolo[3,2-e:2′,3′-g][2,1,3]benzothiadiazole-2,10-diyl]bis[methylidyne(5,6-chloro-3-oxo-1H-indene-2,1(3H)-diylidene)]]bis[propanedinitrile]), and IEICO-4F (2,2′-((2Z,2′Z)-(((4,4,9,9-tetrakis(4-hexylphenyl)-4,9-dihydro-sindaceno[1,2-b:5,6-b′]dithiophene-2,7-diyl)bis(4-((2-ethylhexyl)oxy)thiophene-5,2-diyl))bis(methanylylidene))bis(5,6-difluoro-3-oxo-2,3-dihydro-1H-indene-2,1- diylidene))dimalononitrile) were purchased from Solarmer Materials Inc. PTB7-Th (poly[4,8-bis(5-(2-ethylhexyl)-thiophene-2-yl)benzo[1,2-b;4,5-b′]dithiophene-2,6-diyl-alt-(4-(2-ethylhexyl)-3-fluorothieno[3,4b]thiophene-)-2-carboxylate-2-6-diyl]]) and Y6 (2,2′-((2Z,2′Z)-((12,13-bis(2-ethylhexyl)-3,9-diundecyl-12,13-dihydro-[1,2,5]thiadiazolo[3,4-e]thieno[2′′,3′′:4′,5′]thieno[2′,3′:4,5]pyrrolo[3,2-g]thieno[2′,3′:4,5]thieno[3,2-b]indole-2,10-diyl)bis(methanylylidene))bis(5,6-difluoro-3-oxo-2,3-dihydro-1H-indene-2,1-diylidene))dimalononitrile) were purchased from Nanjing Zhiyan Technology Co., Ltd. TCB and DIO were purchased from Tokyo Chemical Industry (TCI). PNDIT-F3N was purchased from eFlexPV Inc. 2-PACz was purchased from Suzhou Liwei New Material Technology Co. Ltd. CF, methanol and ethanol were purchased from Sigma-Aldrich. Diethyl sulfide (DES) was purchased from J&K Scientific‌. CuSCN, MgF_2_ were purchased from Macklin. All chemicals were used as received without further purification.

**Instrumentation.** The *J*-*V* curves were performed in the N_2_-filled glovebox under AM 1.5G (100 mW cm^−2^) using an AAA solar simulator (SS-X50, Enlitech) and a programmable sourcemeter (B2901BL, Enlitech), calibrated by the certified standard silicon solar cell (SRC-2020, Enlitech). The EQE curves were recorded through the solar cell spectral response measurement system (QE-R, Enlitech) with calibrated light intensity by a standard Si photovoltaic cell. Transient photocurrent (TPC) was detected by an integrated testing system (TPCV, Enlitech). The EQE_EL_ measurement was performed on REPS-PRO (Enlitech). The absorbance was obtained on a Shimadzu UV-3600 Plus Spectrophotometer. The TM-AFM was measured by Cypher ES (Oxford Instruments). The film thickness was measured by Bruker Dektak XT. Contact angle measurement was performed on Biolin Theta Lite. Temperature-dependent PL spectra were recorded by FLS980 spectrometer (Edinburgh Instruments, EI). The temperature was controlled by the OXFORD attachment. GIWAXS was conducted on active layer films at the Brockhouse X-ray Diffraction and Scattering Sector Low Energy Wiggler (BXDS-WLE) beamline of the Chinese University of Hong Kong using a photon energy of 15.12 keV (*λ* = 0.82 Å). A Rayonix MX300 detector 328.04 mm away from the sample was used for collecting patterns. The PDS measurements were performed with a 1 kW Xe arc lamp and a 1/4 m grating monochromator (Oriel) as the tunable light source. The pump beam was modulated at 13 Hz by using a mechanical chopper before irradiating the sample. Perfluorohexane was used as the deflection fluid. A Uniphase He–Ne laser was directed parallel to the sample surface as the probe laser. A quadrant cell (United Detector Technology) was used as the position sensor for monitoring the photothermal deflection signal of the probe beam. The output of the detector was fed into a lock-in amplifier (Stanford Research, Model SR830) for phase-sensitive measurements. All PDS spectra were normalized to the incident power of the pump beam. Samples were prepared on quartz substrates using identical blend solutions as those used in devices. The MPPT (maximum power point tracking) measurements were conducted using a multi-channel solar cell stability test system (MSCLT-1, Wuhan 91pvk Solar Technology Co., Ltd., China). The semitransparent devices were fabricated following the same procedure described earlier. The inverted architecture had a structure of ITO/ZnO/active layer/MoO_3_/Ag. The in-situ absorption measurement was conducted on a lab-assembled setup integrated with a USB2000+ spectrometer. The same blend solution as used in device fabrication was spin-coated onto a glass substrate pre-deposited with a 200nm thick Ag electrode layer. The spectral signals were acquired in reflection mode during the in-situ measurement.

**Device fabrication.** The opaque BHJ devices were fabricated in a traditional device structure of ITO/2-PACz/active layer/PNDIT-F3N/Ag. Patterned ITO substrates were first cleaned by successive ultrasonication in glass cleaning solution, deionized water, acetone, and isopropanol. Prior to the deposition of the 2-PACz layer, the substrates were subjected to ultraviolet ozone treatment for 20 min, followed by plasma cleaning for 5 min. 2-PACz solution (0.2 mg/mL in ethanol) was spin-coated at 3000 rpm for 30 s and then thermally annealed at 100 °C for 10 min. Then, PTB7-Th:IEICO-4F, PTB7-Th:Y6, and PTB7-Th:BTP-eC9 blend solutions were prepared in CF at various D/A ratios (1:2, 1:3, 1:4, 1:5, and 1:7), with a fixed acceptor concentration of 15 mg/mL for IEICO-4F and 9.6 mg/ml for Y6 and BTP-eC9, respectively. Similarly, PM6:BTP-eC9 and D18:BTP-eC9 blends were prepared in CF at a D/A ratio of 1:3 and a fixed BTP-eC9 concentration of 8.7 mg/ml. Dual additives (TCB and DIO) were introduced during solution preparation. After stirring on a 100 °C hotplate for over 4 h, the blend solutions were spin-coated onto the 2-PACz layer to form the active layers. Subsequently, a PNDIT-F3N layer was deposited by spin-coating (0.5 mg/mL in methanol with 0.5 vol% acetic acid, 2000 rpm, 30 s) onto the active layer. Finally, 100 nm Ag was deposited at a vacuum level of < 1×10^-4^ Pa. Typical devices area (0.0216 cm^2^) was defined by a metal mask.

The optically modulated ST-OSCs were fabricated in the aforementioned traditional device structure, incorporating a thin Ag electrode (1 Å s^-1^) topped with 35 nm MoO_3_ (0.1 Å s^-1^) to boost transmittance, and a 100 nm MgF_2_ anti-reflection layer on the glass side, all deposited under a vacuum of < 1×10^-4^ Pa.

For the fabrication of bilayer devices to calculate *L*_EX+SP_, the patterned ITO substrates were first cleaned following the opaque BHJ devices. CuSCN (40 mg/ml) was dissolved in DES at 60 °C for 1 h and then filtered. Then the CuSCN solution was spin-coated on ITO substrates at 1500 rpm for 30 s, followed by annealing of the device at 105 °C for 10 min to obtain thin films with thickness of ca. 60 nm. IEICO-4F was dissolved in CF with different concentrations (1-12 mg/ml) and was spin-coated on CuSCN layer at different speeds for 30 s to obtain film thicknesses ranging from 25 nm to 120 nm. Next, a PNDIT-F3N layer was deposited by spin-coating (0.5 mg/mL in methanol with 0.5 vol% acetic acid, 2000 rpm, 30 s) onto IEICO-4F. Finally, 100 nm Ag was deposited at a vacuum level of < 1×10^-4^ Pa.

**Calculating the SCLC mobility:**

The SCLC mobility (*μ*) was measured with the hole-only device structure of ITO/PEDOT:PSS (Heraeus Clevios PVP Al 4083)/active layer/MoO_3_/Ag and electron-only device structure of ITO/ZnO/active layer/PNDIT-F3N/Ag. ZnO was deposited by spin-coating a ZnO precursor solution (zinc acetate dihydrate, dissolved in 2-methoxyethanol with ethanolamine) on the ITO substrates at a speed of 4500 rpm for 30 s, followed by thermal annealing at 200 °C for 30 min. PEDOT:PSS was spin-coated on the ITO substrates at a speed of 5500 rpm for 30 s, followed by thermal annealing at 150 °C for 10 min. The values of SCLC mobility were obtained by fitting the current density-voltage curves according to

| $J=\frac{9\varepsilon_{0}\varepsilon_{r}\mu V^{2}}{8L^{3}}$ | (S1) |
| --- | --- |

Here, *ε*_0_ is the vacuum dielectric constant (8.85×10^-12^ F^-1^m^-1^), *ε*_r_ is the relative dielectric constant (assumed to be 3 here), and *L* is the film thickness.

**Calculating the *L*_EX+SP_:**^[1-5]^

According to previous reports, the EQE spectra of the bilayer devices with a series acceptor film thicknesses can be modeled with the well-known one-dimensional exciton diffusion equation:

| $\frac{\partial n(x,t)}{\partial t}=D\frac{\partial^{2}n(x,t)}{\partial x^{2}}+G\left( x,t \right)-k_{\mathrm{PL}}n\left( x,t \right)-k_{\mathrm{FRET}}n\left( x,t \right)-\gamma n\left( x,t \right)^{2}$ | (S2) |
| --- | --- |

where *G*(*x*, *t*) is the time-dependent exciton generation profile at position *x* given by transfer-matrix modeling, *k*_PL_ is the radiative decay rate without quencher sites, *γ* is an exciton-exciton annihilation rate constant, and *k*_FRET_ denotes the rate of Förster resonance energy transfer (FRET) in the presence of a neighboring material. It is worth noting that FRET between CuSCN and IEICO-4F is negligible owing to the small overlap of CuSCN’s absorption with IEICO-4F’s emission, so *k*_FRET_ is zero. Due to the small light intensity during EQE measurements, exciton-exciton annihilation rate constant (*γ*) is also regarded as zero. Hence, Eq. (S2) can be simplified under steady-state conditions with

| $k_{\mathrm{PL}}=\frac{D}{L_{EX+SP}^{2}}$ | (S3) |
| --- | --- |

as

| $\left( \frac{\partial^{2}}{\partial x^{2}}-\frac{1}{L_{EX+SP}^{2}} \right)n\left( x \right)=-\frac{G(x)}{D}$ | (S4) |
| --- | --- |

The general solution can be written as:

| $Dn\left( x \right)=\left[ k_{1}-\frac{L_{EX+SP}}{2}\int_{x_{0}}^{x} e^{-\frac{\hat{x}}{L_{EX+SP}}}G\left( \hat{x} \right)d\hat{x} \right]e^{\frac{x}{L_{EX+SP}}}+\left[ k_{2}+\frac{L_{EX+SP}}{2}\int_{x_{0}}^{x} e^{\frac{\hat{x}}{L_{EX+SP}}}G\left( \hat{x} \right)d\hat{x} \right]e^{-\frac{x}{L_{EX+SP}}}$ | (S5) |
| --- | --- |

where *k*_1_ and *k*_2_ are constants and can be solved with boundary conditions of complete exciton quenching at IEICO-4F/CuSCN interface (*n*=0) and complete exciton reflection at IEICO-4F/PNDIT-F3N interface (∂*n*/∂*x*=0). So they are given as:

| $k_{1}={-k_{2}e}^{-\frac{2x_{0}}{L_{EX+SP}}}$ | (S6) | |
| --- | --- | --- |
| $k_{2}=\frac{-\frac{L_{EX+SP}}{2}\int_{x_{0}}^{0} e^{-\frac{\hat{x}}{L_{EX+SP}}}G\left( \hat{x} \right)d\hat{x}-\frac{L_{EX+SP}}{2}\int_{x_{0}}^{0} e^{\frac{\hat{x}}{L_{EX+SP}}}G\left( \hat{x} \right)d\hat{x}}{e^{-\frac{2x_{0}}{L_{EX+SP}}}+1}$ | (S7) |  |

The EQE can then be calculated considering that the photocurrent is only due to the exciton dissociation at the CuSCN/IEICO-4F interface:

| $\mathrm{EQE}=\frac{J_{photo}}{J_{inc}}=\frac{qn_{c}D}{J_{inc}}\left. \frac{\partial n(x)}{\partial x} \right\vert_{interface}$ | (S8) |
| --- | --- |

where *J*_photo_ and *J*_inc_ are the generated photocurrent density and the incident light current density. And the shape of normalized EQE vs. thickness data is only determined by *D*∂*n*(x)/∂*x*, which is given by

| $D\frac{\partial n\left( x \right)}{\partial x}\left( x=x_{0} \right)=\frac{1}{L_{EX+SP}}e^{\frac{x}{L_{EX+SP}}}k_{1}-\frac{1}{L_{EX+SP}}e^{-\frac{x}{L_{EX+SP}}}k_{2}$ | (S9) |
| --- | --- |

**Calculating the energy loss:**^[6,7]^

Based on detailed balance and reciprocity, energy loss can be expressed as the sum of three components, as shown below:

| ${q\Delta V}_{\mathrm{loss}}=\Delta E_{1}+\Delta E_{2}+\Delta E_{3}$ | |  |  |
| --- | --- | --- | --- |
| $=\left( E_{g}-qV_{\mathrm{OC}}^{\mathrm{SQ}} \right)+\left( qV_{\mathrm{OC}}^{\mathrm{SQ}}-qV_{\mathrm{OC}}^{\mathrm{rad}} \right)+(qV_{\mathrm{OC}}^{\mathrm{rad}}-qV_{\mathrm{OC}})$ | |  |  |
| $=\left( E_{g}-qV_{\mathrm{OC}}^{\mathrm{SQ}} \right)+qV_{\mathrm{OC}}^{rad,below gap}+qV_{\mathrm{OC}}^{\mathrm{nrad}}$ | (S10) | | |

where *∆V*_loss_ is the total voltage loss, *E*_g_ is band gap, *V*_OC_^SQ^ is the maximum voltage deduced by the Shockley-Queisser, *V*_OC_^rad^ is the open-circuit voltage when there is only radiative recombination, *∆V*_OC_^rad, below gap^ is the voltage loss of radiative recombination from the absorption below the bandgap, *∆V*_OC_^nrad^ is the voltage loss of non-radiative recombination.

The intersect of the extrapolated baseline and absorption edge defines the *λ*_on-set_, and then the *E*_g_ can be calculated by *E*_g_ = 1240/*λ*_on-set_.

In order to calculate the energy loss parameters, we should firstly understand where the losses are from. The *V*_OC_ of any type of solar cells is determined by the ratio between short circuit current (*J*_SC_) and dark saturation current (*J*_0_), following this expression:

| $V_{oc}=\frac{k_{B}T}{q}\ln(\frac{J_{\mathrm{SC}}}{J_{0}}+1)$ | (S11) |
| --- | --- |

where *k*_B_ is the boltzmann constant, *T* is the temperature, and *q* is the elementary charge. The expression for *J*_SC_ and *J*_0_ are given by:

| $J_{\mathrm{SC}}=q\cdot\int_{0}^{\infty} \mathrm{EQE}_{\mathrm{pv}}(E)\cdot\varphi_{AM1.5}\left( E \right)dE$ | (S12) |
| --- | --- |
| $J_{0}=\frac{q}{{EQE}_{\mathrm{EL}}}\cdot\int_{0}^{\infty} \mathrm{EQE}_{\mathrm{pv}}(E)\cdot\varphi_{\mathrm{bb}}\left( E \right)dE$ | (S13) |

The expression for *J*_0_ is the Rau’s reciprocity relation, where EQE_EL_ is radiative quantum efficiency of the solar cell when charge carriers are injected into the device in dark, *φ*_AM1.5_ is the AM1.5 standard solar spectrum and *φ*_bb_ is the black body spectrum. When all the recombination is radiative (i.e. EQE_EL_ = 1), *J*_0_ is minimized, and *V*_oc_ is maximized:

| $J_{0}^{rad}=q\cdot\int_{0}^{\infty} \mathrm{EQE}_{\mathrm{pv}}(E)\cdot\varphi_{\mathrm{bb}}\left( E \right)dE$ | (S14) |
| --- | --- |
| $V_{\mathrm{OC}}^{\mathrm{rad}}=\frac{k_{B}T}{q}\ln(\frac{J_{\mathrm{SC}}}{J_{0}^{rad}}+1)=\frac{k_{B}T}{q}\ln(\frac{q\cdot\int_{0}^{\infty} \mathrm{EQE}_{\mathrm{pv}}(E)\cdot\varphi_{AM1.5}\left( E \right)dE}{q\cdot\int_{0}^{\infty} \mathrm{EQE}_{\mathrm{pv}}(E)\cdot\varphi_{\mathrm{bb}}\left( E \right)dE}+1)$ | (S15) |

In the Shockley-Queisser theory, the general quantum efficiency EQE_pv_^SQ^(E) can be defined as follow:

| $\left\{ \begin{aligned} \mathrm{EQE}_{\mathrm{PV}}^{\mathrm{SQ}}\left( E \right)=1,E>E_{g} \\ \mathrm{EQE}_{\mathrm{PV}}^{\mathrm{SQ}}\left( E \right)=0,E<E_{g} \end{aligned} \right.$ | (S16) |
| --- | --- |

Substituting general quantum efficiency EQE_pv_^SQ^(E) (equation S16) in equations S12 and S14, then we can get the short circuit current and dark saturation current in the SQ limit:

| $J_{\mathrm{SC}}^{\mathrm{SQ}}= q\cdot\int_{E_{g}}^{\infty} \varphi_{AM1.5}\left( E \right)dE$ | (S17) |
| --- | --- |
| $J_{0}^{\mathrm{SQ}}= q\cdot\int_{E_{g}}^{\infty} \varphi_{\mathrm{bb}}\left( E \right)dE$ | (S18) |

In the same way, we can calculate the value of the SQ open-circuit voltage limit, *V*_OC_^SQ^ according to equation S15:

| $V_{\mathrm{OC}}^{\mathrm{SQ}}=\frac{k_{B}T}{q}\ln(\frac{J_{\mathrm{SC}}^{\mathrm{SQ}}}{J_{0}^{\mathrm{SQ}}}+1)=\frac{k_{B}T}{q}\ln(\frac{q\cdot\int_{E_{g}}^{\infty} \varphi_{AM1.5}\left( E \right)dE}{q\cdot\int_{E_{g}}^{\infty} \varphi_{bb}\left( E \right)dE}+1)$ | (S19) |
| --- | --- |

The difference between *V*_OC_^SQ^ and *V*_OC_^rad^ is due to that in the SQ theory, the band edge of the absorber is totally abrupt when calculating *V*_OC_^rad^, the band gap will be smeared out for the existence of charge transfer state absorption.

Therefore, we can deduce the voltage loss of radiative recombination below the gap

| $V_{\mathrm{OC}}^{rad,below gap}=V_{\mathrm{OC}}^{\mathrm{SQ}}-V_{\mathrm{OC}}^{\mathrm{rad}}$ | (S20) |
| --- | --- |

The voltage loss due to non-radiative recombination, *V*_OC_^rad^, can be rewritten as

| $V_{\mathrm{OC}}^{\mathrm{nrad}}=V_{\mathrm{OC}}^{\mathrm{rad}}-V_{\mathrm{OC}}=-k_{B}T\ln\mathrm{EQE}_{\mathrm{EL}}$ | (S21) |
| --- | --- |

From which we can calculate the *V*oc value. Based on the previous discussions, we are now able to summarize the energy loss from the *E*_g_ to the *qV*_OC_ for any type of solar cells. We can get these three terms of energy losses based on related experiments and calculations.

**Calculating the ternary phase diagram:** ^[8-10]^

According to the lattice-based hypothesis for molecular chain fluids, a simple model to describe the film formation in the ternary system was given by Altena. The free energy of the mixing is given by the equation:

| $\frac{\Delta G_{M}}{RT}=n_{1}ln\varphi_{1}+n_{2}ln\varphi_{2}+n_{3}ln\varphi_{3}+ꭓ_{12}n_{1}\varphi_{2}+ꭓ_{13}n_{1}\varphi_{3}+ꭓ_{23}N_{2}n_{2}\varphi_{3}$ | (S22) |
| --- | --- |

where the subscript M indicates the mixing process, *R* is the universal gas constant, *n*_i_ is the molar number, and *φ*_i_ is volume fraction of component i ∈ {1, 2, 3}|{1 = CF, 2 = PTB7-Th, 3 = acceptor}. *N*_i_ is the number of lattice occupied by one molecular of component i which is calculated by the molecular weight and segment molar ratio. Hence, *N*_1_=1, and

| $N_{2}=\frac{molecular weight of polymer}{molecular weight of segment}\times\frac{molar volume of segment}{molar volume of CF}$ | (S23) |
| --- | --- |
| $N_{3}=\frac{molar volume of the acceptor}{molar volume of CF}$ | (S24) |

*χ*_ij_ represents the Flory-Huggins interaction parameter between component *i* and component *j*. The Flory-Huggins interaction parameters are calculated by Hildebrand solubility parameters for materials with similar polarity according to the expression:

| $ꭓ_{\mathrm{ij}}=\frac{v_{1}}{RT}{(\delta_{i}-\delta_{j})}^{2}+0.34$ | (S25) |
| --- | --- |

where *v*_1_ represents the molar volume of CF, 0.34 is the empirical value for entropy contribution, and 𝛿_i_ is the solubility parameter of component *i*, which is expressed as:

| $\delta_{i}=K\sqrt{\gamma_{i}}$ | (S26) |
| --- | --- |

where *γ*_i_ is the surface energy calculated based on the contact angle via Neumann’s method, and K is a proportionality constant simply given by 116×10^3^𝑚^-1/2^.

The binodal line demarcates the boundary between the single-phase and metastable regions. It defines the onset of phase separation. Using the definition of the chemical potentials:

| $\Delta\mu_{i}=\frac{\partial\Delta G_{M}}{\partial n_{i}}$ | (S27) |
| --- | --- |

The binodal line is calculated according to the following equations:

| $\frac{\Delta\mu_{1}}{RT}=\ln\varphi_{1}+\left( 1-\varphi_{1} \right)-\frac{\varphi_{2}}{N_{2}}-\frac{\varphi_{3}}{N_{3}}+\left( {ꭓ_{12}\varphi}_{2}+{ꭓ_{13}\varphi}_{3} \right)\left( \varphi_{2}+\varphi_{3} \right)-{ꭓ_{23}\varphi}_{2}\varphi_{3}$ | (S28) |
| --- | --- |
| $\frac{\Delta\mu_{2}}{RT}=\ln\varphi_{2}+\left( 1-\varphi_{2} \right)-\varphi_{1}N_{2}-\frac{\varphi_{3}N_{2}}{N_{3}}+\left( {ꭓ_{12}\varphi}_{1}N_{2}+{ꭓ_{23}\varphi}_{3}N_{2} \right)\left( \varphi_{1}+\varphi_{3} \right)-{ꭓ_{13}\varphi}_{1}\varphi_{3}N_{2}$ | (S29) |
| $\frac{\Delta\mu_{3}}{RT}=\ln\varphi_{3}+\left( 1-\varphi_{3} \right)-\varphi_{1}N_{3}-\frac{\varphi_{2}N_{3}}{N_{2}}+\left( {ꭓ_{13}\varphi}_{1}N_{3}+{ꭓ_{23}\varphi}_{2}N_{3} \right)\left( \varphi_{1}+\varphi_{2} \right)-{ꭓ_{12}\varphi}_{1}\varphi_{2}N_{3}$ | (S30) |
| $\Delta\mu_{i,A}=\Delta\mu_{i,B}$ | (S31) |
| $\varphi_{1,A}+\varphi_{2,A}+\varphi_{3,A}=\varphi_{1,B}+\varphi_{2,B}+\varphi_{3,B}=1$ | (S32) |

The tie lines connect the compositions with equal chemical potentials. The region inside the spinodal line is completely unstable, it can be evaluated from the relation:

| $\frac{\partial^{2}(\Delta G_{M})}{\partial\varphi_{2}^{2}}\frac{\partial^{2}\left( \Delta G_{M} \right)}{\partial\varphi_{3}^{2}}-\frac{\partial^{2}(\Delta G_{M})}{\partial\varphi_{2}\partial\varphi_{3}}\frac{\partial^{2}\left( \Delta G_{M} \right)}{\partial\varphi_{2}\partial\varphi_{3}}=0$ | (S33) |
| --- | --- |

The Gibbs free energy can be transformed to the following equation:

| $\frac{\Delta G_{M}}{RTn_{tot}}=\varphi_{1}\ln\varphi_{1}+\frac{\varphi_{2}\ln\varphi_{2}}{N_{2}}+\frac{\varphi_{3}\ln\varphi_{3}}{N_{3}}+{ꭓ_{12}\varphi}_{1}\varphi_{2}+{ꭓ_{13}\varphi}_{1}\varphi_{3}+{ꭓ_{23}\varphi}_{2}\varphi_{3}$ | (S34) |
| --- | --- |

The second order derivatives of the Gibbs free energy are as follows:

| $\frac{\partial^{2}(\frac{\Delta G_{M}}{RTn_{\mathrm{tot}}})}{\partial\varphi_{2}^{2}}=\frac{1}{\varphi_{1}}+\frac{1}{\varphi_{2}N_{2}}-2ꭓ_{12}$ | (S35) |
| --- | --- |
| $\frac{\partial^{2}(\frac{\Delta G_{M}}{RTn_{\mathrm{tot}}})}{\partial\varphi_{3}^{2}}=\frac{1}{\varphi_{1}}+\frac{1}{\varphi_{3}N_{3}}-2ꭓ_{13}$ | (S36) |
| $\frac{\partial^{2}(\frac{\Delta G_{M}}{RTn_{\mathrm{tot}}})}{\partial\varphi_{2}\partial\varphi_{3}}=\frac{1}{\varphi_{1}}+ꭓ_{23}-ꭓ_{13}-ꭓ_{12}$ | (S37) |

**Calculating the average visible transmittance**:^[11]^

The AVT value was calculated according to the average value of transmittance of semi-transparent devices based on photonic response of the human eye. The wavelength range is usually adopted by 370-740 nm, and the specific calculation formula is

| $AVT=\frac{\int T(\lambda)F(\lambda)E(\lambda)d\lambda}{\int F(\lambda)E(\lambda)d\lambda}$ | (S38) |
| --- | --- |

where *T*(*λ*) is the transmission spectra of semitransparent devices, *F*(*λ*) is photon flux under AM 1.5G light illumination conditions and *E*(*λ*) is the photonic response of the human eye.

**Calculating the color coordinates:**^[11]^

The color coordinates (*x*, *y*) of semitransparent devices were calculated according to the transmission spectra based on chromaticity diagram of the CIE 1931xy. The color coordinates were calculated by the formulas

| $\left\{ \begin{aligned} X=\int\varphi\left( \lambda\right)*\bar{x}\left( \lambda\right)*d\lambda\\ Y=\int\varphi\left( \lambda\right)*\bar{y}\left( \lambda\right)*d\lambda\\ Z=\int\varphi\left( \lambda\right)*\bar{z}\left( \lambda\right)*d\lambda\end{aligned} \right.$ | (S39) |
| --- | --- |
| $\left\{ \begin{aligned} x=\frac{X}{X+Y+Z} \\ y=\frac{Y}{X+Y+Z} \\ z=\frac{Z}{X+Y+Z} \end{aligned} \right.$ | (S40) |

where X, Y, Z are tristimulus values, *φ*(*λ*) is the spectral power distribution (SPD) of the transmission spectra of semitransparent devices and (λ), (λ), (λ) are color-matching functions.

**Calculating the *J*_MAX_:**^[12-14]^

The simulated exciton generation profiles and *J*_MAX_ values were obtained by the transfer-matrix modeling. The calculation divided the device structure into multiple thin sublayers, and each sublayer’s extinction coefficient *k* can be calculated from the absorption spectrum according to A(*λ*)=lg(*I*_0_/*I*)=-lg(e^-4π^*^k^*^d/^*^λ^*). The refractive index *n* is seted as 2 for organic layers, since its value does not largely impact the generated spectrum comparing with using the more accurate values. Then we can calculate the electric field *E*(x) at an arbitrary position *x* according to previous reports, and the exciton generation profiles *G*(*x*) at an arbitrary position *x* can be obtained on the basis of the electric field by:

| $G\left( x \right)=\eta\int_{\lambda_{1}}^{\lambda_{2}} \frac{2\pi\varepsilon_{0}kn}{h}\left\vert E\left( x \right) \right\vert^{2}d\lambda$ | (S41) |
| --- | --- |

where *η* is the ratio of generated excitons and absorbed photos, *h* is the Planck constant, *λ*_2_ and *λ*_1_ are the upper and lower limits of the integrated wavelength range, and it is 300 nm and 1000 nm in this work. Integrating G(x) through the active layer gives the value of *J*_max_.

**1D drift-diffusion simulation:**^[15-16]^

The 1D drift-diffusion simulations were performed using the opensource package SIMsalabim. To simplify the simulation, we assumed an ohmic contact at the interface between the active layer and electrodes, and the influence of bulk and interface traps were ignored as well.

**Calculating the EA and IE:**^[17]^

Th electron affinity (EA) and ionization energy (IE) were measured by the electrochemical cyclic voltammetry (CV) and calculated by EA = e(*φ*_red_+4.80-*V*_ferro_) and IE = e(*φ*_ox_+4.80-*V*_ferro_). The CV was conducted on an electrochemical workstation with glassy carbon disk, Pt wire and Ag/Ag^+^ electrode as working electrode, counter electrode and reference electrode respectively in a 0.1 mol L^-1^ tetrabutylammonium hexafluorophosphate (Bu_4_NPF_6_) acetonitrile solution.

‘**The DSC measurements:**^[18-19]^

The melting point depression experiment was carried out using differential scanning calorimetry (DSC, METTLER TOLEDO, DSC3) to evaluate the interaction parameter between the electron acceptors (IEICO-4F, Y6, and BTP-eC9) and PTB7-Th. The samples were firstly heated at a rate of 10 °C/min to 310 °C and held for 3 minutes. Subsequently the samples were cooled to 25 °C at a rate of 10 °C/min, and the melting temperature (*T*_m_) was obtained. The interaction parameter was determined by fitting the melting point depression data based on:

| $\frac{1}{T_{m}}-\frac{1}{T_{m}^{0}}=-\frac{RV_{2u}}{\Delta H_{2u}V_{1u}}\left[ \frac{\ln v_{2}}{m_{2}}+\left( \frac{1}{m_{2}}-\frac{1}{m_{1}} \right)\times\left( 1-v_{2} \right)+\chi_{12}{(1-v_{2})}^{2} \right]$ | (S42) |
| --- | --- |

where *T*_m_^0^ represents the melting point of electron acceptors in the standard state, *T*_m_ is the melting point when mixed with PTB7-Th, *R* is the gas constant, the subscript 1 is identified with PTB7-Th and the subscript 2 with the electron acceptors, *V*_u_ is the molar volume (of repeating unit in the case of polymer), *∆H*_u_ is the enthalpy of fusion per mole (of repeating unit in the case of polymer), *v* is the volume fraction, *m* is the degree of polymerization, and *ꭓ*_12_ represents the interaction parameter between PTB7-Th and the acceptor.’

**Calculating the bimolecular recombination coefficient:** ^[20]^

In organic solar cells, the bimolecular recombination loss has been found to be significantly lower than the traditional Langevin recombination rate. This reduction can be quantified by the bimolecular recombination coefficient (*γ*).

| $\gamma=\gamma_{pre}\frac{q}{\varepsilon_{0}\varepsilon_{r}}(\mu_{h}+\mu_{e})$ | (S43) |
| --- | --- |

where *γ* is the bimolecular recombination coefficient, *γ*_pre_ is the Langevin prefactor, *q* is the elementary charge, *ε*_0_ is the vacuum permittivity, *ε*_r_ is the relative permittivity of the photoactive layer, taken as 3, *μ*_e_ is the electron mobility and *μ*_h_ is the hole mobility. The calculation of *γ* is dependent on the bimolecular recombination efficiency (*η*_BR_).

| $\eta_{BR}=\frac{\gamma p^{2}\mu_{h}}{G\mu_{e}}=\beta^{-1}-1$ | (S44) |
| --- | --- |

*η*_BR_ is the bimolecular recombination efficiency, *p* is the hole carrier density, *G* is the exciton generation rate, calculated using *J*_sat_, and *β* is the light-intensity dependence coefficient of the current, derived from the light-intensity-dependent *J*_SC_. The hole carrier density can be calculated using the following equation:

| $p=\frac{f_{p}\mu_{e}}{2\gamma\mu_{h}}\left[ \sqrt{1+\frac{4\gamma\mu_{h}G}{{f_{p}^{2}\mu}_{e}}}-1 \right]$ | (S45) |
| --- | --- |

*f*_p_ is the hole extraction rate. The rate *f*_p_ can be decomposed into contributions from drift and diffusion components:

| $f_{p}=f_{p}^{diffusion}+f_{p}^{drift}=8\frac{\mu_{h}k_{B}T}{qd^{2}}+2\frac{\mu_{h}V_{0}}{d^{2}}$ | (S46) |
| --- | --- |

*k*_B_ is the Boltzmann constant, *T* is the temperature, *V*_0_ is the built-in electric field, which can be approximated by *V*_oc_ under short-circuit conditions, and the *d* is the thickness of the photoactive layer.

**Calculating the *L*_dr_:** ^[21-22]^

Bartesaghi et al. proposed a figure-of-merit *θ* to evaluate the ratio between carrier recombination and extraction rates, as well as to calculate the carrier drift length (*l*_dr_) at short-circuit current conditions.

| $\theta=\frac{k_{rec}}{k_{ext}}=\frac{\gamma Gd^{4}}{\mu_{h}\mu_{e}V_{0}^{2}}=\frac{d^{2}}{l_{dr}^{2}}$ | (S47) |
| --- | --- |

where *k*_rec_ represents carrier recombination rate, *k*_ext_ is the carrier extraction rate, *γ* represents the bimolecular recombination coefficient, *G* represents exciton generation rate, calculated using *J*_sat_, *d* is the thickness of the active layer, *μ*_e_ is the electron mobility, *μ*_h_ is the hole mobility, *V*_0_ represents internal voltage, which can be substituted by *V*_OC_ at the short-circuit current condition and *l*_dr_ is the carrier drift length.
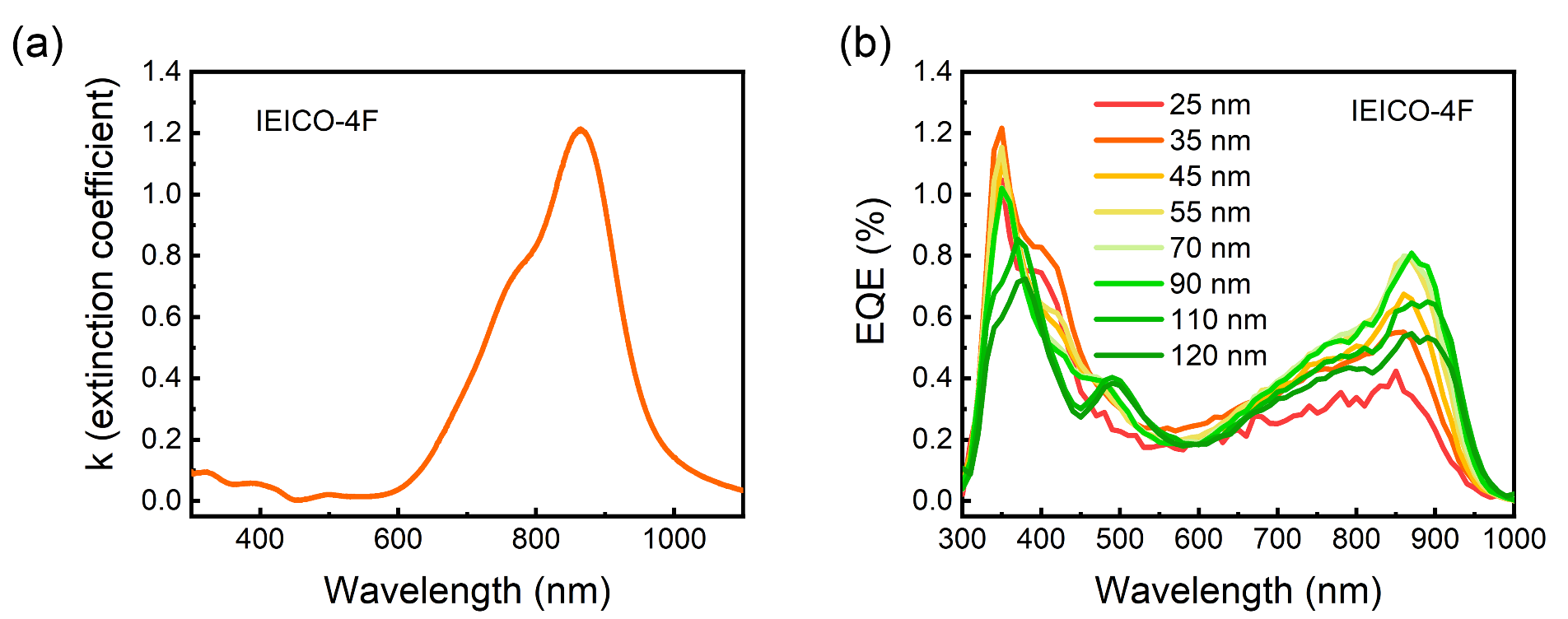


**Figure S1.** (a) The extinction coefficient of IEICO-4F. (b) EQE spectra of CuSCN/IEICO-4F devices at different IEICO-4F thicknesses (25nm, 35nm, 45nm, 55nm, 70nm, 90nm, 110nm, and 120nm).


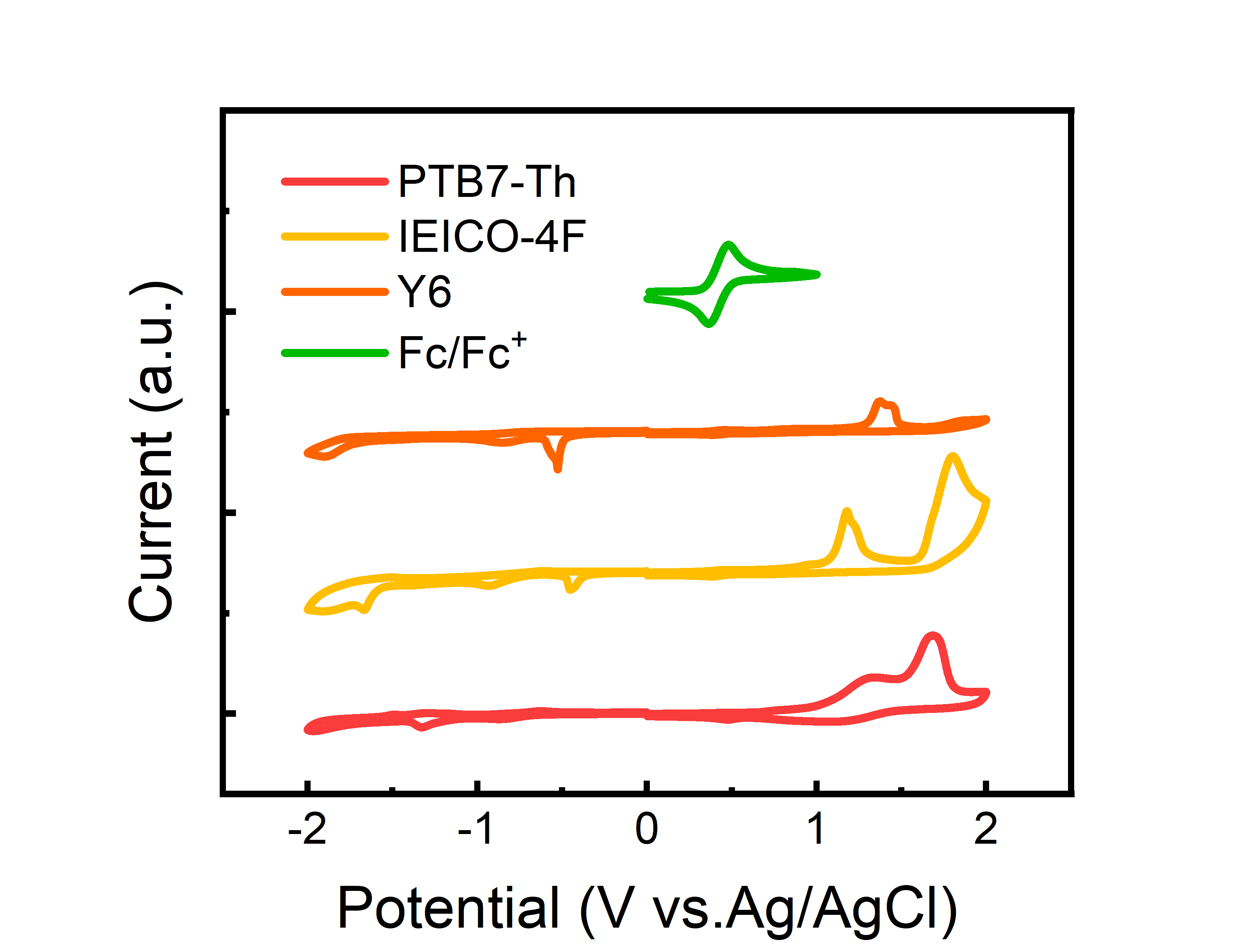


**Figure S2.** The CV measurement of PTB7-Th, IEICO-4F, Y6, and Fc/Fc^+^.


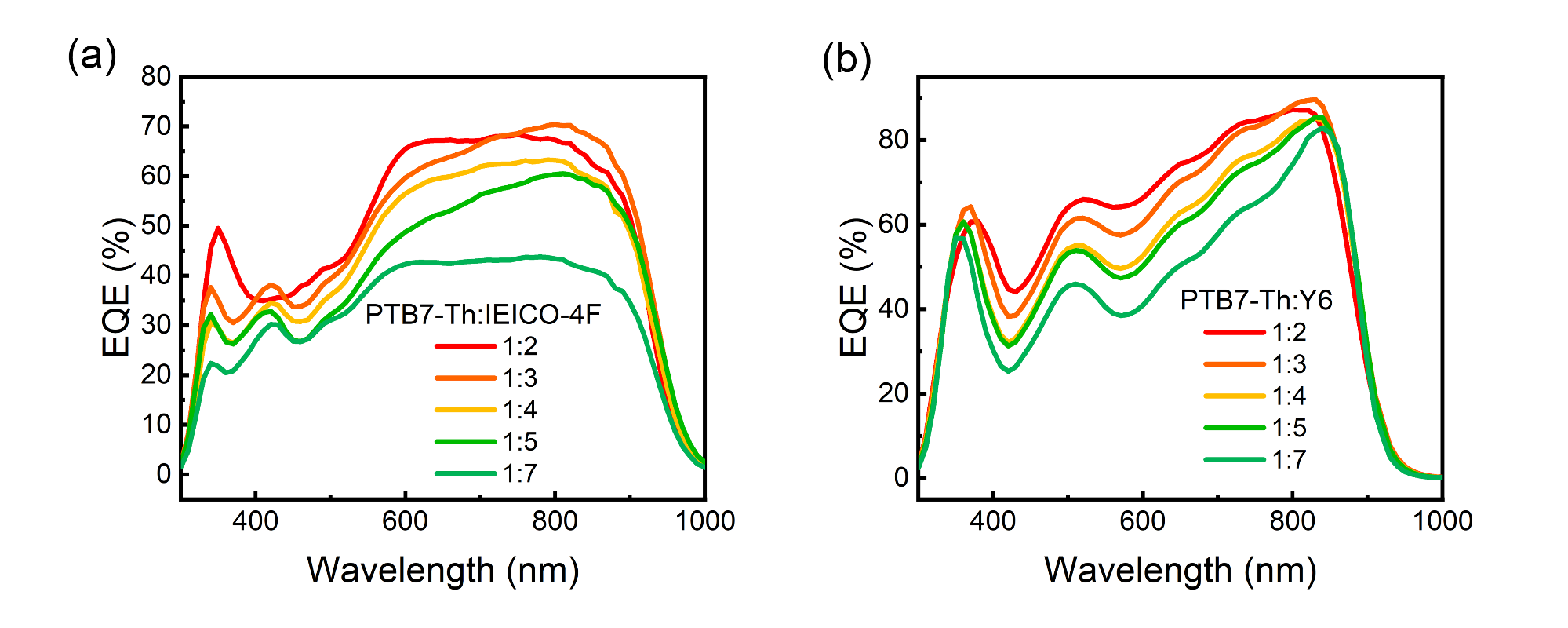


**Figure S3.** EQE curves of (a) PTB7-Th:IEICO-4F and (b) PTB7-Th:Y6 devices at various D/A ratios (1:2, 1:3, 1:4, 1:5, and 1:7).


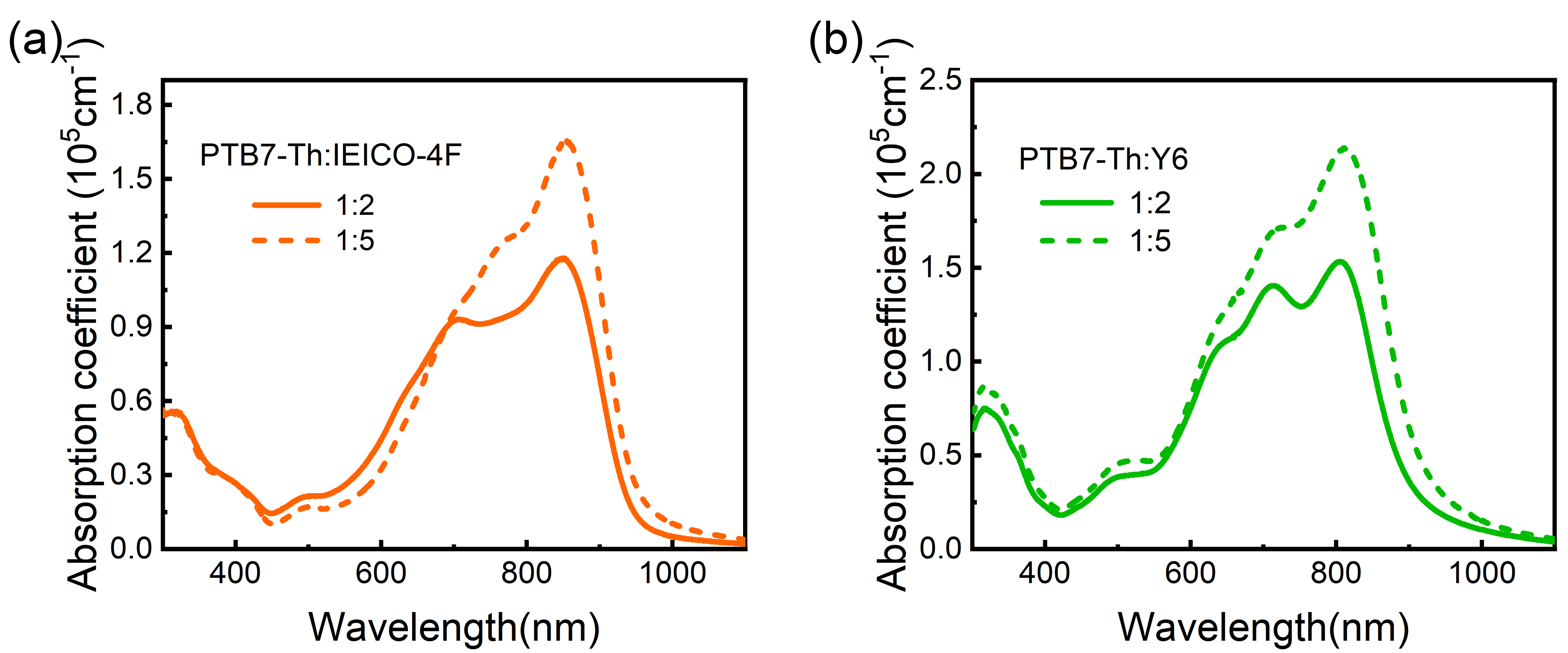


**Figure S4.** Absorption coefficients of (a) PTB7-Th:IEICO-4F and (b) PTB7-Th:Y6 blend films at D/A ratios of 1:2 and 1:5.


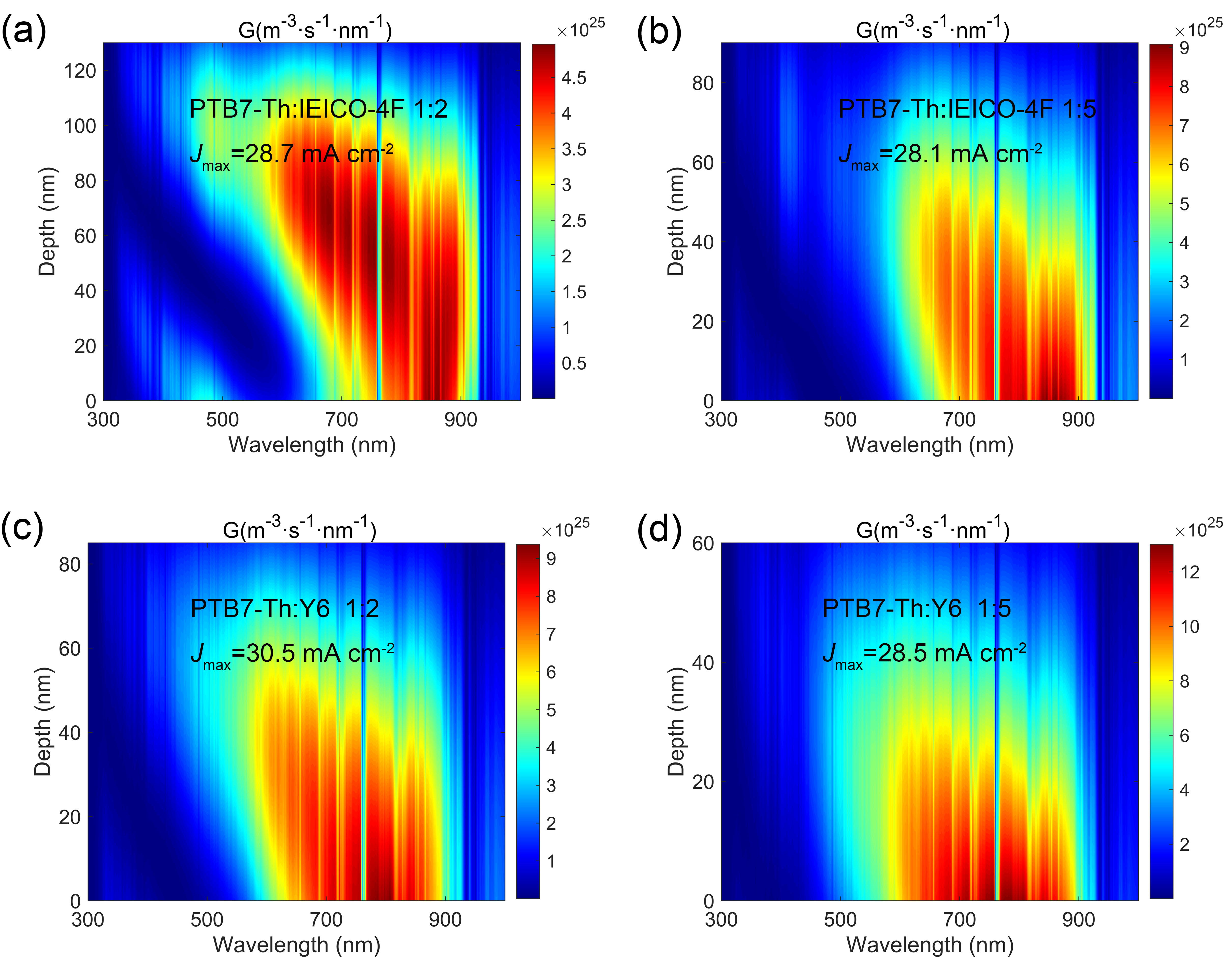


**Figure S5.** Simulated exciton generation profiles and *J*_MAX_ values of PTB7-Th:IEICO-4F and PTB7-Th:Y6 devices at D/A ratios of 1:2 and 1:5.


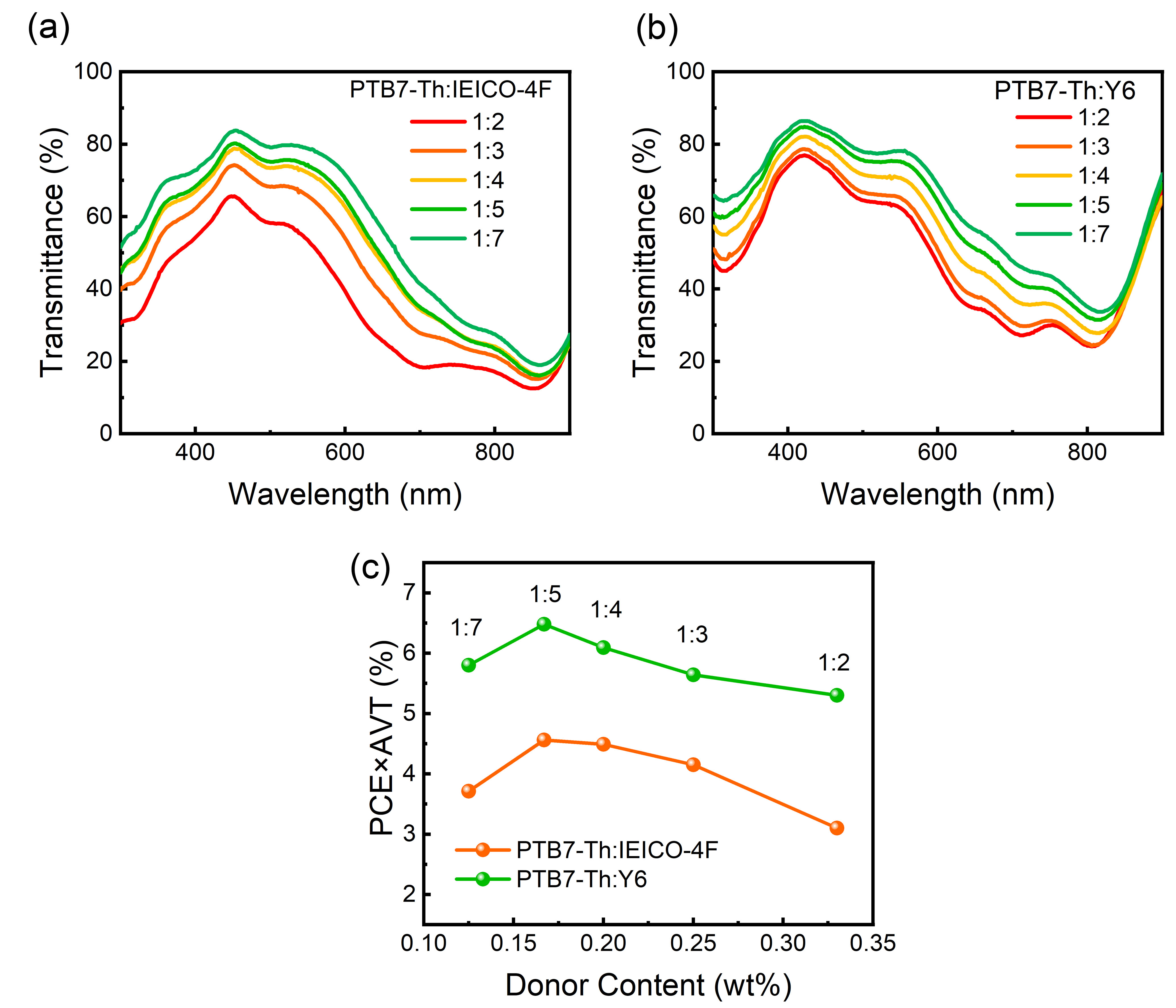


**Figure S6.** Transmittance spectra of (a) PTB7-Th:IEICO-4F and (b) PTB7-Th:Y6 blend films at various D/A ratios (1:2, 1:3, 1:4, 1:5, and 1:7). (c) The product of PCE for the opaque device and AVT for the blend film of PTB7-Th:IEICO-4F and PTB7-Th:Y6 at various D/A ratios (1:2, 1:3, 1:4, 1:5, and 1:7).


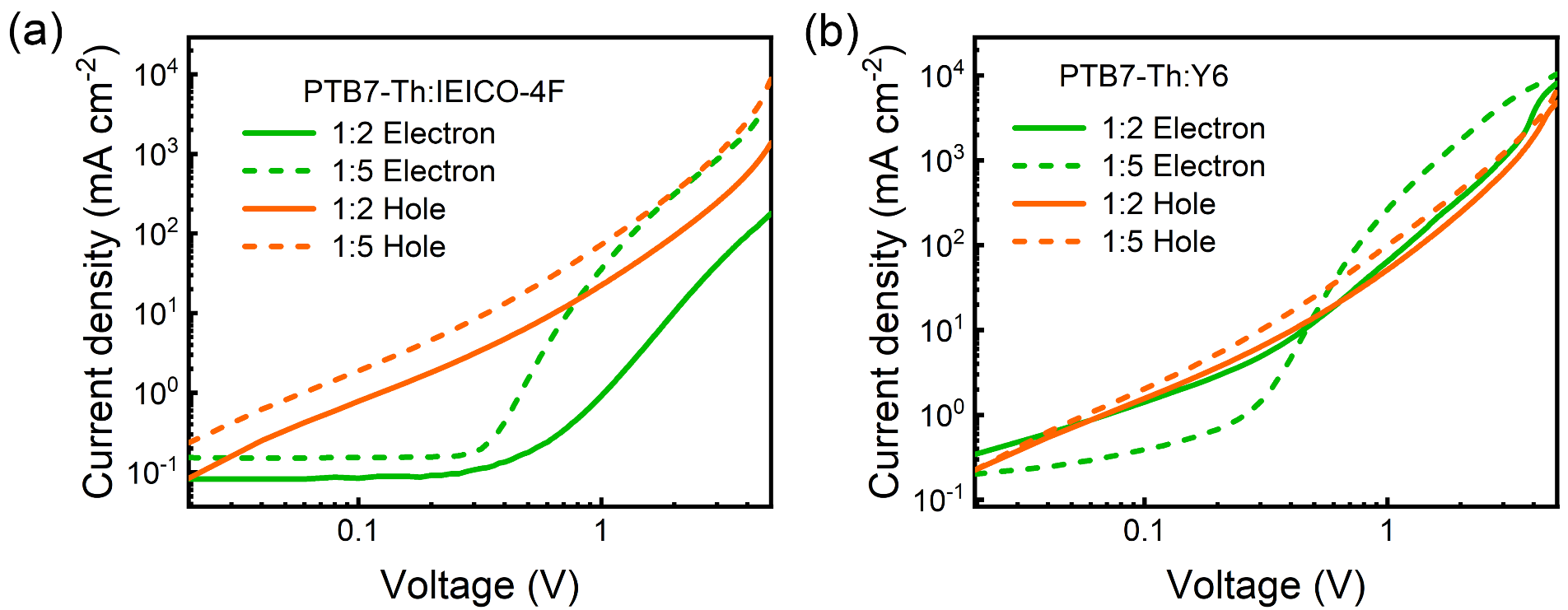


**Figure S7.** Hole-only current density and electron-only current density versus voltage curves of (a) PTB7-Th:IEICO-4F and (b) PTB7-Th:Y6 devices at D/A ratios of 1:2 and 1:5.


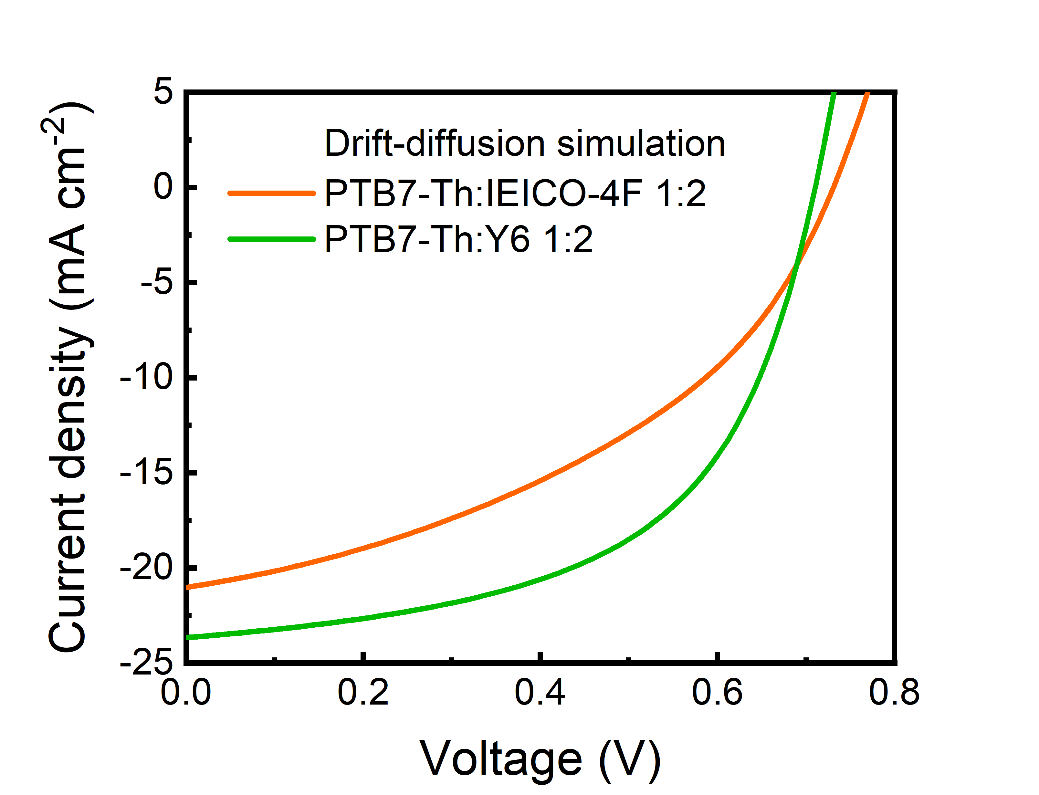


**Figure S8.** *J*-*V* curves of PTB7-Th:IEICO-4F and PTB7-Th:Y6 devices at a D/A ratio of 1:2 via drift-diffusion simulation.


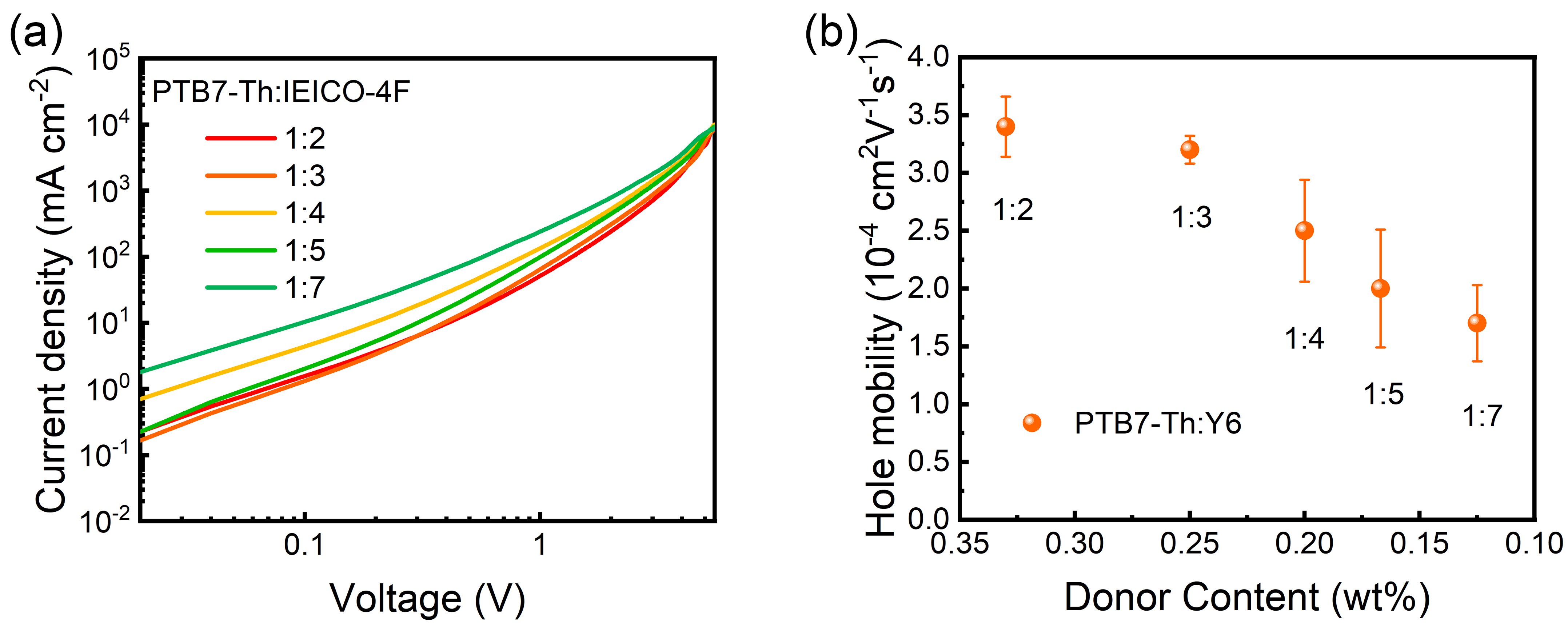


**Figure S9.** (a) Hole-only current density versus voltage curves and (b) hole mobilities of PTB7-Th:Y6 devices at various D/A ratios (1:2, 1:3, 1:4, 1:5, and 1:7).


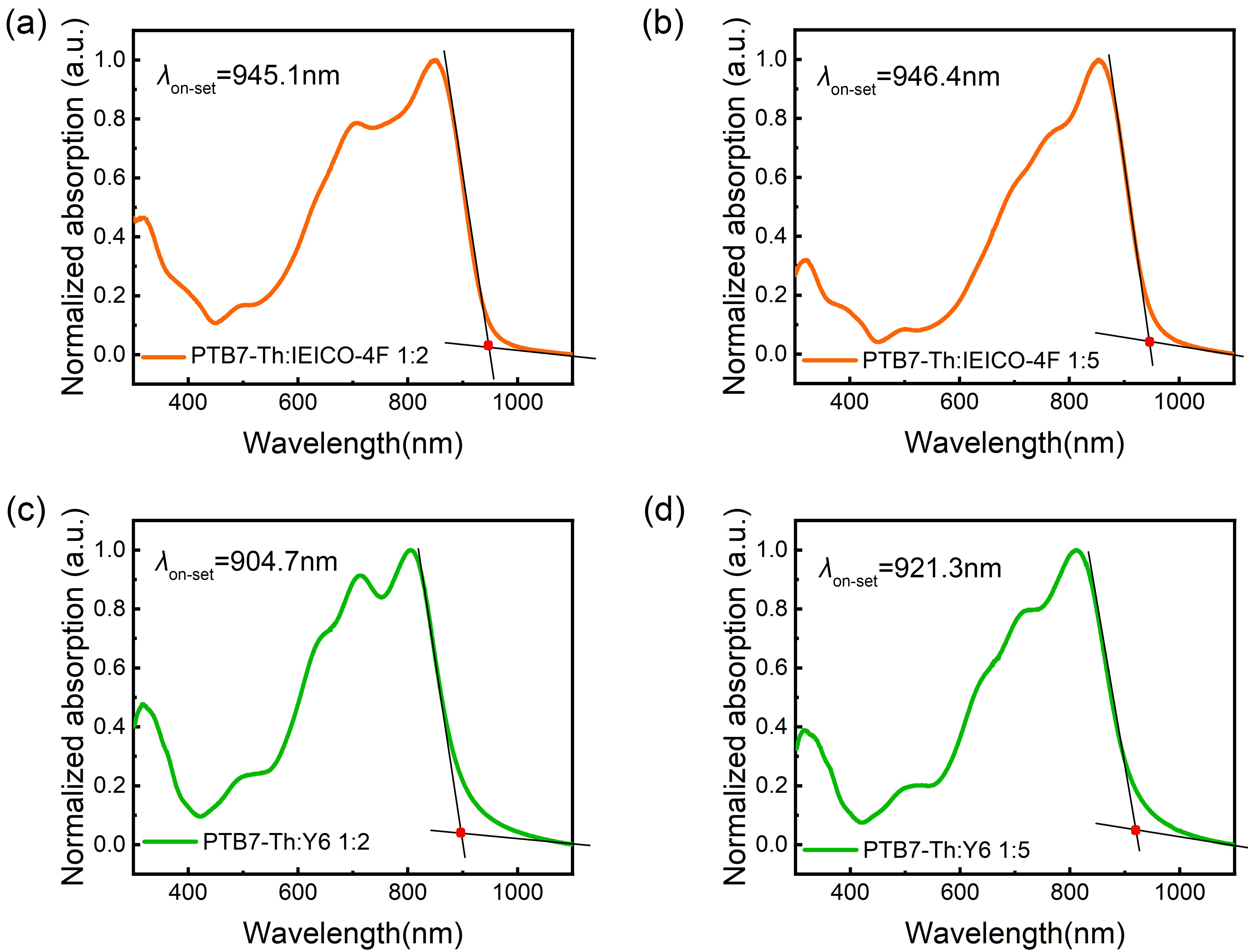


**Figure S10.** *E*_g_ of PTB7-Th:IEICO-4F and PTB7-Th:Y6 blend films at D/A ratios of 1:2 and 1:5.


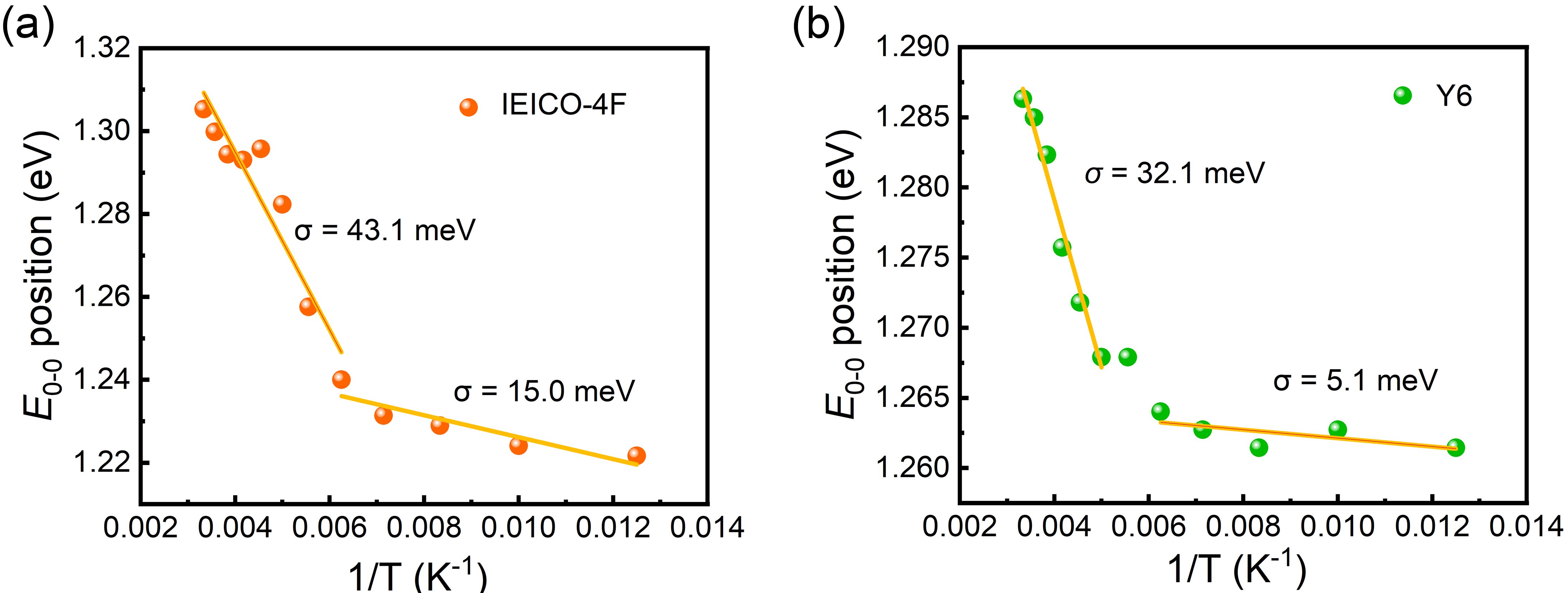


**Figure S11.** Temperature dependence of PL (0-0) position and calculated energy disorders of pure (a) IEICO-4F and (b) Y6 films.


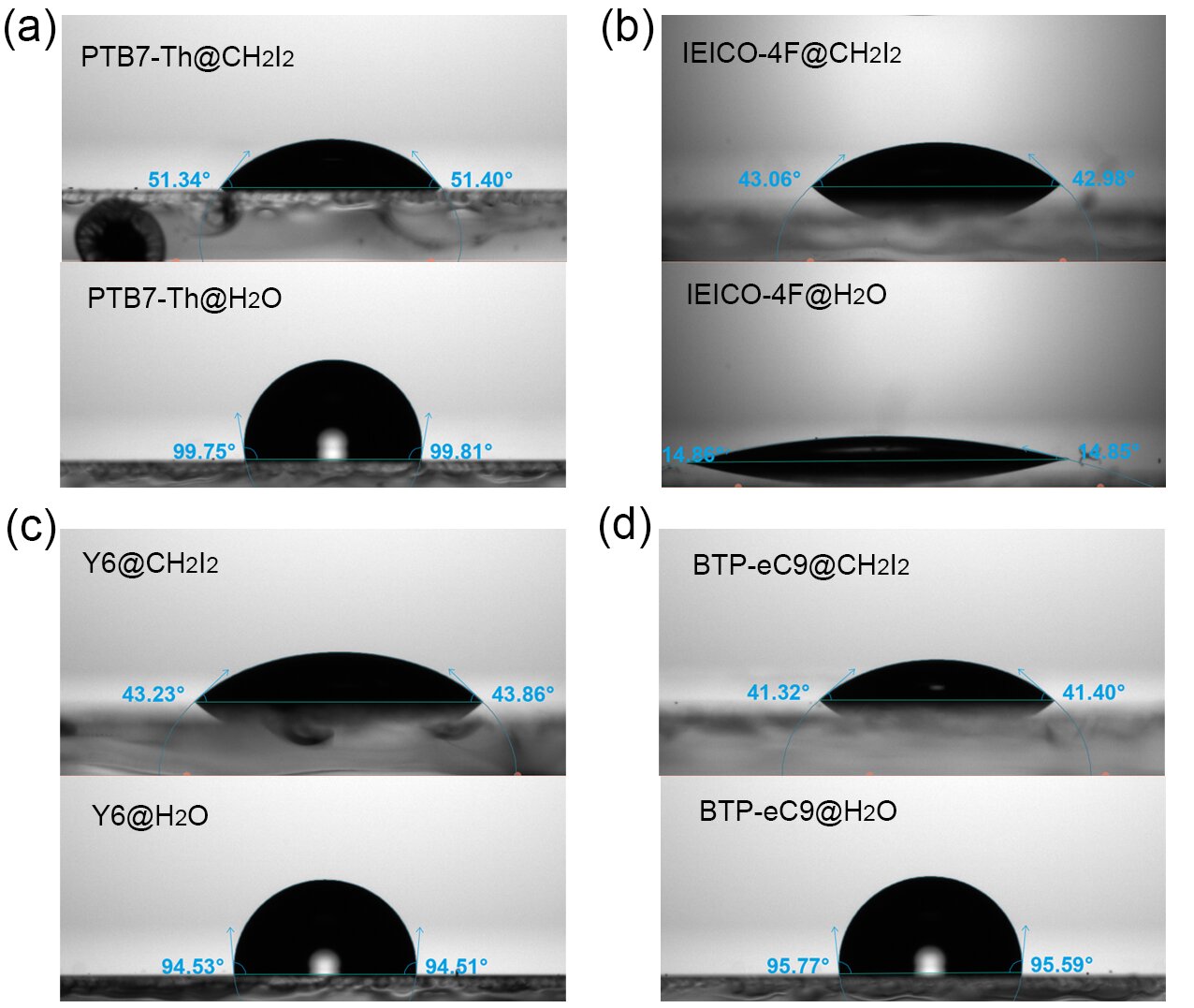


**Figure S12.** Contact angle measurements of PTB7-Th, IEICO-4F, Y6 and BTP-eC9.


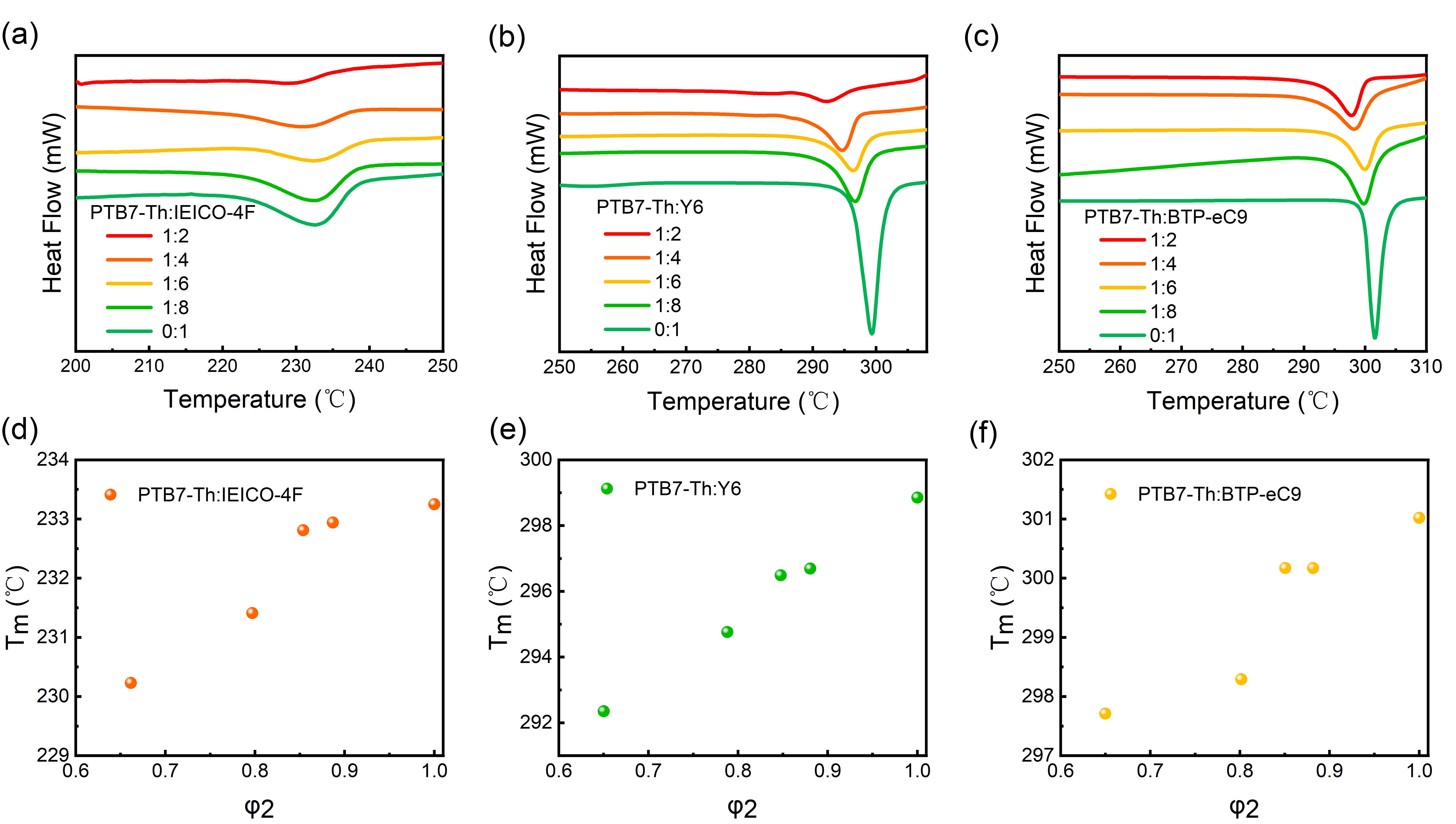


**Figure S13.** Differential scanning calorimetry (DSC) endothermal curves of (a) IEICO-4F, (b) Y6 and (c) BTP-eC9 with different mass ratio of PTB7-Th to show melting point depression behavior. (d-f) The melting temperature (*T*_m_) of the acceptor as a function of its volume fraction in the blend.


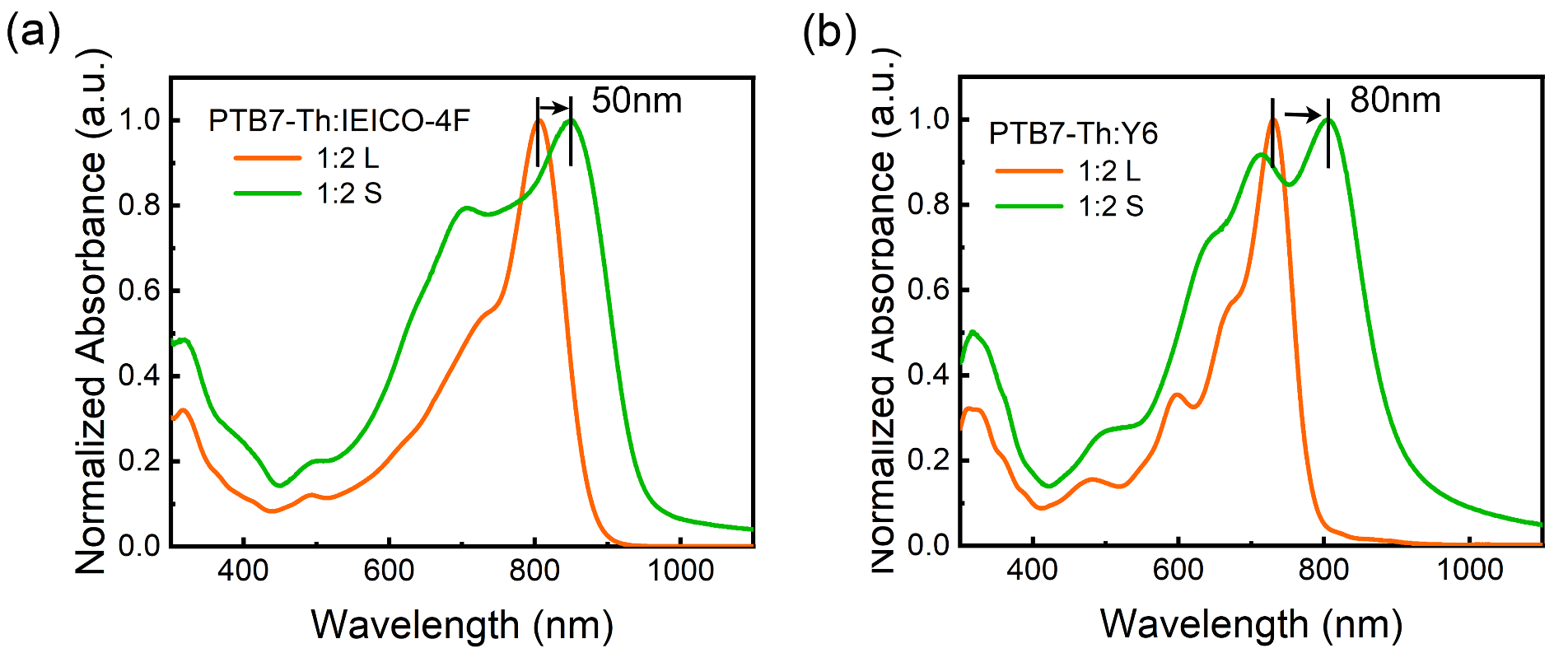


**Figure S14.** Normalized absorbance of PTB7-Th:IEICO-4F and PTB7-Th:Y6 blend solutions and films at a D/A ratio of 1:2.


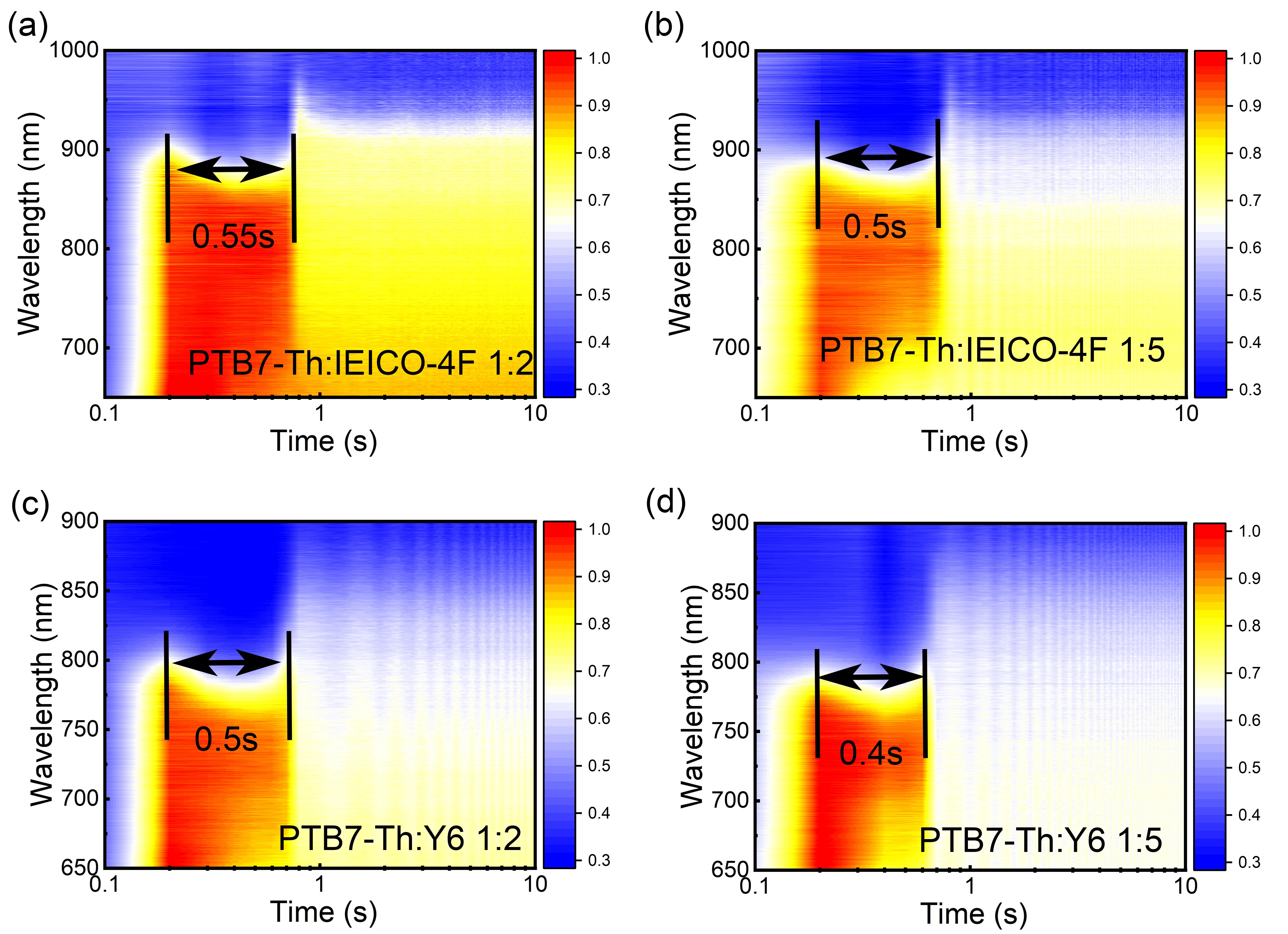


**Figure S15.** Contour plots of In-situ absorption spectra of PTB7-Th:IEICO-4F and PTB7-Th:Y6 blend films at D/A ratios of 1:2 and 1:5.


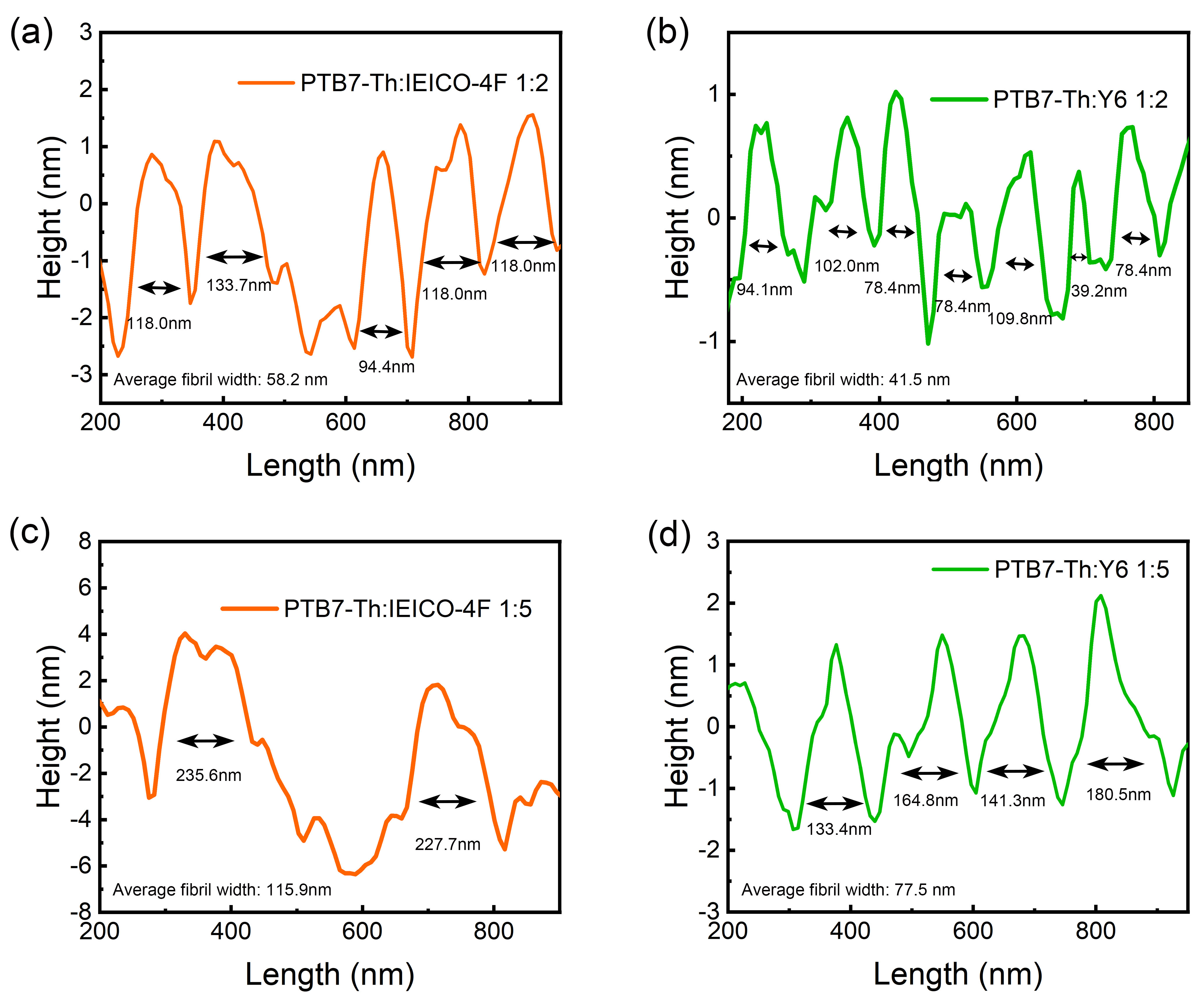


**Figure S16.** Line profiles along the diagonal direction in AFM images to obtain the average fibril width of PTB7-Th:IEICO-4F and PTB7-Th:Y6 blend films at D/A ratios of 1:2 and 1:5.

**
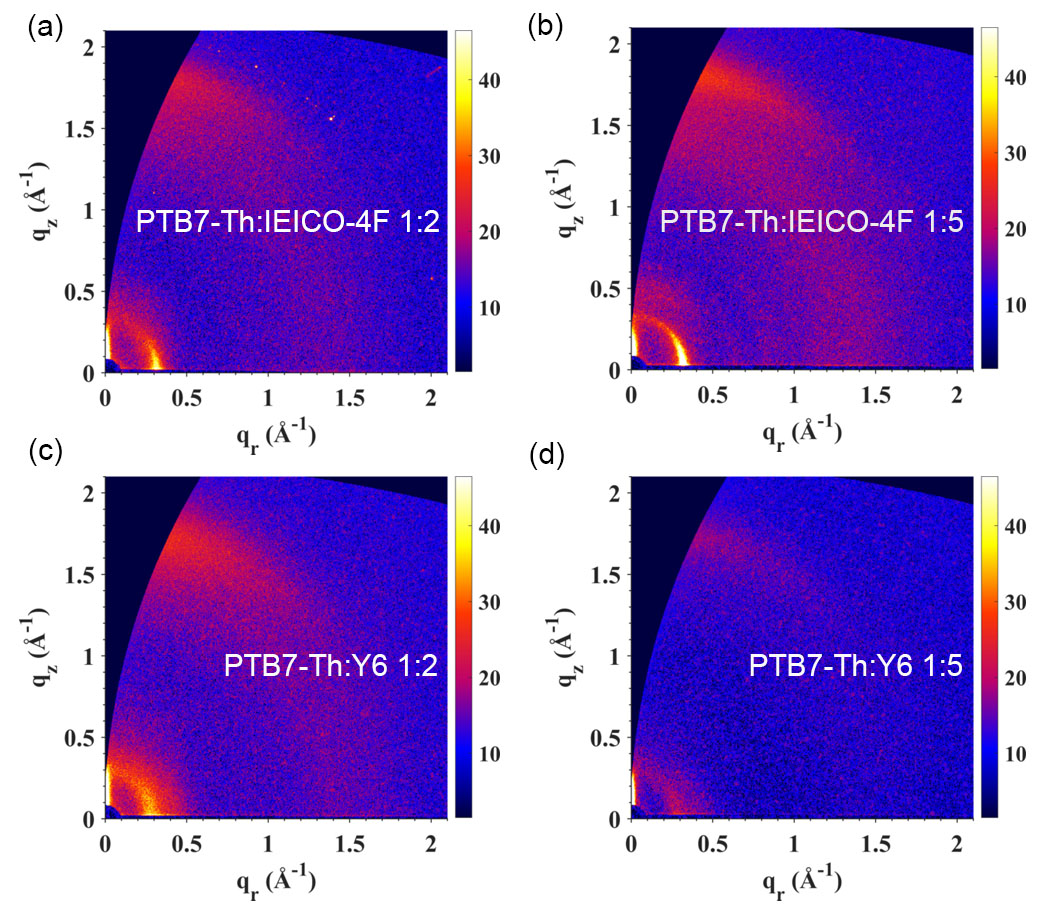
**

**Figure S17.** Two-dimensional GIWAXS scattering patterns of PTB7-Th:IEICO-4F and PTB7-Th:Y6 blend films at D/A ratios of 1:2 and 1:5.


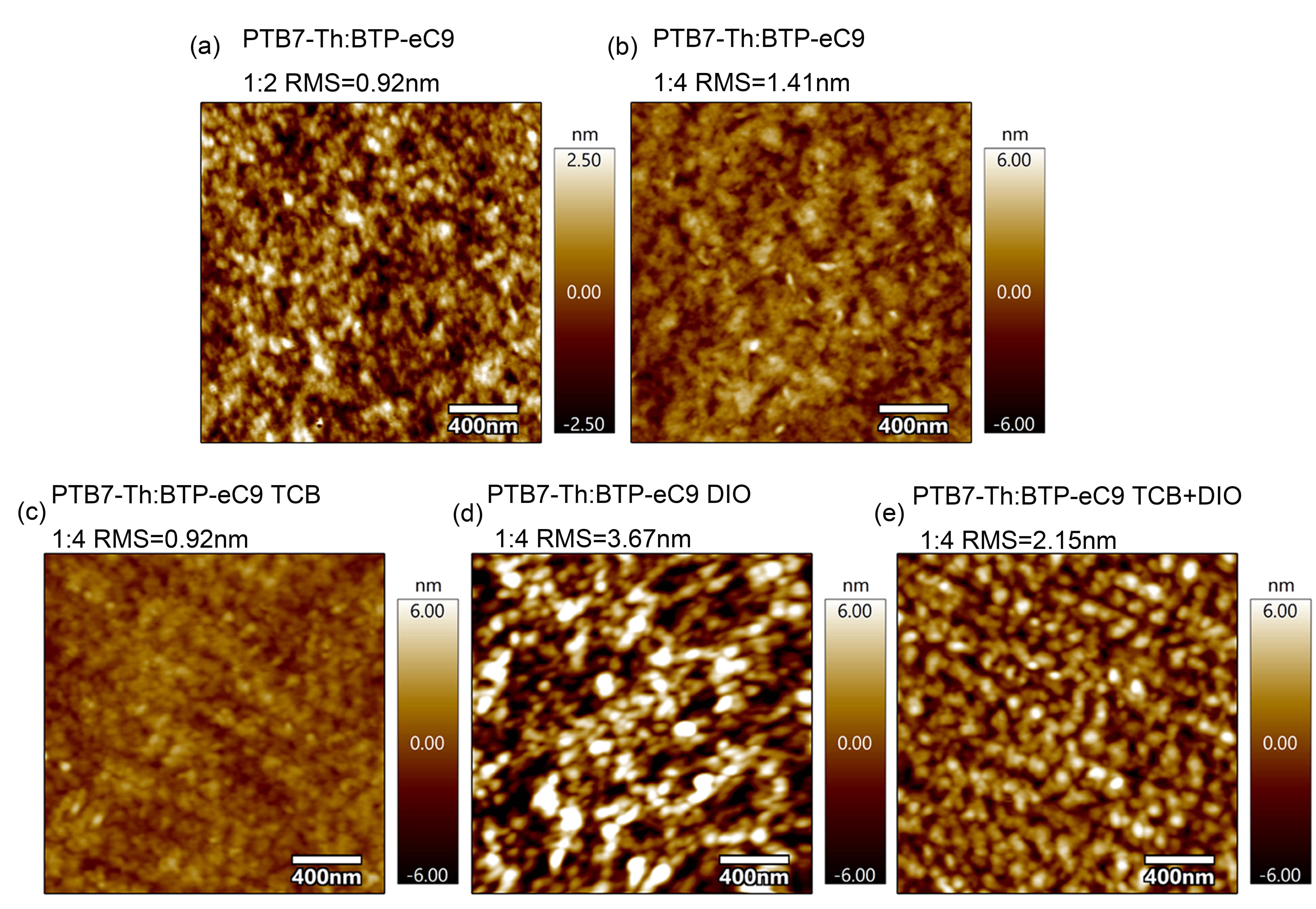


**Figure S18.** AFM images of PTB7-Th:BTP-eC9 blend films at D/A ratios of 1:2 and 1:4 with and without the addition of TCB and DIO.

**
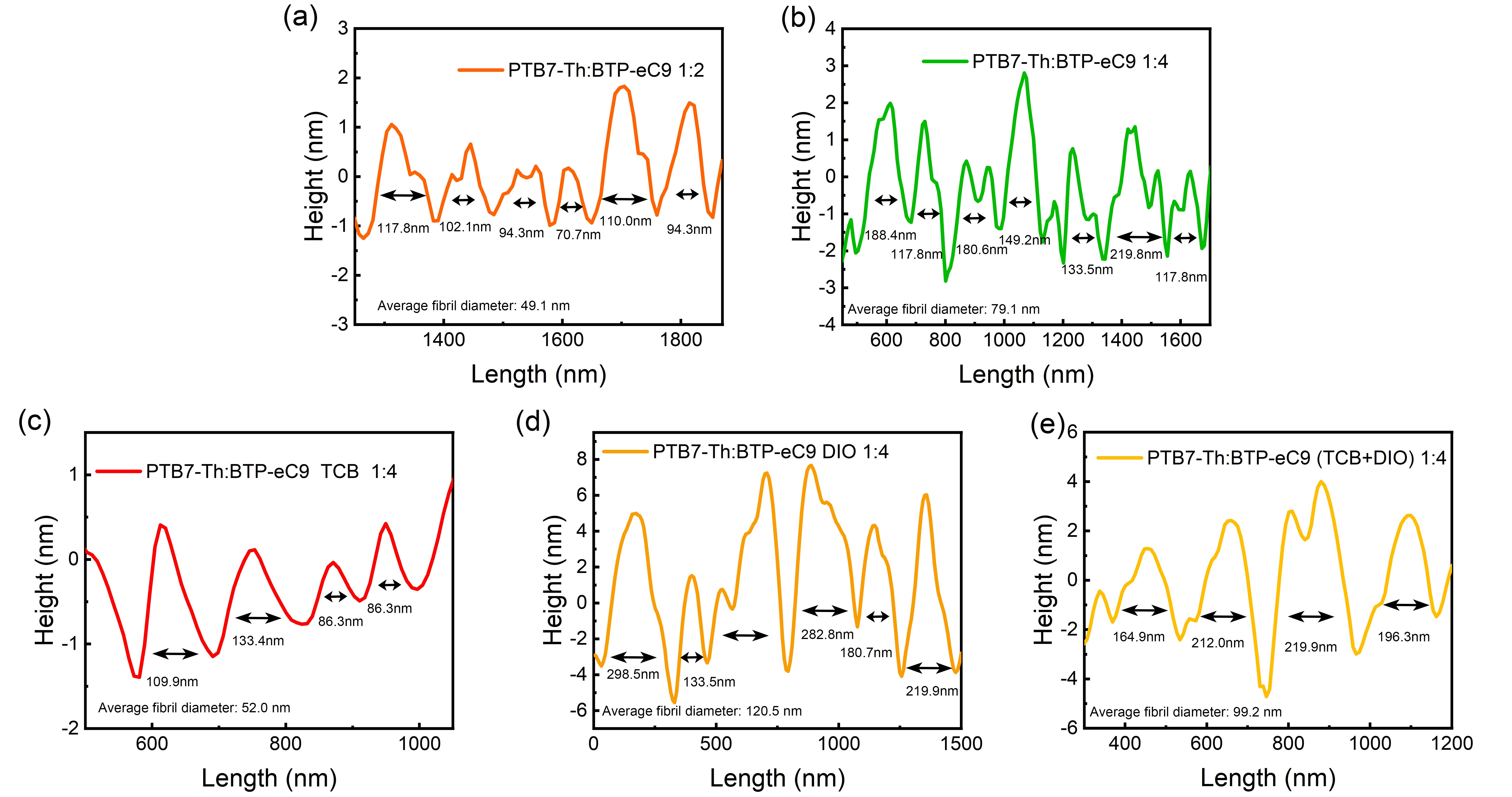
**

**Figure S19.** Line profiles along the diagonal direction to obtain the average fibril width of PTB7-Th:BTP-eC9 blend films at D/A ratios of 1:2 and 1:4 with and without the addition of TCB and DIO.

**
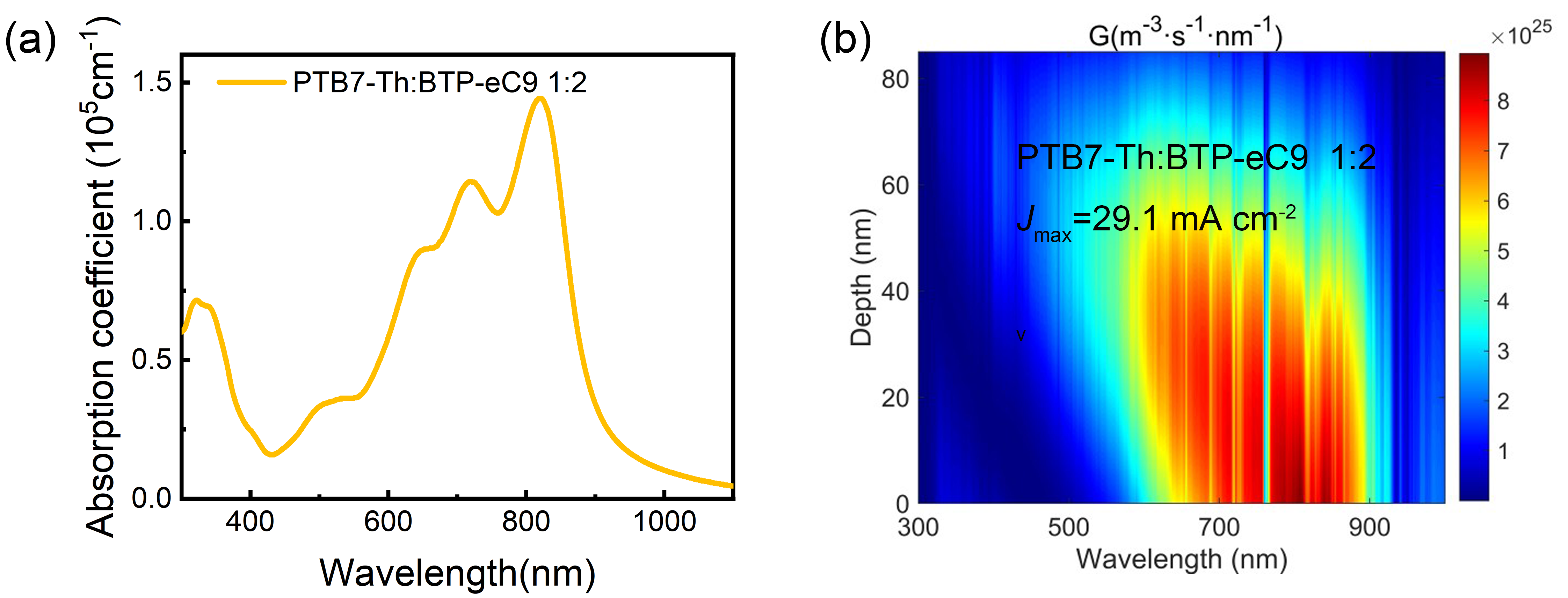
**

**Figure S20.** (a) The absorption coefficient and (b) simulated exciton generation profile and *J*_MAX_ value of PTB7-Th:BTP-eC9 device at a D/A ratio of 1:2.


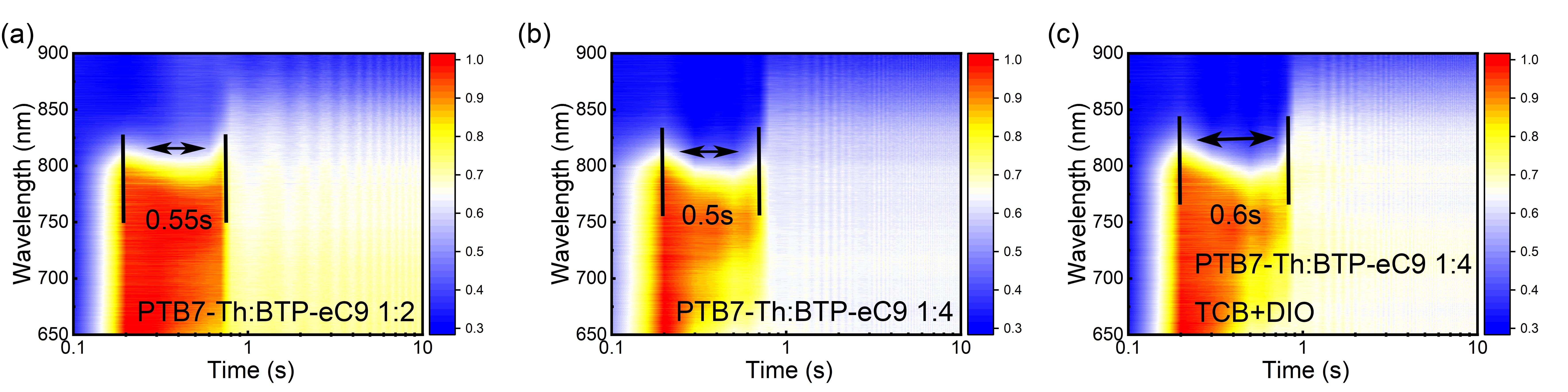


**Figure S21.** Contour plots of In-situ absorption spectra of PTB7-Th:BTP-eC9 device at a D/A ratio of 1:4 with and without the addition of TCB and DIO.


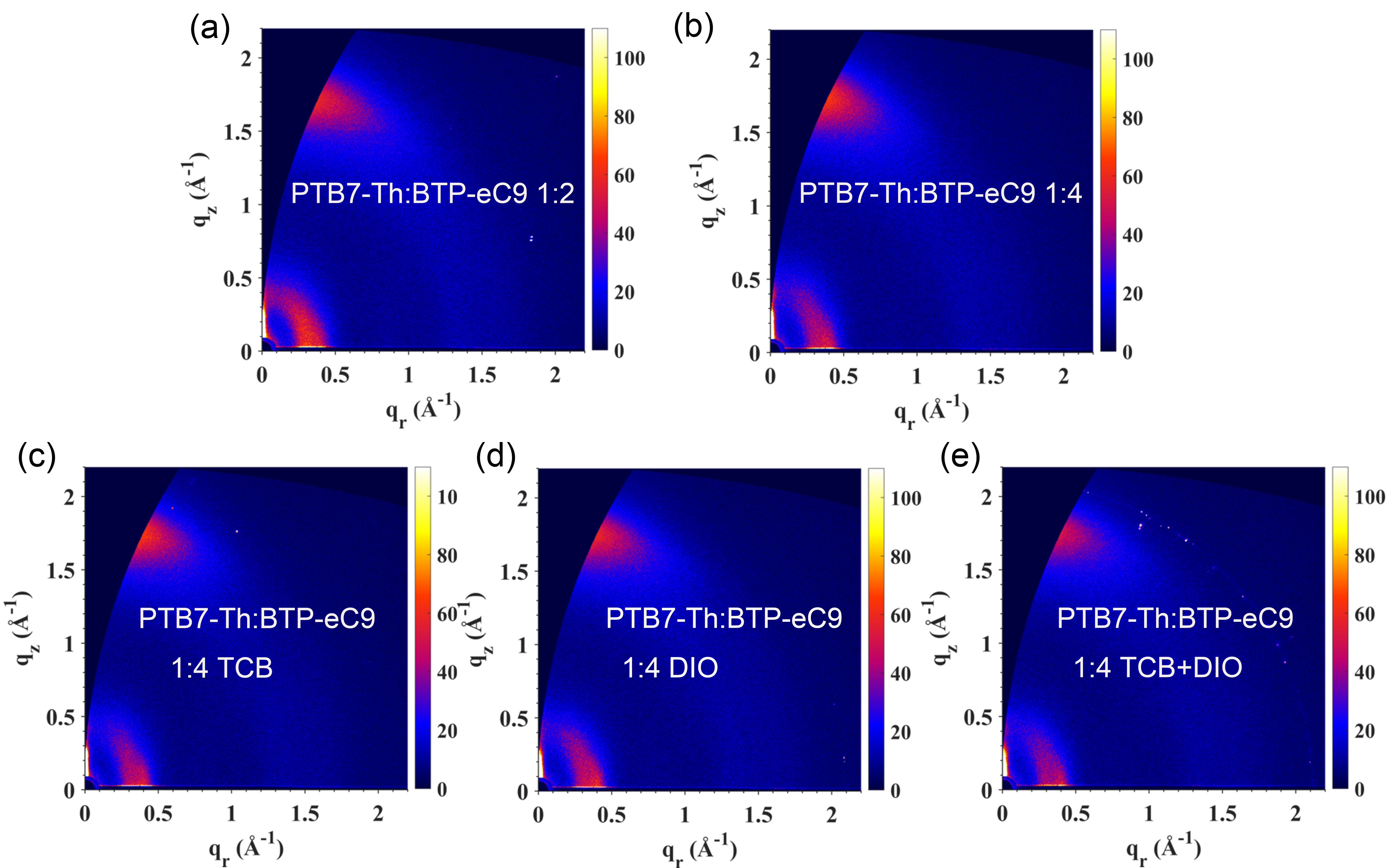


**Figure S22.** Two-dimensional GIWAXS scattering patterns of PTB7-Th:BTP-eC9 blend films at D/A ratios of 1:2 and 1:4 with and without the addition of TCB and DIO.

**
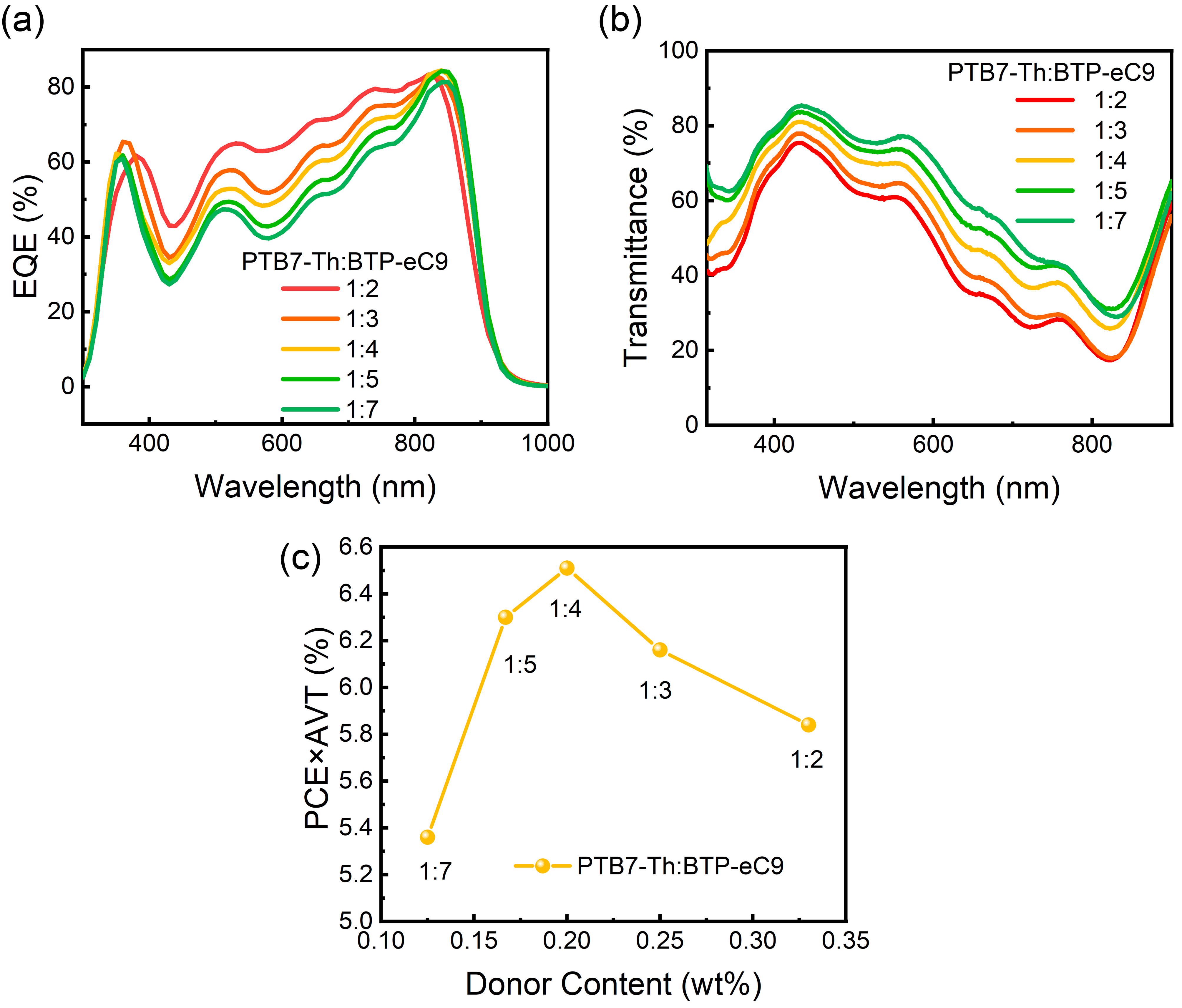
**

**Figure S23.** (a) EQE curves, (b) transmittance spectra, and (c) the product of PCE for the opaque device and AVT for the blend film of PTB7-Th:BTP-eC9 at various D/A ratios (1:2, 1:3, 1:4, 1:5, and 1:7).

**
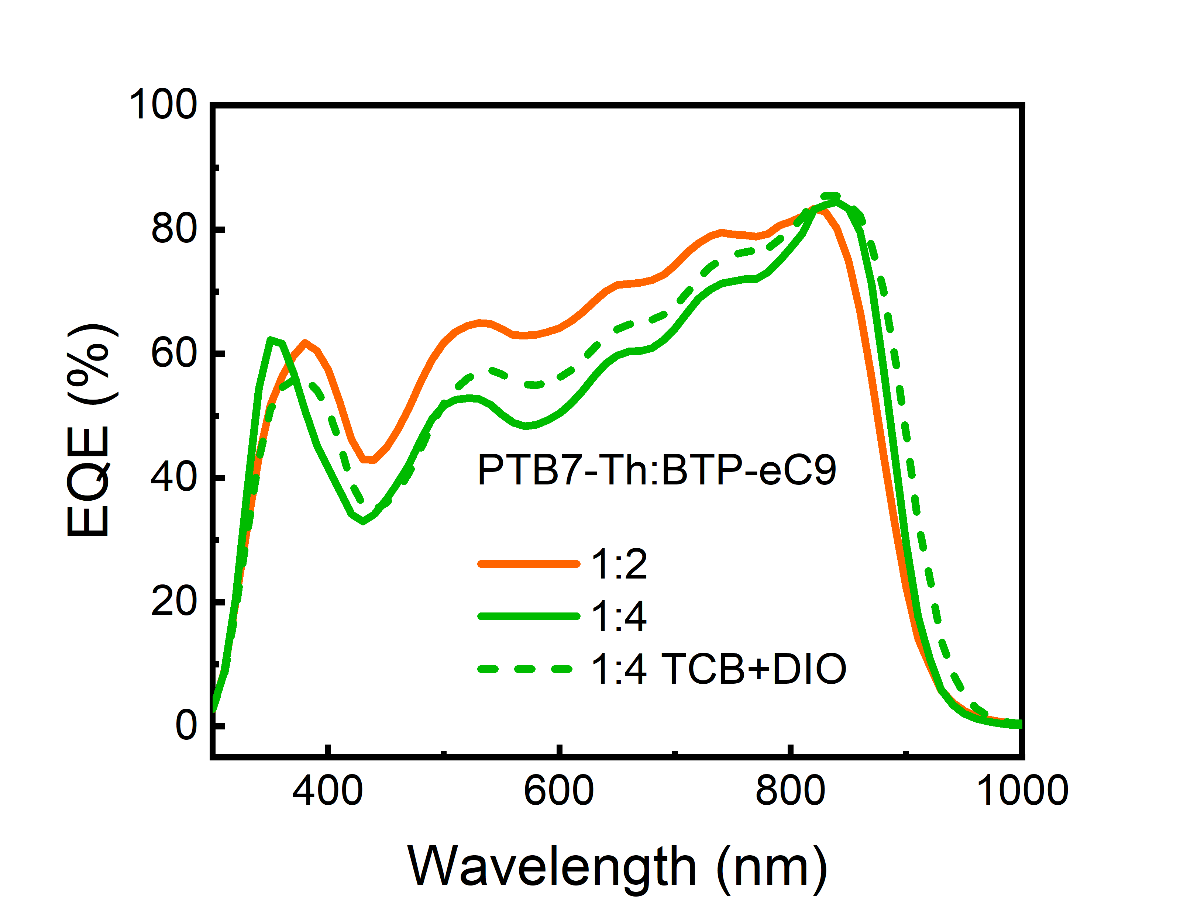
**

**Figure S24.** EQE curves of PTB7-Th:BTP-eC9 (1:2) devices without additives, and PTB7-Th:BTP-eC9 (1:4) devices both with and without dual-additive treatment.

**
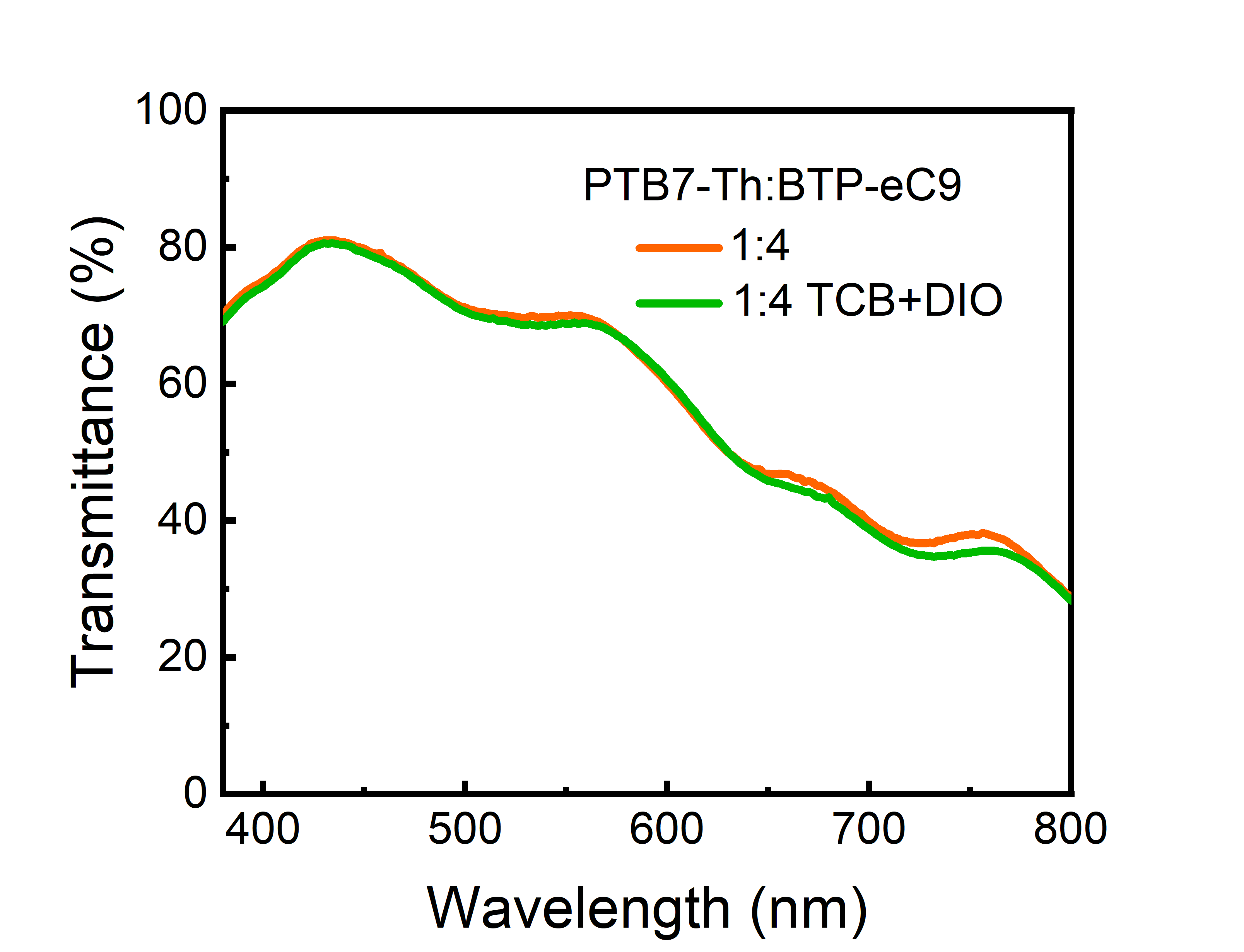
**

**Figure S25.** Transmittance spectra of PTB7-Th:BTP-eC9 (1:4) blend films with and without dual-additive treatment.


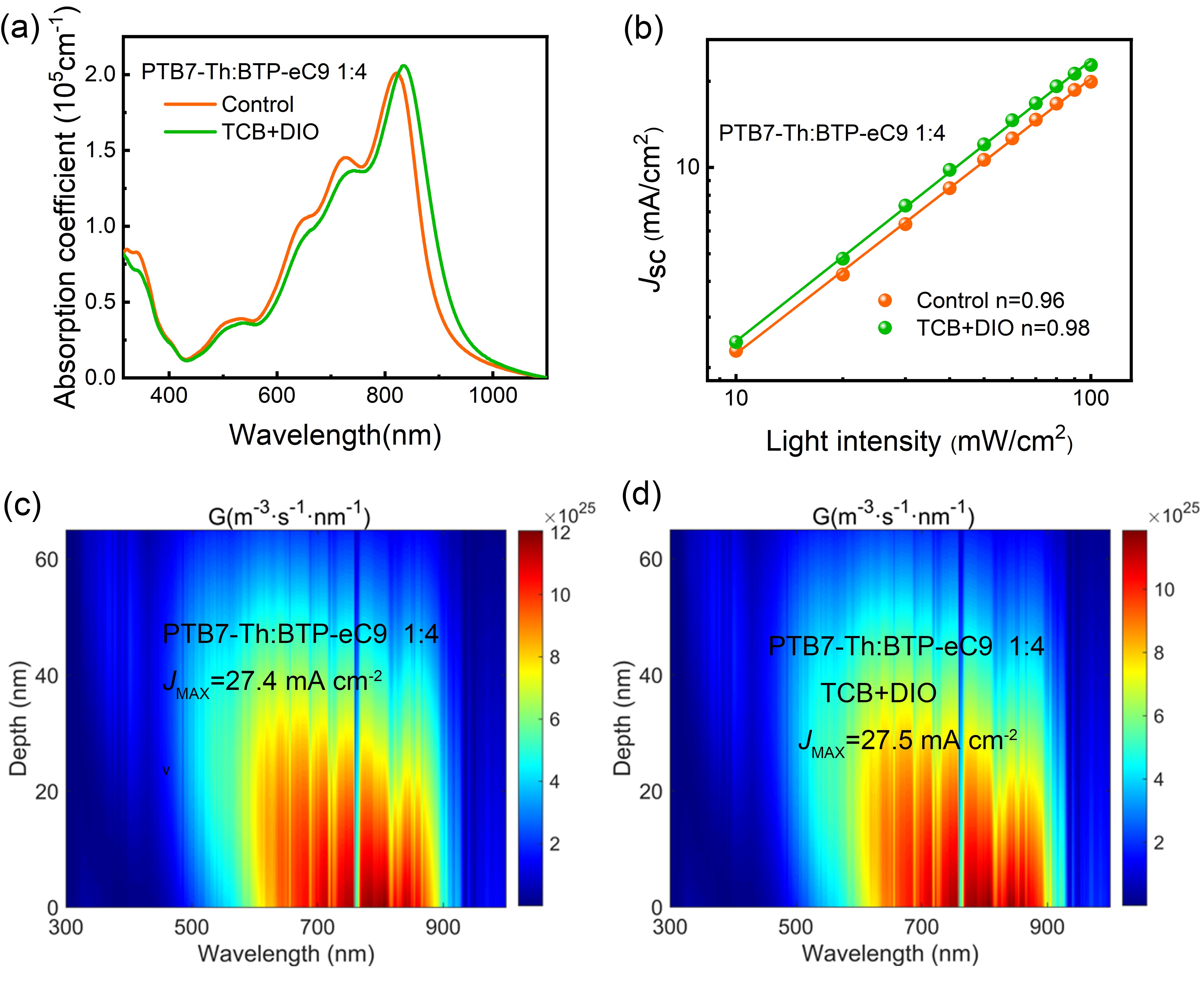


**Figure S26.** (a) The absorption coefficient, (b) *J*_SC_ versus Plight plots and (c-d) simulated exciton generation profiles and *J*_MAX_ values of PTB7-Th:BTP-eC9 device at a D/A ratio of 1:4 with and without the addition of TCB and DIO.


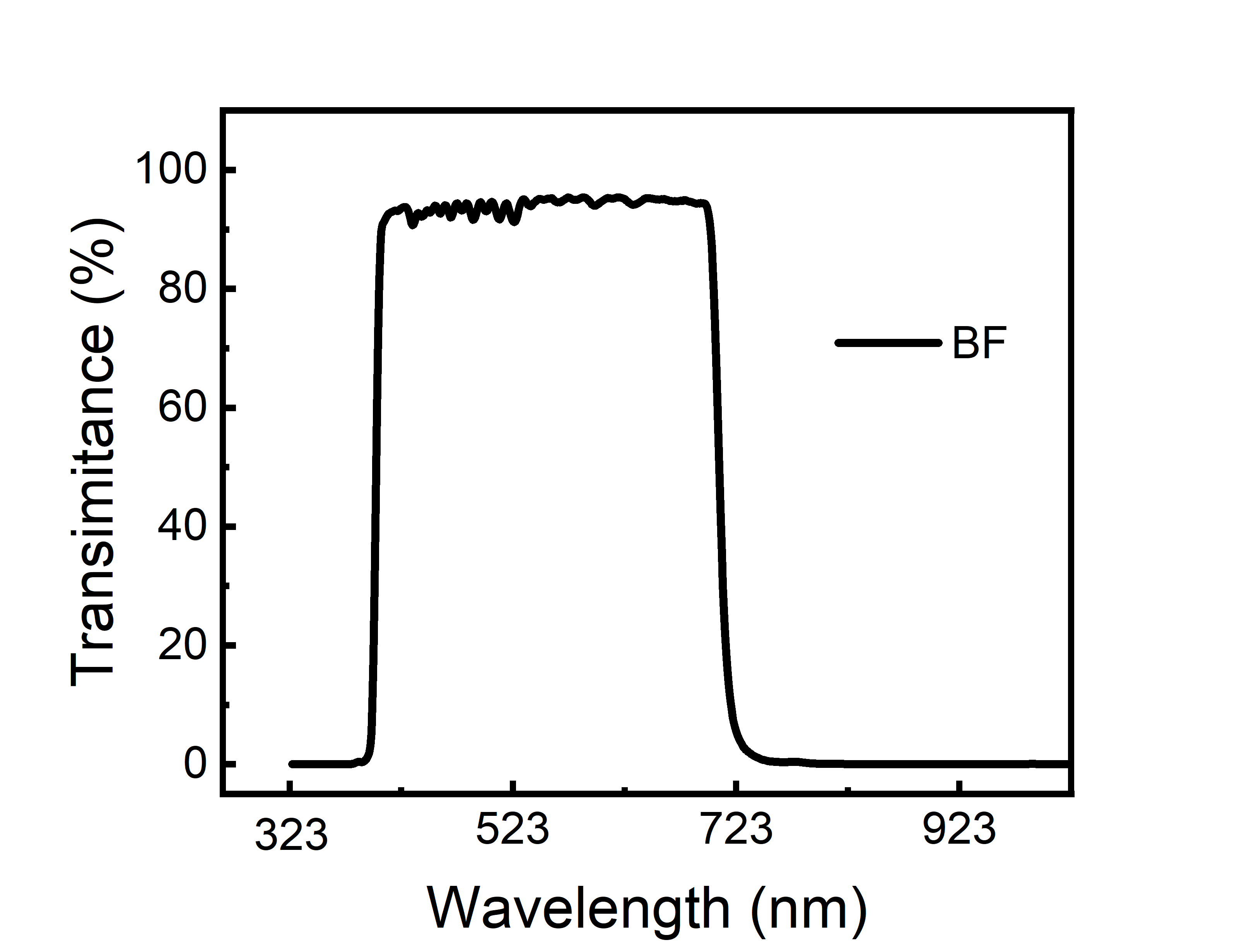


**Figure S27.** Transmittance spectrum of the bandpass filter (BF).


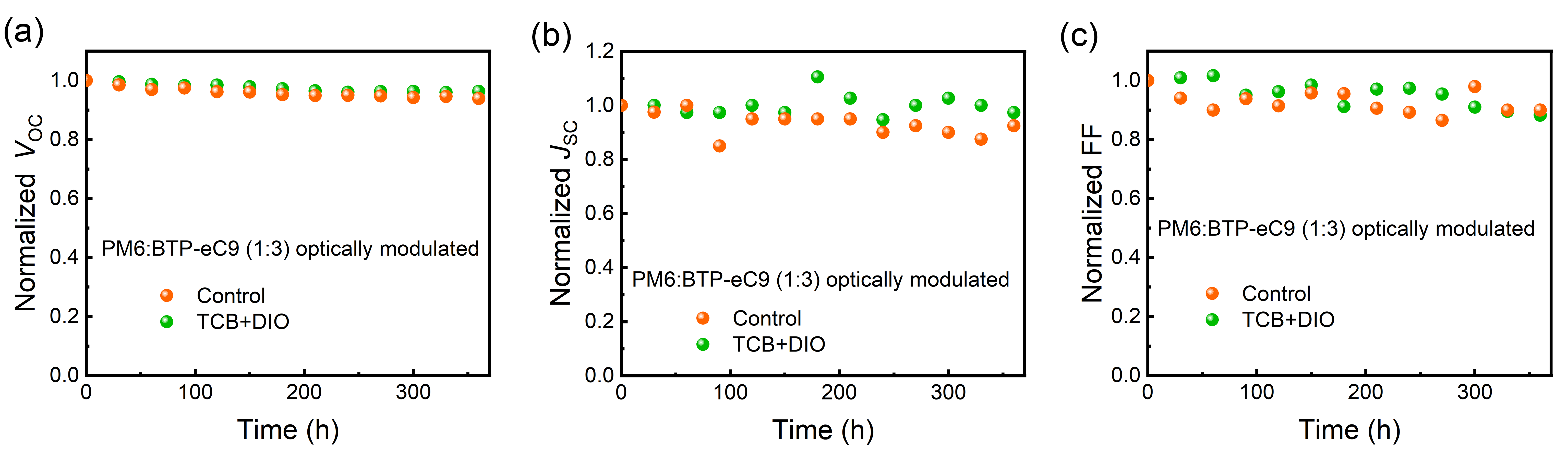


**Figure S28.** Evolution of normalized *V*_OC_, *J*_SC_ and FF decay of optically modulated PM6:BTP-eC9 (1:3) ST-OSCs before and after the addition of TCB and DIO.

**
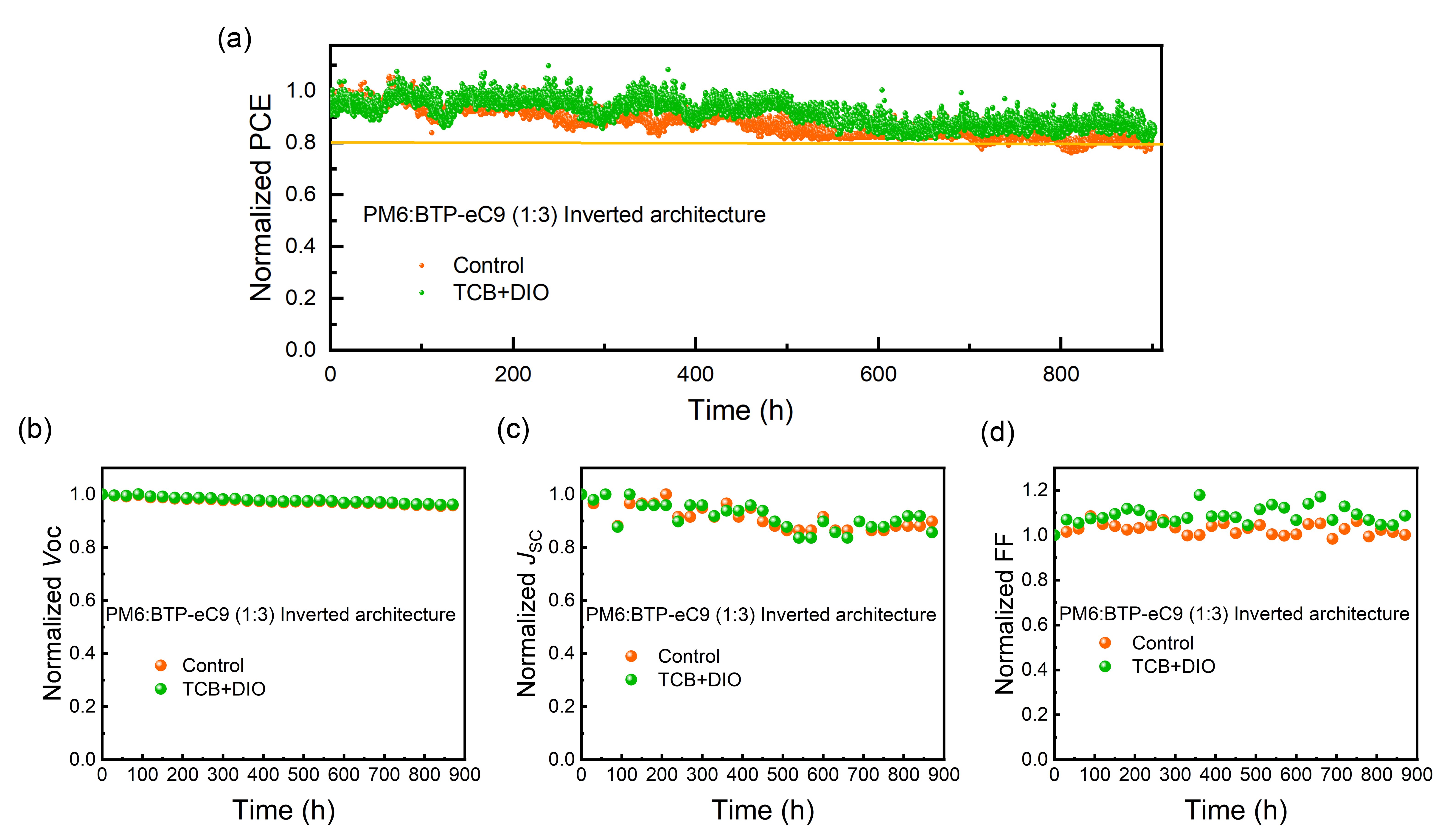
**

**Figure S29.** (a) MPPT tests of the unencapsulated PM6:BTP-eC9 (1:3) inverted devices. (b-d) Evolution of normalized *V*_OC_, *J*_SC_ and FF decay of PM6:BTP-eC9 (1:3) inverted devices before and after the addition of TCB and DIO.

**Table S1.** Film thicknesses, hole, and electron mobilities of IEICO-4F and Y6.

| Device | Film thickness  (nm) | Hole mobility  (10^-4^cm^2^V^-1^s^-1^) | Electron mobility  (10^-4^cm^2^V^-1^s^-1^) |
| --- | --- | --- | --- |
| IEICO-4F | 70 | 0.1±0.0 | 5.1±0.7 |
| Y6 | 80 | 4.8±0.8 | 7.2±0.5 |

**Table S2.** Photovoltaic performance of PTB7-Th:IEICO-4F and PTB7-Th:Y6 devices at various D/A ratios (1:2, 1:3, 1:4, 1:5, and 1:7).

| Materials | D/A ratio | *V*_OC_ ^[a]^  (V) | *J*_SC_ ^[a]^  (mA cm^-2^) | FF^[a]^  (%) | PCE ^[a]^  (%) | *J*_EQE_  (mA cm^-2^) |
| --- | --- | --- | --- | --- | --- | --- |
| PTB7-Th:IEICO-4F | 1:2 | 0.729  (0.726±0.002) | 21.3  (21.0±0.3) | 42.6  (42.0±0.4) | 6.6  (6.4±0.1) | 20.4 |
|  | 1:3 | 0.732  (0.725±0.004) | 21.0  (20.6±0.2) | 46.0  (45.5±0.8) | 7.1  (6.8±0.2) | 20.3 |
|  | 1:4 | 0.734  (0.735±0.003) | 19.5  (18.8±0.6) | 48.9  (47.8±0.8) | 7.0  (6.6±0.3) | 18.5 |
|  | 1:5 | 0.737  (0.733±0.004) | 18.2  (17.8±0.6) | 50.5  (49.6±1.1) | 6.8  (6.5±0.2) | 17.4 |
|  | 1:7 | 0.751  (0.747±0.004) | 14.4  (13.7±0.5) | 49.6  (48.8±1.1) | 5.4  (5.0±0.2) | 13.6 |
| PTB7-Th:Y6 | 1:2 | 0.705  (0.712±0.006) | 24.3  (23.8±0.4) | 55.7  (55.5±0.5) | 9.5  (9.4±0.1) | 23.6 |
|  | 1:3 | 0.710  (0.705±0.005) | 23.9  (23.5±0.7) | 57.5  (57.5±0.8) | 9.7  (9.5±0.2) | 23.0 |
|  | 1:4 | 0.713  (0.712±0.004) | 21.9  (21.8±0.4) | 60.9  (59.7±1.0) | 9.5  (9.3±0.2) | 21.0 |
|  | 1:5 | 0.717  (0.715±0.004) | 21.4  (21.2±0.4) | 61.9  (60.6±1.0) | 9.5  (9.2±0.1) | 20.6 |
|  | 1:7 | 0.731  (0.733±0.005) | 19.6  (18.9±0.7) | 59.1  (57.6±0.9) | 8.5  (8.0±0.3) | 18.0 |

^[a]^ The maximum and average values were obtained from 10 independent devices.

**Table S3.** *J*_MAX_, *J*_SC_ and the corresponding current density loss of PTB7-Th:IEICO-4F and PTB7-Th:Y6 devices at D/A ratios of 1:2 and 1:5.

| Materials | D/A ratio | *J*_MAX_  (mA cm^-2^) | *J*_SC_  (mA cm^-2^) | *J*_MAX_-*J*_SC_  (mA cm^-2^) |
| --- | --- | --- | --- | --- |
| PTB7-Th:IEICO-4F | 1:2 | 28.7 | 21.0 | 7.7 |
|  | 1:5 | 28.1 | 17.8 | 10.3 |
| PTB7-Th:Y6 | 1:2 | 30.5 | 23.8 | 6.7 |
|  | 1:5 | 28.5 | 21.2 | 7.3 |

**Table S4.** PCE of opaque devices, AVT of blend films, and their product of PTB7-Th:IEICO-4F and PTB7-Th:Y6 at various D/A ratios (1:2, 1:3, 1:4, 1:5, and 1:7).

| Materials | D/A ratio | PCE (%) | AVT (%) | PCE×AVT (%) |
| --- | --- | --- | --- | --- |
| PTB7-Th:IEICO-4F | 1:2 | 6.4 | 48.4 | 3.10 |
|  | 1:3 | 6.8 | 61.0 | 4.15 |
|  | 1:4 | 6.6 | 68.1 | 4.49 |
|  | 1:5 | 6.5 | 70.1 | 4.56 |
|  | 1:7 | 5.0 | 74.1 | 3.71 |
| PTB7-Th:Y6 | 1:2 | 9.4 | 56.4 | 5.30 |
|  | 1:3 | 9.5 | 59.4 | 5.64 |
|  | 1:4 | 9.3 | 65.5 | 6.09 |
|  | 1:5 | 9.2 | 70.5 | 6.48 |
|  | 1:7 | 8.0 | 72.5 | 5.80 |

**Table S5.** Film thicknesses, hole and electron mobilities of PTB7-Th:IEICO-4F and PTB7-Th:Y6 at D/A ratios of 1:2 and 1:5.

| Materials | D/A ratio | Film thickness  (nm) | Hole mobility ^[a]^  (10^-4^ cm^2^V^-1^s^-1^) | Electron mobility ^[a]^  (10^-4^ cm^2^V^-1^s^-1^) | *µ*_h_/*µ*_e_ |
| --- | --- | --- | --- | --- | --- |
| PTB7-Th:IEICO-4F | 1:2 | 130 | 4.6±1.6 | 0.6±0.1 | 7.7 |
|  | 1:5 | 90 | 9.1±0.7 | 3.6±0.2 | 2.5 |
| PTB7-Th:Y6 | 1:2 | 85 | 3.4±0.3 | 6.7±0.3 | 0.5 |
|  | 1:5 | 60 | 2.0±0.5 | 3.5±0.4 | 0.6 |

^[a]^ The average values were obtained from 10 independent devices.

**Table S6.** Parameters of PTB7-Th:IEICO-4F and PTB7-Th:Y6 devices at a D/A ratio of 1:2 via drift-diffusion simulations. The parameter N_c_ denotes the effective density of states, G represents the exciton generation rate, and K_BM_ refers to the bimolecular recombination rate.

| Materials | LUMO  (eV) | HOMO  (eV) | *N*_c_  (10^25^ m^-3^) | *G*  (10^28^ m^-3^ s^-1^) | *K*_BM_  (10^-17^ m^3^s^-1^) |
| --- | --- | --- | --- | --- | --- |
| PTB7-Th:IEICO-4F | 4.19 | 5.20 | 0.53 | 1.17 | 2 |
| PTB7-Th:Y6 | 4.10 | 5.20 | 1.95 | 1.94 | 23 |

**Table S7.** Photovoltaic performance of PTB7-Th:IEICO-4F and PTB7-Th:Y6 devices at a D/A ratio of 1:2 via drift-diffusion simulations.

| Materials | D/A ratio | *V*_OC_  (V) | *J*_SC_  (mA cm^-2^) | FF  (%) | PCE  (%) |
| --- | --- | --- | --- | --- | --- |
| PTB7-Th:IEICO-4F | 1:2 | 0.731 | 21.0 | 42.0 | 6.5 |
| PTB7-Th:Y6 | 1:2 | 0.710 | 23.7 | 55.3 | 9.3 |

**Table S8.** Film thicknesses and hole mobilities of PTB7-Th:Y6 at various D/A ratios (1:2, 1:3, 1:4, 1:5, and 1:7).

| Materials | D/A  ratio | Film thickness  (nm) | Hole mobility ^[a]^  (10^-4^ cm^2^V^-1^s^-1^) |
| --- | --- | --- | --- |
| PTB7-Th:Y6 | 1:2 | 85 | 3.4±0.3 |
|  | 1:3 | 75 | 3.2±0.1 |
|  | 1:4 | 70 | 2.5±0.4 |
|  | 1:5 | 60 | 2.0±0.5 |
|  | 1:7 | 55 | 1.7±0.3 |

^[a]^ The average values were obtained from 10 independent devices.

**Table S9.** Summary of calculated energy loss parameters of PTB7-Th:IEICO-4F and PTB7-Th:Y6 devices at D/A ratios of 1:2 and 1:5.

| Materials | D/A ratio | *E*_g_  (eV) | *qV*_OC_^SQ^  (eV) | *qV*_OC_^rad^  (eV) | *ΔE*_1_  (eV) | *ΔE*_2_  (eV) | *ΔE*_3_  (eV) | *qV*_OC_,cal  (eV) | *qV*_OC_  (eV) |
| --- | --- | --- | --- | --- | --- | --- | --- | --- | --- |
| PTB7-Th:IEICO-4F | 1:2 | 1.312 | 1.058 | 1.012 | 0.254 | 0.046 | 0.284 | 0.728 | 0.726 |
|  | 1:5 | 1.310 | 1.050 | 1.029 | 0.260 | 0.021 | 0.296 | 0.733 | 0.733 |
| PTB7-Th:Y6 | 1:2 | 1.371 | 1.115 | 1.039 | 0.256 | 0.076 | 0. 326 | 0.713 | 0.712 |
|  | 1:5 | 1.346 | 1.088 | 1.052 | 0.258 | 0.036 | 0. 336 | 0.716 | 0.715 |

**Table S10.** Parameters for calculation of the Flory-Huggins interaction parameters (*χ*) and ternary phase diagram. For the polymer, molar volume (*V*_m_) and the molecular weight (*M*_wt_) belong to the segment.

| Materials | *V*_m_  (cm^3^ mol^-1^) | *M*_wt_  (g mol^-1^) | *γ*_i_  (mN m^-1^) | *δ*_i_  (MPa^1/2^) |
| --- | --- | --- | --- | --- |
| PTB7-Th | 746 | 891.4 | 34.2 | 21.5 |
| Y6 | 1128.6 | 1451.9 | 38.2 | 22.7 |
| BTP-eC9 | 1226.6 | 1573.8 | 39.7 | 23.1 |
| IEICO-4F | 1459.7 | 1776.4 | 70.5 | 30.8 |
| CF | 80.5 | 133.4 | / | 18.9 |

**Table S11.** Flory-Huggins interaction parameters calculated by Hildebrand solubility parameters and the degree of polymerization (*N*_i_) for PTB7-Th, IEICO-4F, Y6, and BTP-eC9.

| Material systems | | | *χ*_12_ | *χ*_13_ | *χ*_23_ | *N*_2_ | *N*_3_ |
| --- | --- | --- | --- | --- | --- | --- | --- |
| 1 | 2 | 3 |  |  |  |  |  |
| CF | PTB7-Th | IEICO-4F | 0.56 | 4.95 | 3.16 | 731 | 19 |
| CF | PTB7-Th | Y6 | 0.56 | 0.81 | 0.39 | 731 | 15 |
| CF | PTB7-Th | BTP-eC9 | 0.56 | 0.91 | 0.42 | 731 | 16 |

**Table S12.** Initial component points (*φ*_CF_, *φ*_PTB7-Th_, *φ*_acceptor_) of PTB7-Th:IEICO-4F and PTB7-Th:Y6 devices at D/A ratios of 1:2 and 1:5, and PTB7-Th:BTP-eC9 devices at D/A ratios of 1:2 and 1:4.

| Materials | D/A ratio | Initial component point |
| --- | --- | --- |
| PTB7-Th:IEICO-4F | 1:2 | (0.982, 0.006, 0.012) |
|  | 1:5 | (0.985, 0.002, 0.012) |
| PTB7-Th:Y6 | 1:2 | (0.989, 0.004, 0.007) |
|  | 1:5 | (0.991, 0.001, 0.007) |
| PTB7-Th:BTP-eC9 | 1:2 | (0.989, 0.004, 0.007) |
|  | 1:4 | (0.991, 0.002, 0.007) |

**Table S13.** The fitted peak location, d-spacing, full width at half maximum (FWHM), coherence length, and peak area from GIWAXS patterns of PTB7-Th:IEICO-4F and PTB7-Th:Y6 blend films at D/A ratios of 1:2 and 1:5.

| Conditions | D/A ratio | Location  (Å^-1^) | d-spacing  (Å) | FWHM  (Å^-1^) | CL  (nm) | peak area |
| --- | --- | --- | --- | --- | --- | --- |
| PTB7-Th:IEICO-4F | 1:2 | 0.315 | 19.95 | 0.050 | 11.37 | 0.76 |
|  |  | 1.839 | 3.42 | 0.198 | 2.85 | 1.01 |
|  | 1:5 | 0.320 | 19.65 | 0.042 | 13.47 | 1.43 |
|  |  | 1.858 | 3.38 | 0.183 | 3.09 | 1.56 |
| PTB7-Th:Y6 | 1:2 | 0.277 | 22.66 | 0.114 | 4.95 | 1.48 |
|  |  | 1.755 | 3.58 | 0.265 | 2.13 | 1.89 |
|  | 1:5 | 0.277 | 22.71 | 0.108 | 5.26 | 0.36 |
|  |  | 1.774 | 3.54 | 0.268 | 2.11 | 0.70 |

**Table S14.** Photovoltaic performance of PTB7-Th:BTP-eC9 devices at various D/A ratios (1:2, 1:3, 1:4, 1:5, and 1:7).

| Materials | D/A ratio | *V*_OC_^[a]^  (V) | *J*_SC_ ^[a]^  (mA cm^-2^) | FF ^[a]^  (%) | PCE ^[a]^  (%) | *J*_EQE_  (mA cm^-2^) |
| --- | --- | --- | --- | --- | --- | --- |
| PTB7-Th:BTP-eC9 | 1:2 | 0.710  (0.706±0.004) | 23.1  (22.7±0.6) | 64.9  (64.7±0.9) | 10.7  (10.4±0.2) | 22.5 |
|  | 1:3 | 0.706  (0.707±0.004) | 22.1  (21.8±0.3) | 66.3  (66.2±0.4) | 10.4  (10.2±0.1) | 21.1 |
|  | 1:4 | 0.709  (0.709±0.002) | 20.6  (20.4±0.2) | 68.4  (68.3±0.4) | 10.0  (9.9±0.1) | 20.2 |
|  | 1:5 | 0.716  (0.712±0.004) | 19.9  (19.6±0.5) | 64.7  (64.4±0.7) | 9.2  (9.0±0.2) | 19.1 |
|  | 1:7 | 0.721  (0.723±0.003) | 18.7  (18.5±0.2) | 54.5  (54.3±1.0) | 7.4  (7.3±0.1) | 18.1 |

^[a]^ The maximum and average values were obtained from 10 independent devices.

**Table S15.** The fitted peak location, d-spacing, FWHM, coherence length and peak area from GIWAXS patterns of PTB7-Th:BTP-eC9 blend films at D/A ratios of 1:2 and 1:4 with and without the addition of TCB and DIO.

| Materials | D/A  ratio | Location  (Å^-1^) | d-spacing  (Å) | FWHM  (Å^-1^) | CL  (nm) | peak  area |
| --- | --- | --- | --- | --- | --- | --- |
| PTB7-Th:BTP-eC9 | 1:2 | 0.38 | 16.67 | 0.14 | 4.10 | / |
|  |  | 1.74 | 3.61 | 0.34 | 1.66 | / |
| PTB7-Th:BTP-eC9 | 1:4 | 0.40 | 15.63 | 0.10 | 5.54 | 2.35 |
|  |  | 1.76 | 3.57 | 0.35 | 1.61 | / |
| PTB7-Th:BTP-eC9  TCB | 1:4 | 0.41 | 15.44 | 0.10 | 6.98 | 2.71 |
|  |  | 1.76 | 3.56 | 0.34 | 1.68 | / |
| PTB7-Th:BTP-eC9  DIO | 1:4 | 0.41 | 15.44 | 0.08 | 6.90 | 1.82 |
|  |  | 1.77 | 3.56 | 0.35 | 1.61 | / |
| PTB7-Th:BTP-eC9  TCB+DIO | 1:4 | 0.40 | 15.59 | 0.10 | 6.73 | 2.44 |
|  |  | 1.77 | 3.55 | 0.35 | 1.60 | / |

**Table S16.** PCE of opaque devices, AVT of blend films, and their product of PTB7-Th:BTP-eC9 at various D/A ratios (1:2, 1:3, 1:4, 1:5, and 1:7) and the 1:4 ratio with the addition of TCB and DIO.

| Materials | D/A ratio | PCE (%) | AVT (%) | PCE×AVT (%) |
| --- | --- | --- | --- | --- |
| PTB7-Th:BTP-eC9 | 1:2 | 10.4 | 56.2 | 5.84 |
|  | 1:3 | 10.2 | 60.4 | 6.16 |
|  | 1:4 | 9.9 | 65.8 | 6.51 |
|  | 1:5 | 9.0 | 70.0 | 6.20 |
|  | 1:7 | 7.4 | 72.4 | 5.36 |
| PTB7-Th:BTP-eC9  (TCB+DIO) | 1:4 | 11.3 | 65.4 | 7.39 |

**Table S17.** Photovoltaic performance of PTB7-Th:BTP-eC9 devices at a D/A ratio of 1:4 with and without the addition of TCB (10 mg/ml) and DIO (0.25%).

| Materials | *V*_OC_ ^[a]^  (V) | *J*_SC_ ^[a]^  (mA cm^-2^) | FF ^[a]^  (%) | PCE ^[a]^  (%) |  |
| --- | --- | --- | --- | --- | --- |
| Control | 0.709  (0.709±0.002) | 20.6  (20.4±0.2) | 68.4  (68.3±0.4) | 10.0  (9.9±0.1) | |
| 10mg/ml TCB | 0.716 (0.714±0.002) | 22.8  (22.5±0.4) | 65.4  (65.1±0.9) | 10.7  (10.5±0.2) | |
| 0.25% DIO | 0.706  (0.705±0.004) | 22.2  (21.8±0.6) | 70.8  (70.5±1.1) | 11.1  (10.8±0.2) | |
| 10mg/mlTCB+0.25%DIO | 0.700  (0.700±0.005) | 23.2  (23.1±0.4) | 71.0  (70.2±0.4) | 11.5  (11.3±0.2) | |

^[a]^ The maximum and average values were obtained from 10 independent devices.

**Table S18.** Film thicknesses, hole and electron mobilities of PTB7-Th:BTP-eC9 device at a D/A ratio of 1:4 with and without the addition of TCB and DIO

| Materials | D/A ratio | Film thickness  (nm) | Hole mobility ^[a]^  (10^-4^ cm^2^V^-1^s^-1^) | Electron mobility ^[a]^  (10^-4^ cm^2^V^-1^s^-1^) |
| --- | --- | --- | --- | --- |
| PTB7-Th:BTP-eC9 | 1:4 | 65 | 8.0±1.04 | 6.7±0.27 |
| PTB7-Th:BTP-eC9  TCB+DIO | 1:4 | 65 | 9.4±0.38 | 7.2±0.75 |

^[a]^ The average values were obtained from 10 independent devices.

**Table S19.** Parameters for calculating the carrier drift length (*L*_dr_).

| Conditions | *d*  (nm) | *J*_MAX_  (mA cm^-2^) | *γ*_pre_  (10^-2^) | FOM *θ*  (10^-3^) | *L*_dr_  (nm) |
| --- | --- | --- | --- | --- | --- |
| PTB7-Th:BTP-eC9 1:4 | 65 | 27.4 | 2.84 | 3.9 | 1044 |
| PTB7-Th:BTP-eC9 1:4  TCB+DIO | 65 | 27.5 | 1.48 | 1.9 | 1507 |

**Table S20.** Photovoltaic performance of PM6:BTP-eC9 devices at a D/A ratio of 1:3 with and without the addition of TCB and DIO.

| Conditions | | *V*_OC_ ^[a]^  (V) | *J*_SC_ ^[a]^  (mA cm^-2^) | FF ^[a]^  (%) | PCE ^[a]^  (%) |
| --- | --- | --- | --- | --- | --- |
| TCB  (mg/ml) | DIO  (%) |  |  |  |  |
| 0 | 0 | 0.868  (0.865±0.005) | 26.0  (25.5±0.2) | 75.2  (75.8±0.5) | 16.9  (16.8±0.1) |
| 5 | 0 | 0.841  (0.840±0.003) | 26.2  (26.0±0.3) | 77.1  (76.9±0.4) | 17.0  (16.8±0.1) |
|  | 0.15 | 0.840  (0.841±0.001) | 26.1  (25.8±0.3) | 79.4  (78.9±0.6) | 17.4  (17.1±0.2) |
|  | 0.25 | 0.845  (0.845±0.003) | 26.7  (26.3±0.4) | 78.3  (78.6±0.7) | 17.6  (17.4±0.2) |
|  | 0.40 | 0.824  (0.821±0.001) | 24.2  (23.7±0.5) | 75.7  (75.8±0.5) | 15.1  (14.8±0.3) |
| 0 | 0.25 | 0.829  (0.830±0.005) | 26.4  (25.7±0.5) | 77.6  (78.0±0.5) | 17.0  (16.7±0.2) |
| 1 |  | 0.840  (0.843±0.002) | 24.5  (24.2±0.4) | 79.0  (78.6±0.4) | 16.2  (16.0±0.2) |
| 5 |  | 0.845  (0.845±0.003) | 26.7  (26.3±0.4) | 78.3  (78.6±0.7) | 17.6  (17.4±0.2) |
| 10 |  | 0.837  (0.831±0.004) | 22.4  (22.1±0.4) | 77.2  (75.9±1.0) | 14.5  (14.0±0.3) |

^[a]^ The maximum and average values were obtained from 10 independent devices.

**Table S21.** Photovoltaic performance of D18:BTP-eC9 devices at a D/A ratio of 1:3 with and without the addition of TCB and DIO.

| Materials | *V*_OC_ ^[a]^  (V) | *J*_SC_ ^[a]^  (mA cm^-2^) | FF ^[a]^  (%) | PCE ^[a]^  (%) | |
| --- | --- | --- | --- | --- | --- |
| Control | 0.881  (0.875±0.005) | 25.7  (25.7±0.3) | 74.7  (74.7±0.5) | 16.9  (16.8±0.1) |  |
| 5mg/mlTCB+0.1%DIO | 0.862  (0.861±0.002) | 26.7  (26.3±0.3) | 76.0  (76.0±0.6) | 17.5  (17.2±0.2) |  |

^[a]^ The maximum and average values were obtained from 10 independent devices.

**Table S22.** Photovoltaic performance of PM6:BTP-eC9 (1:3) ST-OSCs with various Ag thicknesses (10nm, 13nm, 15nm, 18nm, and 20nm).

| Materials | Ag thickness (nm) | *V*_OC_ ^[a]^  (V) | *J*_SC_^[a]^  (mA cm^-2^) | FF ^[a]^  (%) | PCE ^[a]^  (%) | AVT  (%) | LUE  (%) |
| --- | --- | --- | --- | --- | --- | --- | --- |
| PM6:BTP-eC9  1:3 | 10 | 0.763  (0.770±0.012) | 16.4  (16.3±0.2) | 64.0  (63.1±0.6) | 8.0  (7.9±0.1) | 27.5 | 2.20 |
|  | 13 | 0.802  (0.803±0.023) | 19.5  (18.9±0.5) | 66.7  (66.2±0.8) | 10.5  (10.1±0.3) | 25.4 | 2.67 |
|  | 15 | 0.839  (0.836±0.003) | 20.0  (19.7±0.5) | 69.2  (68.4±0.7) | 11.6  (11.3±0.2) | 23.1 | 2.68 |
|  | 18 | 0.838  (0.820±0.017) | 21.4  (21.2±0.3) | 71.5  (71.5±0.9) | 12.8  (12.4±0.3) | 22.3 | 2.85 |
|  | 20 | 0.852  (0.846±0.006) | 22.3  (21.7±0.4) | 72.1  (71.3±0.5) | 13.7  (13.1±0.4) | 16.6 | 2.28 |

^[a]^ The maximum and average values were obtained from 10 independent devices.

**Table S23.** Photovoltaic performance of PM6:BTP-eC9 (1:3) ST-OSCs with and without optically modulation and dual-additive treatment.

| Conditions | *V*_OC_^[a]^  (V) | *J*_SC_^[a]^  (mA cm^-2^) | FF^[a]^  (%) | PCE^[a]^  (%) | *J*_EQE_  (mA cm^-2^) | AVT  (%) | LUE  (%) |
| --- | --- | --- | --- | --- | --- | --- | --- |
| 18nm Ag | 0.838  (0.820±0.017) | 21.4  (21.2±0.3) | 71.5  (71.5±0.9) | 12.8  (12.4±0.3) | 20.5 | 22.3 | 2.85 |
| 18nm Ag  + optically modulation | 0.857  (0.851±0.005) | 20.4  (19.9±0.4) | 72.3  (71.2±0.7) | 12.6  (12.1±0.3) | 19.5 | 35.1 | 4.42 |
| 18nm Ag  + optically modulation  + dual-additive treatment | 0.824  (0.823±0.004) | 21.9  (20.6±1.0) | 75.3  (74.6±0.5) | 13.6  (12.7±0.6) | 21.1 | 34.3 | 4.67 |

^[a]^ The maximum and average values were obtained from 10 independent devices.

**Table S24.** Color coordinates of PM6:BTP-eC9 (1:3) ST-OSCs with and without optically modulation and dual-additive treatment.

| Conditions | color coordinates (*x*, *y*) |
| --- | --- |
| 18nm Ag | (0.2732, 0.2862) |
| 18nm Ag + optically modulation | (0.3161, 0.3358) |
| 18nm Ag + optically modulation+ dual-additive treatment | (0.3191, 0.3425) |

**References:**

[1] Y. Firdaus, V. M. Le Corre, S. Karuthedath, W. Liu, A. Markina, W. Huang, S. Chattopadhyay, M. M. Nahid, M. I. Nugraha, Y. Lin, A. Seitkhan, A. Basu, W. Zhang, I. McCulloch, H. Ade, J. Labram, F. Laquai, D. Andrienko, L. J. A. Koster, T. D. Anthopoulos, *Nat. Commun.* **2020**, *11*, 5220.

[2] B. Siegmund, M. T. Sajjad, J. Widmer, D. Ray, C. Koerner, M. Riede, K. Leo, I. D. W. Samuel, K. Vandewal, *Adv. Mater.* **2017**, *29*, 1604424.

[3] Y. Tang, H. Zheng, X. Zhou, Z. Tang, W. Ma, H. Yan, *Energ. Environ. Sci.* **2023**, *16*, 653.

[4] Y. Tang, Y. Cui, R. Zhang, W. Xue, W. Ma, H. Yan, *Adv. Energy Mater.* **2024**, *14*, 2303799.

[5] S. Yoo, B. Domercq, B. Kippelen, *Appl. Phys. Lett.* **2004**, *85*, 5427.

[6] J. Benduhn, K. Tvingstedt, F. Piersimoni, S. Ullbrich, Y. Fan, M. Tropiano, K. A. McGarry, O. Zeika, M. K. Riede, C. J. Douglas, S. Barlow, S. R. Marder, D. Neher, D. Spoltore, K. Vandewal, *Nat. Energy* **2017**, *2*, 17053.

[7] J. Liu, S. Chen, D. Qian, B. Gautam, G. Yang, J. Zhao, J. Bergqvist, F. Zhang, W. Ma, H. Ade, O. Inganäs, K. Gundogdu, F. Gao, H. Yan, *Nat. Energy* **2016**, *1*, 16089.

[8] S. Kouijzer, J. J. Michels, M. van den Berg, V. S. Gevaerts, M. Turbiez, M. M. Wienk, R. A. J. Janssen, *J. Am. Chem. Soc.* **2013**, *135*, 12057.

[9] J.-H. Kim, A. Gadisa, C. Schaefer, H. Yao, B. R. Gautam, N. Balar, M. Ghasemi, I. Constantinou, F. So, B. T. O'Connor, K. Gundogdu, J. Hou, H. Ade, *J. Mater. Chem. A* **2017**, *5*, 13176.

[10] Q. Xue, R. Xia, C. J. Brabec, H.-L. Yip, *Energ. Environ. Sci.* **2018**, *11*, 1688.

[11] M. Żenkiewicz, *J. Achiev. Mater. Manuf. Eng.* **2007**. *24*, 137-145.

[12] X. Zhao, B. Mi, Z. Gao, W. Huang, *Sci. China Phys. Mech.* **2011**, *54*, 375.

[13] S. Gao, L. Bu, Z. Zheng, X. Wang, W. Wang, L. Zhou, J. Hou, G. Lu, *AIP Adv.* **2017**, *7*, 045312.

[14] G. F. Burkhard, E. T. Hoke, M. D. McGehee, *Adv. Mater.* **2010**, *22*, 3293.

[15] M. Koopmans, V. Corre, L. Koster, *Journal of Open Source Software* **2022**, *7*, 3727.

[16] A. These, L. J. A. Koster, C. J. Brabec, V. M. Le Corre, *Adv. Energy Mater.* **2024**, *14*, 2400055.

[17] Y. Zhang, C. Zhong, G. Cai, Y. Li, J. Wang, H. Lu, B. Jia, X. Lu, Y. Lin, X. Zhan, X. Chen, *J. Mater. Chem. C* **2024**, *12*, 1860.

[18] Z. Wang, K. Gao, Y. Kan, M. Zhang, C. Qiu, L. Zhu, Z. Zhao, X. Peng, W. Feng, Z. Qian, X. Gu, A. K. Y. Jen, B. Z. Tang, Y. Cao, Y. Zhang, F. Liu, *Nat. Commun.* **2021**, *12*. 332.

[19] D. Lin, Y. Huang, *Int. J. Pharm.* **2010**, *399*, 109.

[20] L. J. A. Koster, M. Kemerink, M. M. Wienk, K. Maturová, R. A. J. Janssen, *Adv. Mater.* **2011**, *23*, 1670.

[21] N. Tokmoldin, J. Vollbrecht, S. M. Hosseini, B. Sun, L. Perdigón‐Toro, H. Y. Woo, Y. Zou, D. Neher, S. Shoaee, *Adv. Energy Mater.* **2021**. *11*. 2100804.

[22] D. Bartesaghi, I. d. C. Pérez, J. Kniepert, S. Roland, M. Turbiez, D. Neher, L. J. A. Koster, *Nat. Commun.* **2015**, *6*, 7083.
